# Supplementary material for: eIF4E1b is a non-canonical eIF4E protecting maternal dormant mRNAs
Source: EMBO Rep. 2023 Dec 14;25(1):23. doi: 10.1038/s44319-023-00006-4 (PMC10883267; doi:10.1038/s44319-023-00006-4)
Supplement: Supplementary file 1 — Appendix [file 44319_2023_6_MOESM1_ESM.pdf]

eIF4E1b is a non-canonical eIF4E protecting maternal dormant mRNAs

Laura Lorenzo-Orts, Marcus Strobl, Benjamin Steinmetz, Friederike Leesch, Carina Pribitzer, Josef Roehsner, Michael Schutzbier, Gerhard Dürnberger, Andrea Pauli

Appendix

Table of contents:

Appendix Figure S1 ______ 2

Appendix Figure S2 ______ 3

Appendix Figure S3 ______ 4

Appendix Figure S4 ______ 5

Appendix Figure S5 ______ 6

Appendix Table S1 ______ 7

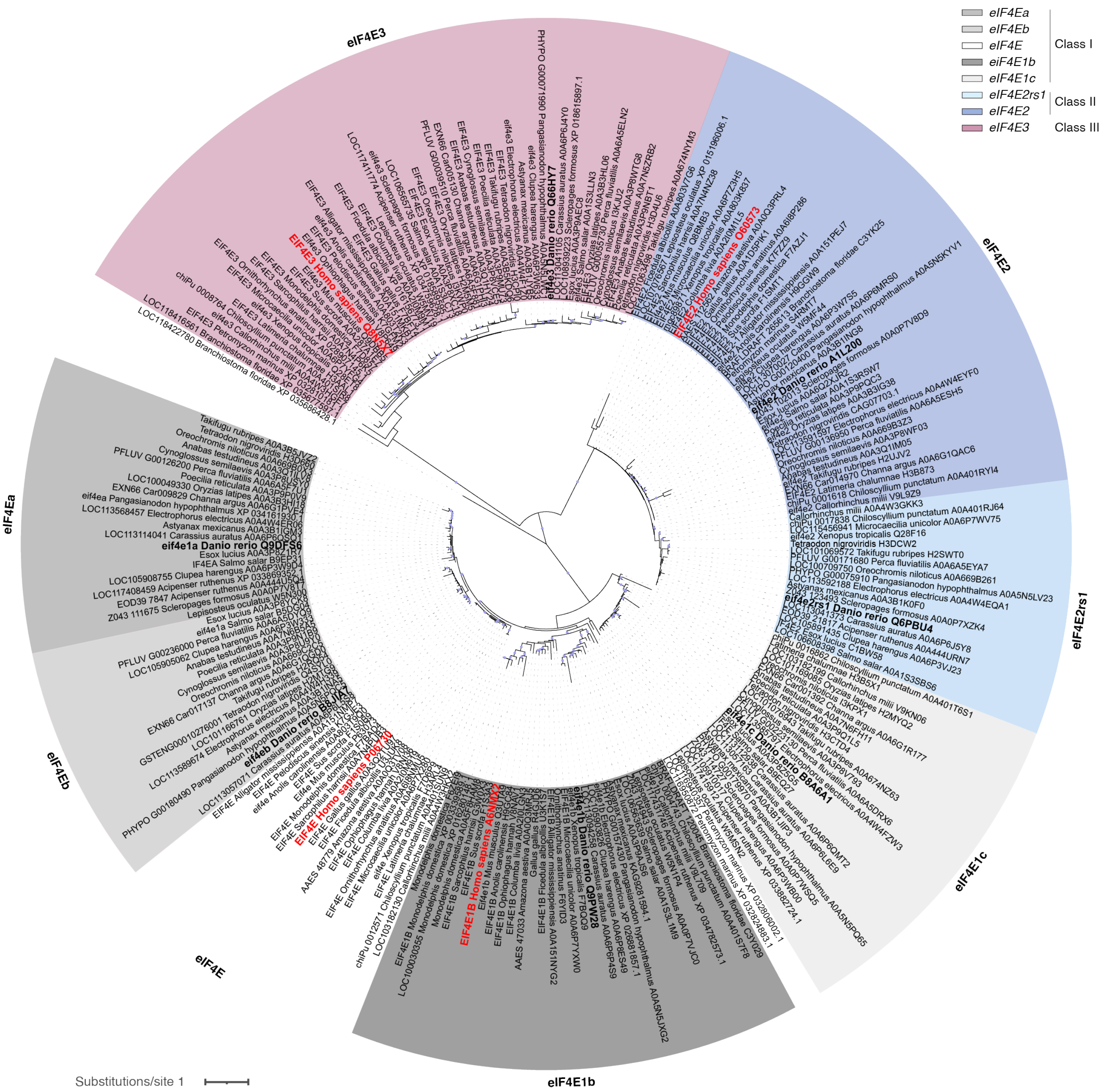

Appendix Figure S1. Phylogenetic analysis of vertebrate eIF4Es. Phylogenetic tree showing 3 major branches that correspond to 3 eIF4E classes: class I with eIF4Ea, eIF4Eb, eIF4E, eIF4E1b and eIF4E1c, class II with eIF4E2rs1and eIF4E2, and class III with eIF4E3. Branches that are supported by an ultrafast bootstrap (UFBoot) value ≥ 95% are indicated by a grey dot. Branch lengths represent the inferred number of amino acid substitutions per site, and branch labels are composed of gene name (if available), genus, species, and accession number. Zebrafish genes (black) and human genes (red) are highlighted in bold.

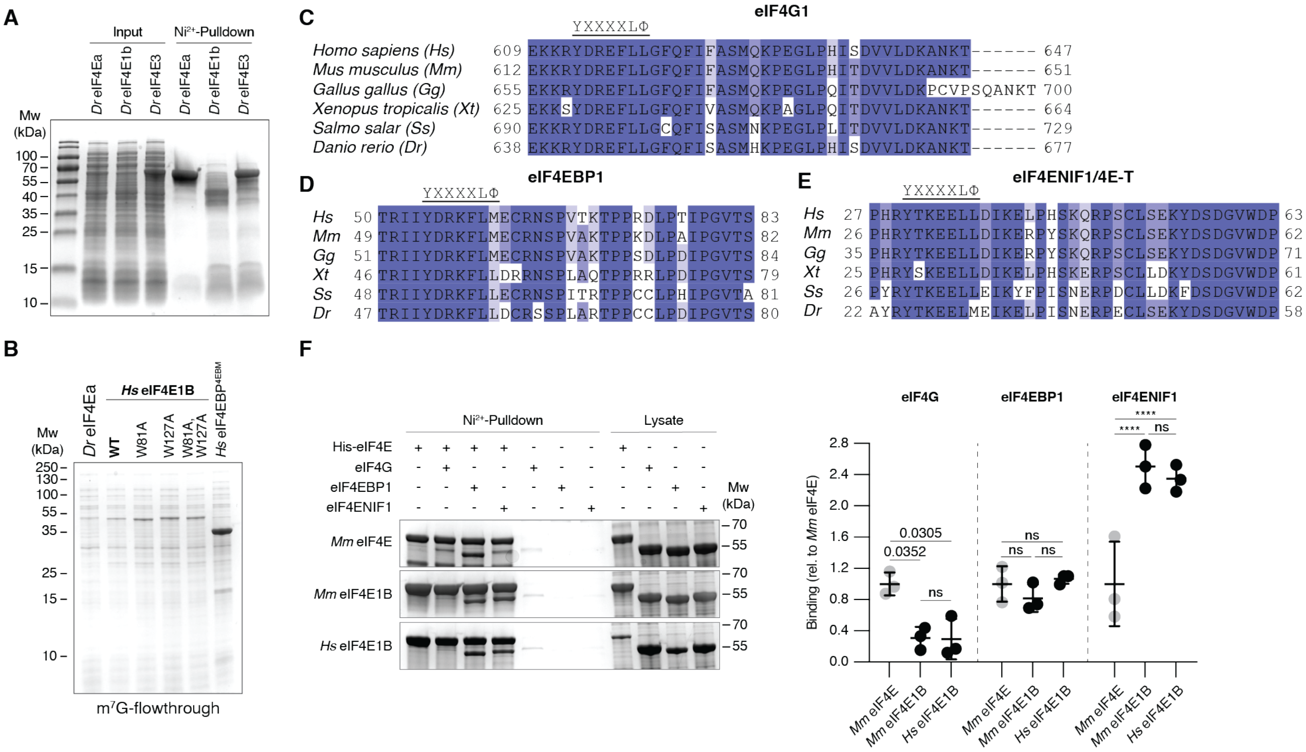

Appendix Figure S2. Human eIF4E1B interacts with the eIF4E-binding motifs of human eIF4EBP1 and eIF4ENIF1, which are conserved across vertebrates.

A Coomassie stained gel of a pulldown assay using Ni^{2+} beads and bacterial lysates containing His- and MBP-tagged zebrafish (*Danio rerio*, *Dr*) eIF4Ea, eIF4E1b and eIF4E3. Inputs corresponding to the soluble fractions of the lysates are shown on the left; elutions are shown on the right.

B Coomassie stained gel of flow-through fractions from an immunoprecipitation assay using m^7G-coated beads and *E. coli* lysates containing eIF4Es and eIF4EBP1.

C-E Amino acid alignments of the eIF4E-binding motifs (4EBMs) of eIF4G1 (*C*), eIF4EBP1 (*D*) and eIF4ENIF1/4E-T (*E*) from six vertebrate species. The species abbreviations are shown in *C*. The canonical YXXXXLΦ eIF4E-binding motif is indicated (X = any amino acid; Φ = hydrophobic residue).

F Human and mouse eIF4E1Bs show similar affinities for eIF4E-binding proteins. Representative Coomassie-stained gels of in vitro pulldown assays with bacteria lysates is shown on the left. Quantification are shown on the right (lines indicate mean with SD; n = 3 independent experiments; mouse eIF4E and eIF4E1B data is also shown in **Fig 2D**). Statistical analysis was performed with two-way ANOVA followed by Sidak's multiple comparisons test (ns: non-significant).

Data information: In *A* and *F*, predicted molecular weights (in kDa) are: *Dr* eIF4Ea, *Dr* eIF4E1b, *Mm* eIF4E1B and *Hs* eIF4E1B = 65; *Mm* eIF4E = 66; *Dr* eIF4E3 = 64; *Hs* eIF4G$^{[4EBM]}$ and *Hs* eIF4ENIF1$^{[4EBM]}$ = 53; *Hs* eIF4EBP1$^{[4EBM]}$ = 52.

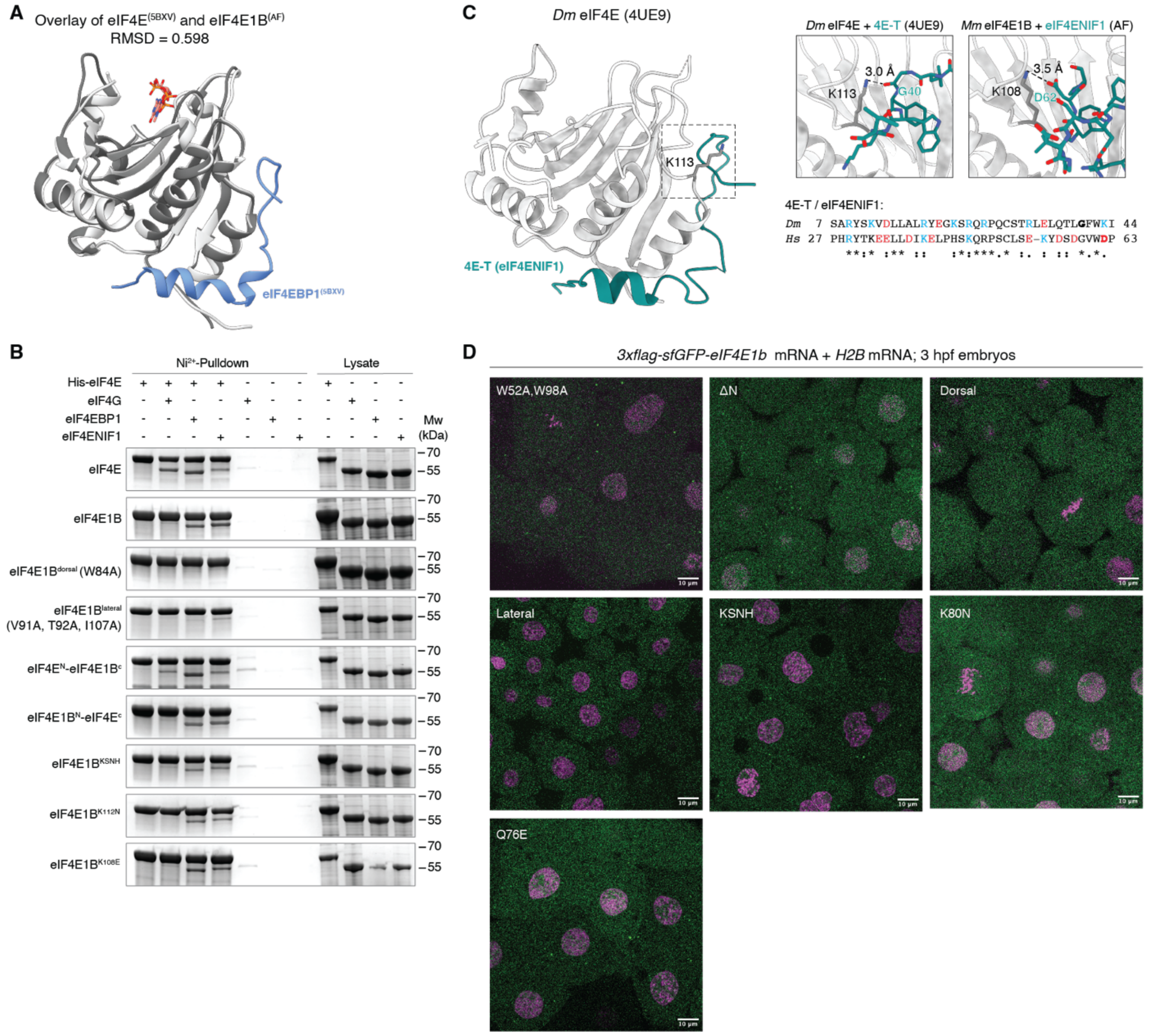

Appendix Figure S3. Interrogating the interaction between eIF4E1b and EIF4NIF1.

A Superimposed structures of mouse eIF4E (PDB-5BXV, Sekiyama *et al*, 2015) and mouse eIF4E1B (predicted by AlphaFold, AF). RMSD = root-mean-square deviation.

B Representative Coomassie-stained gels from pulldown assays with mouse eIF4E1B mutants. Quantifications are shown in **Fig 3D**. Predicted molecular weights (in kDa) are: *Mm* eIF4E1B, *Mm* eIF4E^{N}-eIF4E1B^{C} and *Mm* eIF4E1B^{N}-eIF4E^{C} = 65; *Mm* eIF4E = 66; *Hs* eIF4G$^{[4EBM]}$ and *Hs* eIF4ENIF1$^{[4EBM]}$ = 53; *Hs* eIF4EBP1$^{[4EBM]}$ = 52. 4EBM = eIF4E-binding motif.

C Structural representation of *Drosophila melanogaster* (*Dm*) eIF4E in complex with 4E-T (PDB-4UE9, Peter *et al*, 2015). The nitrogen of Lys113 of *Dm* eIF4E (corresponding to Lys108 of mouse eIF4E1B) forms a main-chain contact (dashed line) with the carbonyl oxygen of Gly40 of *Dm* 4E-T. In contrast, Lys108 of mouse eIF4E1B is predicted to interact with Asp62 of eIF4ENIF1 in AF. An alignment of *Dm* 4E-T and *Hs* eIF4ENIF1 binding motifs is shown on the bottom right: * = identical residues; : = similar residues; red: acid; blue: basic; bold: interacting with Lys113 or Lys108 of *Dm* eIF4E or *Mm* eIF4E1B, respectively.

D Confocal microscopy images of 1k-cell embryos transiently expressing eIF4E1b mutant proteins from mRNAs injected at the 1-cell stage. Nuclei were labeled with *H2B-RFP* mRNA. Quantification of the number of GFP-positive foci is shown in **Fig 3H**. Scale bars = 10 µm.

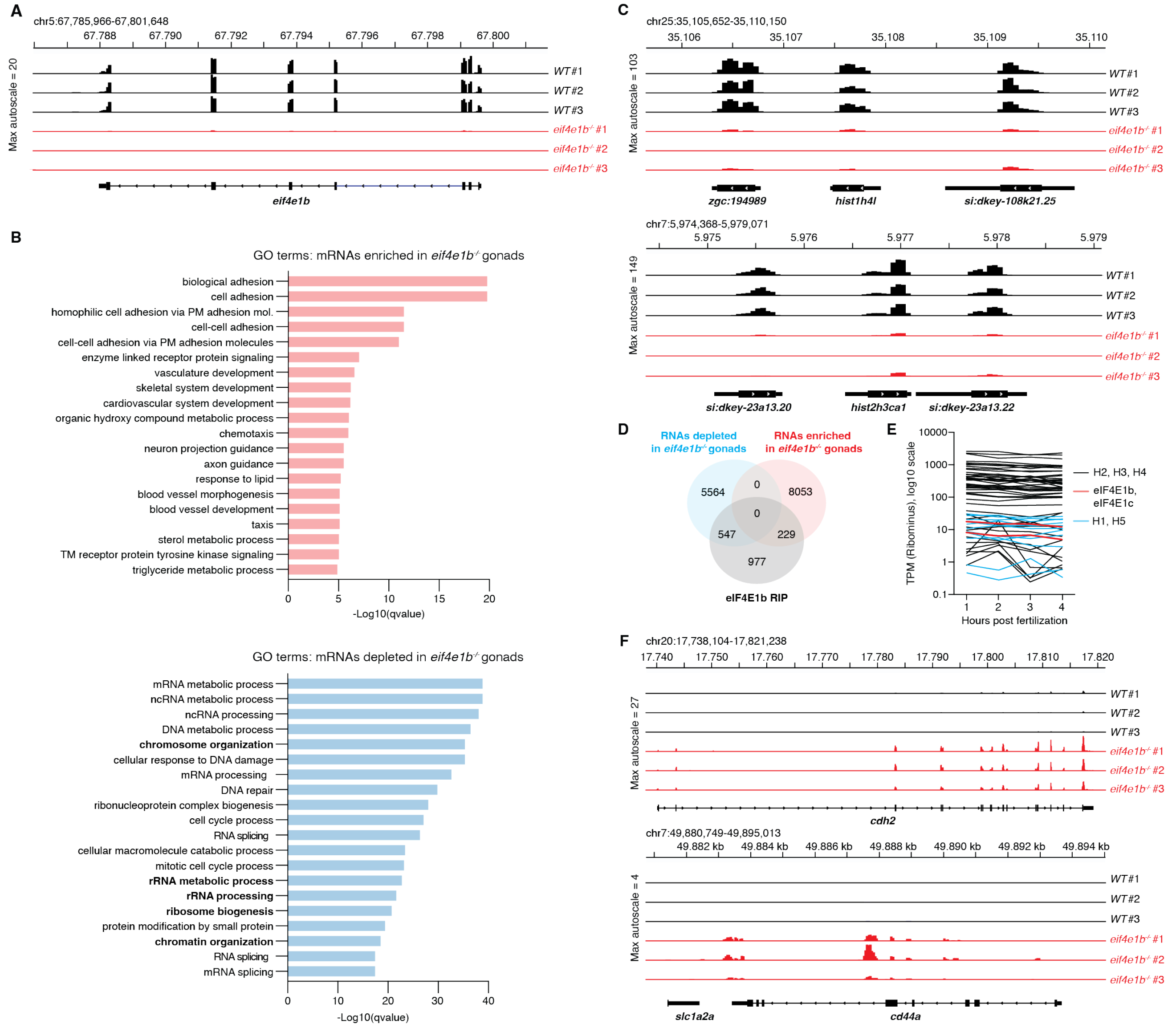

Appendix Figure S4. Transcriptomic analyses of *eif4e1b* mutant and wild-type gonads.
A RNA-seq reads for the *eif4e1b* transcript in *eif4e1b* mutant and wild-type gonads isolated from juvenile fish with high expression of *ziwi:GFP*.
B GO term analysis of up- (top, in red) and down-regulated (bottom, in blue) mRNAs in *eif4e1b* mutant gonads with high *ziwi:GFP* expression. GO terms that are also enriched in the eIF4E1b RIP are highlighted in bold.
C Sequencing reads for 6 histone mRNAs in *eif4e1b* mutant and wild-type gonads isolated from juvenile fish with a high expression of *ziwi:GFP*.
D Venn diagram showing the overlap between mRNAs down- or up-regulated in *eif4e1b* mutant gonads with mRNAs enriched in the eIF4E1b RIP at 1.25 hours post fertilization.
E Histone mRNA abundance (in transcripts per million, TPM) during the first four hours post fertilization according to published rRNA-depleted RNA-seq data (Cabrera-Quio *et al*, 2021).
F RNA-seq reads for cell adhesion molecule mRNAs, such as *cdh2* (top) and *cd44a* (bottom), in *eif4e1b* mutant and wild-type gonads of juvenile fish with high *ziwi:GFP* expression.

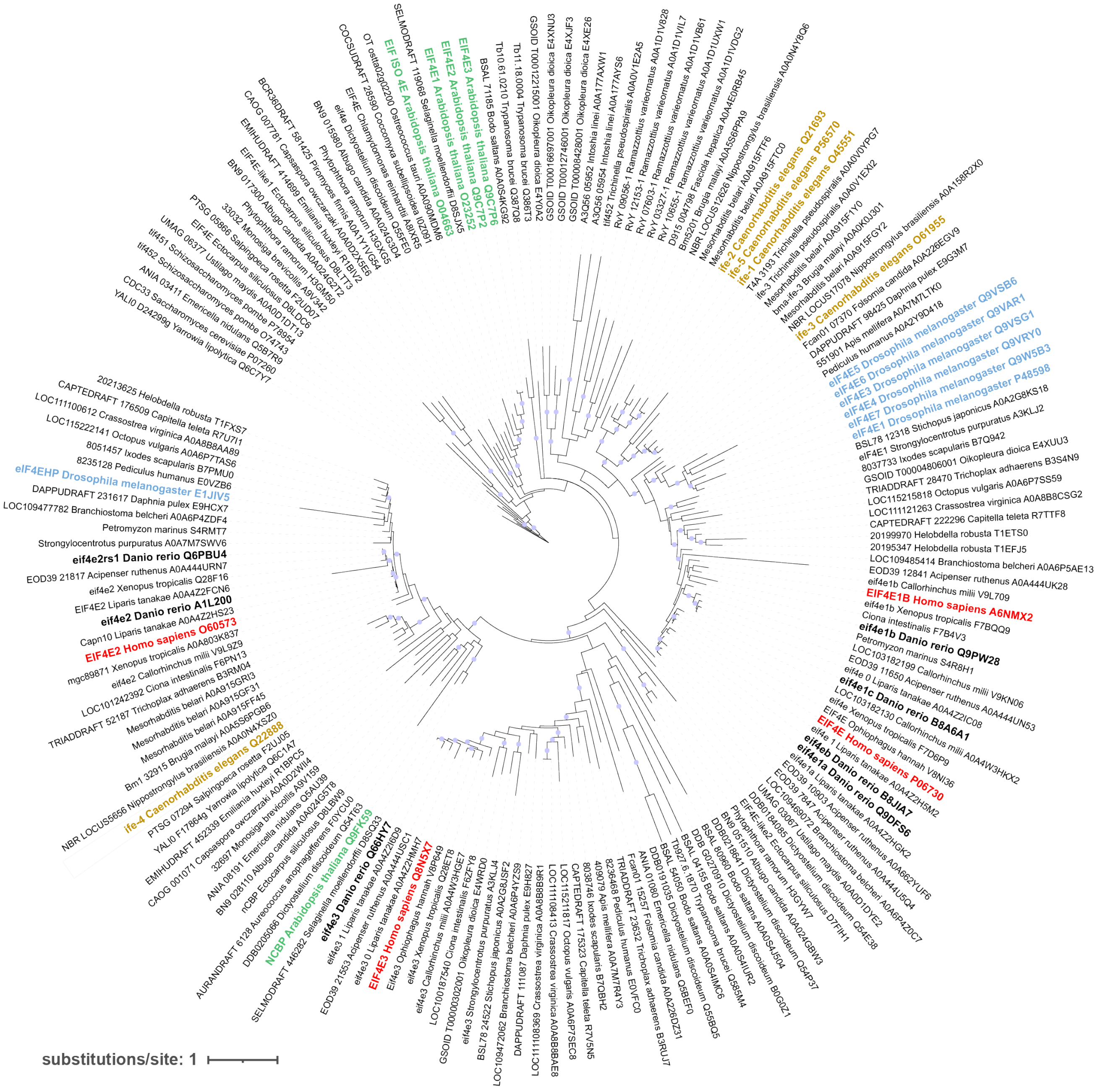

Appendix Figure S5. Phylogenetic analysis of eIF4E proteins in eukaryotes. Phylogenetic tree of eIF4E proteins from different species, including protists, fungi, plants, and animals (invertebrates and vertebrates). Grey dots indicate branches that are supported by an ultrafast bootstrap (UFBoot) value ≥ 95%. Branch lengths represent the inferred number of amino acid substitutions per site. Labels consist of gene name (if available), genus, species, and accession number. eIF4E proteins from specific organisms are highlighted in bold: zebrafish (black), human (red), *Drosophila melanogaster* (blue), *Caenorhabditis elegans* (goldenrod), and *Arabidopsis thaliana* (green).

Appendix Table S1. List of primers used for RT-qPCR or RT-PCR in this study. Genes dysregulated in *eIF4E1b* or *eIF4E1c* overexpressing (OE) embryos are highlighted in violet (down) or blue (up).

	Name	Sequence
qPCR in eIF4E1c OE embryos	si:ch211-226h8.4-F	TGTGTTCTCTGATCCCTGTTATG
	si:ch211-226h8.4-R	GAGACGCTGGCAGAGAAAT
	zgc:112146-F	GCAACACTCGACACAAGAGA
	zgc:112146-R	TTCCAGTGTAGTCCACAAATCC
	gcdhb-F	CCCGTTTGGGTGTCTGAATAA
	gcdhb-R	GTCCAGTGTGTACTGTCTTGTG
	commd3-F	AAGTGTCGAGAGAGCAACAC
	commd3-R	ACAACCACAGCACCAGATAATA
	aldh9a1b-F	CAAGGACAGGTGTGCAGTAA
	aldh9a1b-R	GAGGGTCTCCAATGCTGATAG
	man2b2-F	GGGCCGATTTCAGACAACTA
	man2b2-R	TCCTGGCACAATCTCCATAAC
	si:ch73-366l1.5-F	TCCACCAAGTTTGAGAAGAGATT
	si:ch73-366l1.5-R	CAGTGATCCTTGAGGCTGATATT
qPCR in eIF4E1b OE embryos	zgc:103559-F	CCTCATGTAACCGATGCTGAT
	zgc:103559-R	GGATGCGAGAGTTCTTGTTCT
	scp2b-F	GGAGGAGTCAGTCAGGAAGAA
	scp2b-R	CCGTATTACACAGAGGCTGAAC
	fi32c03-F	CCCACTGCTATTCACCAAACT
	fi32c03-R	CGACACAAGGATCAGAGAACAC
	zgc:85777-F	TGAGCTGCAGATGGAGATTG
	zgc:85777-R	GCCAACTTCGTCACATCATTTC
	zp3-F	GCCAATGGATGGTTTGCTAATG
	zp3-R	CTTCCCACTGAATGCCTCTAAA
	zp3.2-F	TGGTGTTGAAGGCCAACTTA
	zp3.2-R	GATTCCTTTGCGCGTCTTTG
	zgc:152936-F	GCTATGTACCAGCCACCAAT
	zgc:152936-R	ACGCCTGCAGCAGTATTT
	aldh9a1b-F	CAAGGACAGGTGTGCAGTAA
	aldh9a1b-R	GAGGGTCTCCAATGCTGATAG
	neu3.2-F	CTGCCATCCAGCCGATAAA
	neu3.2-R	GACCCTCATGCAGAATCACA
	clpb-F	TCGATGATGTGCAGAAGAGTG
	clpb-R	AACTCGTCCCGTCTGAAATG
	gchfr-F	CCTGTGAATGCAGACAGGTAA
	gchfr-R	GCACACGTTTGGAAATTCTCTT
	zgc:101569-F	GGCTCTTGAACTCGCAGAA
	zgc:101569-R	GTTGAAGGAGGTGGAGTCAAA
Tethering	actb2-F	ATCAGGGTGTCATGGTTGGT
	actb2-R	CACGCAGCTCGTTGTAGAAG
	sfGFP_F	GACAACCCTGACATACGGAG
	sfGFP_R	TTCCGTCATCCTTGAAGCTG
