# Supplementary figures and images for: eIF4E1b is a non-canonical eIF4E protecting maternal dormant mRNAs
Source: EMBO Rep. 2023 Dec 14;25(1):23. doi: 10.1038/s44319-023-00006-4 (PMC10883267; doi:10.1038/s44319-023-00006-4)

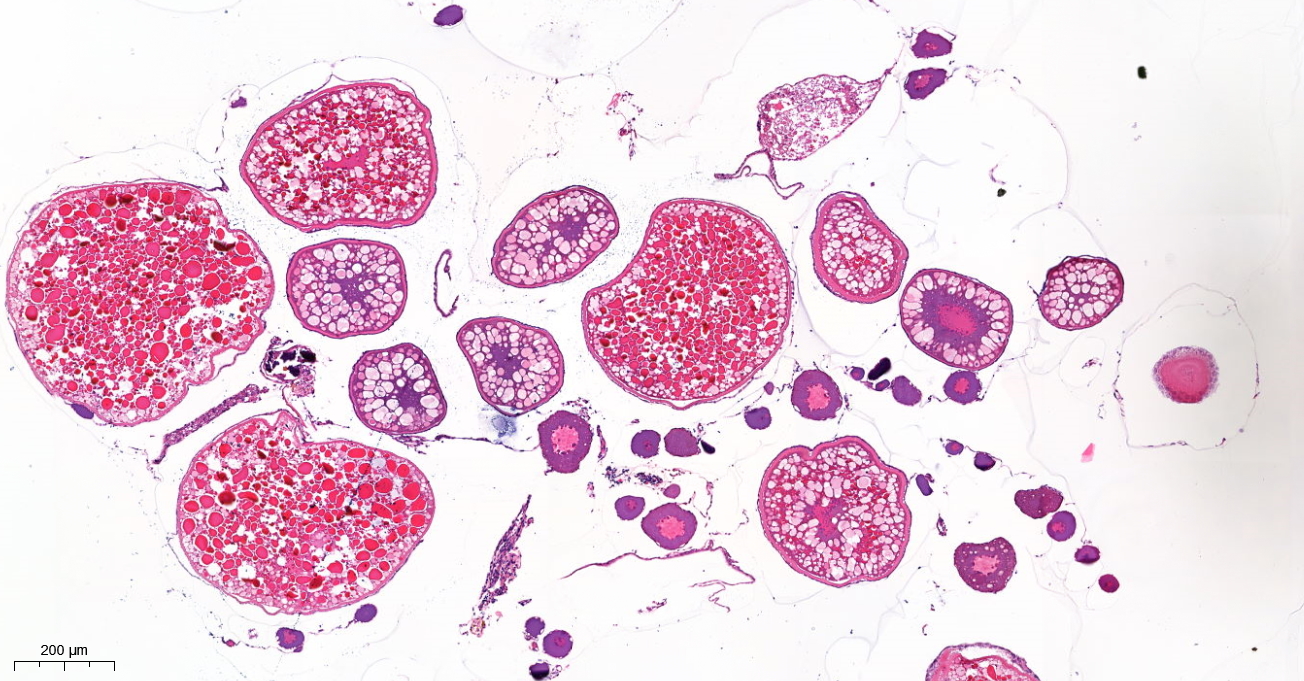

Supplement: Supplementary file 10 — Source Data Fig. 1 [file 44319_2023_6_MOESM10_ESM.zip › Figure 1/1G/Left_WT.jpg]

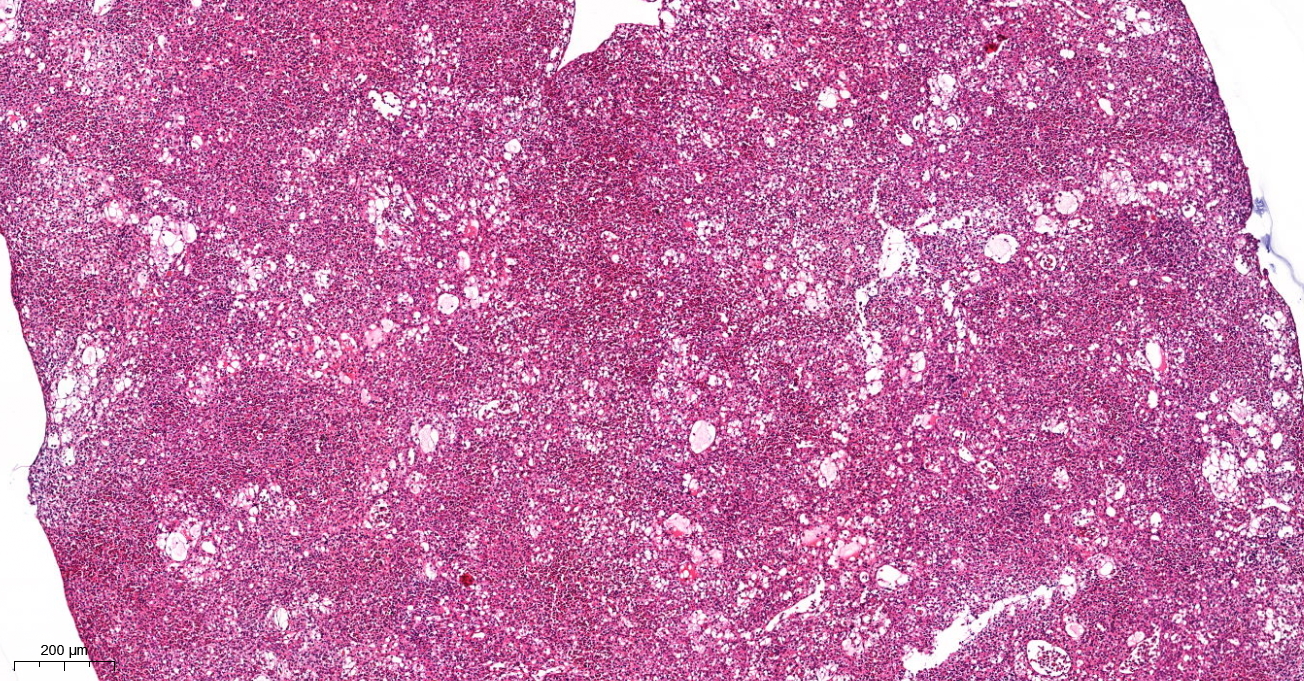

Supplement: Supplementary file 10 — Source Data Fig. 1 [file 44319_2023_6_MOESM10_ESM.zip › Figure 1/1G/Right_eif4e1b-hom.jpg]

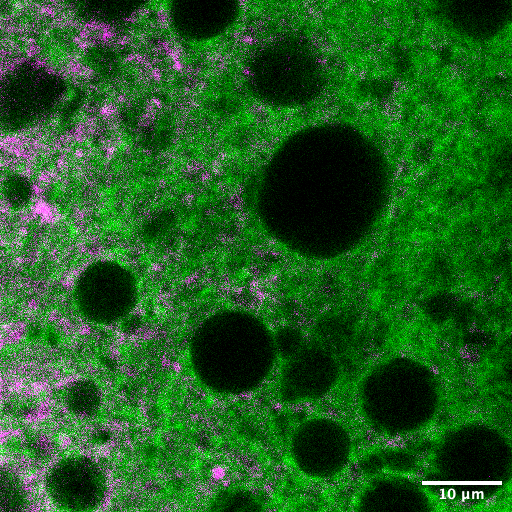

Supplement: Supplementary file 11 — Source Data Fig. 2 [file 44319_2023_6_MOESM11_ESM.zip › Figure 2/2F/2F_5.tif]

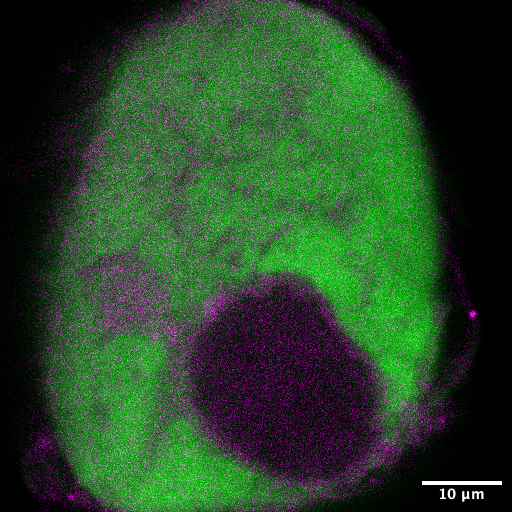

Supplement: Supplementary file 11 — Source Data Fig. 2 [file 44319_2023_6_MOESM11_ESM.zip › Figure 2/2F/2F_4.tif]

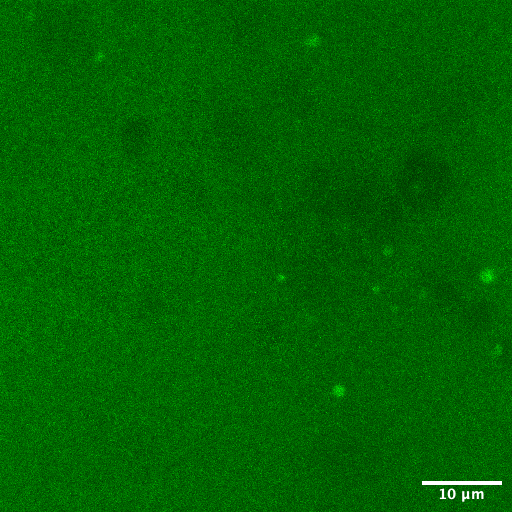

Supplement: Supplementary file 11 — Source Data Fig. 2 [file 44319_2023_6_MOESM11_ESM.zip › Figure 2/2F/2F_6.tif]

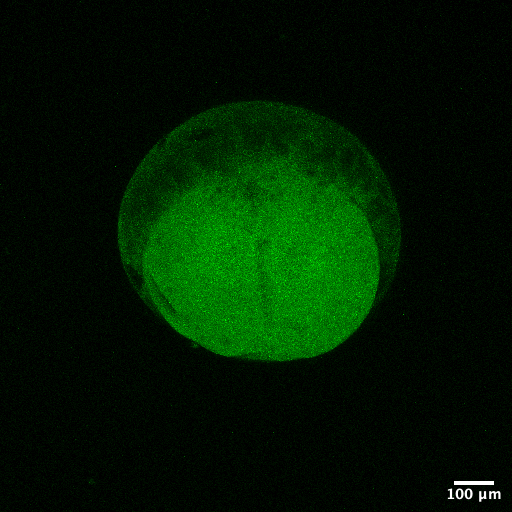

Supplement: Supplementary file 11 — Source Data Fig. 2 [file 44319_2023_6_MOESM11_ESM.zip › Figure 2/2F/2F_3.tif]

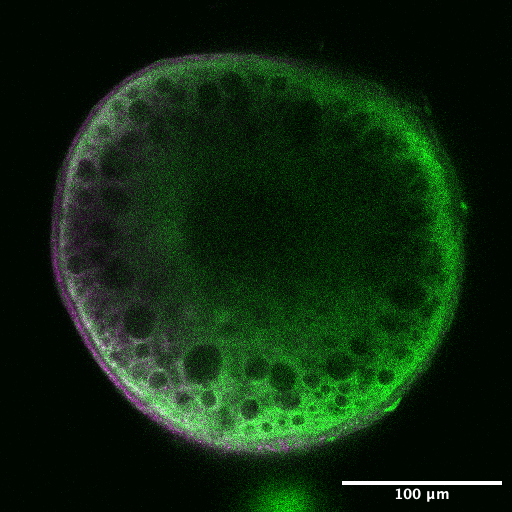

Supplement: Supplementary file 11 — Source Data Fig. 2 [file 44319_2023_6_MOESM11_ESM.zip › Figure 2/2F/2F_2.tif]

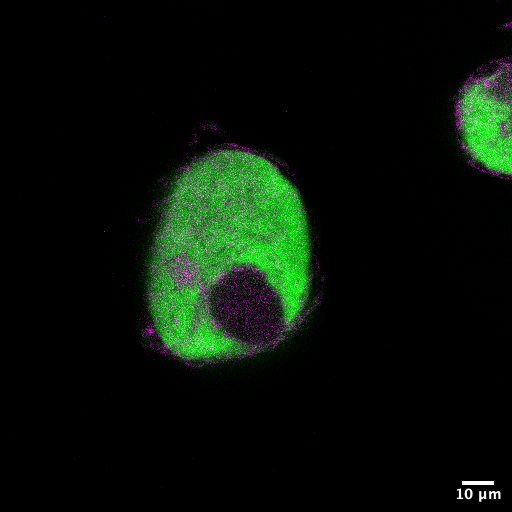

Supplement: Supplementary file 11 — Source Data Fig. 2 [file 44319_2023_6_MOESM11_ESM.zip › Figure 2/2F/2F_1.tif]

|           | Ni <sup>2+</sup> -Pulldown |   |   |   |   |   |   | Lysate |   |   |   |
|-----------|----------------------------|---|---|---|---|---|---|--------|---|---|---|
| His-eIF4E | +                          | + | + | + | - | - | - | +      | - | - | - |
| eIF4G     | -                          | + | - | - | + | - | - | -      | + | - | - |
| eIF4EBP1  | -                          | - | + | - | - | + | - | -      | - | + | - |
| eIF4ENIF1 | -                          | - | - | + | - | - | + | -      | - | - | + |

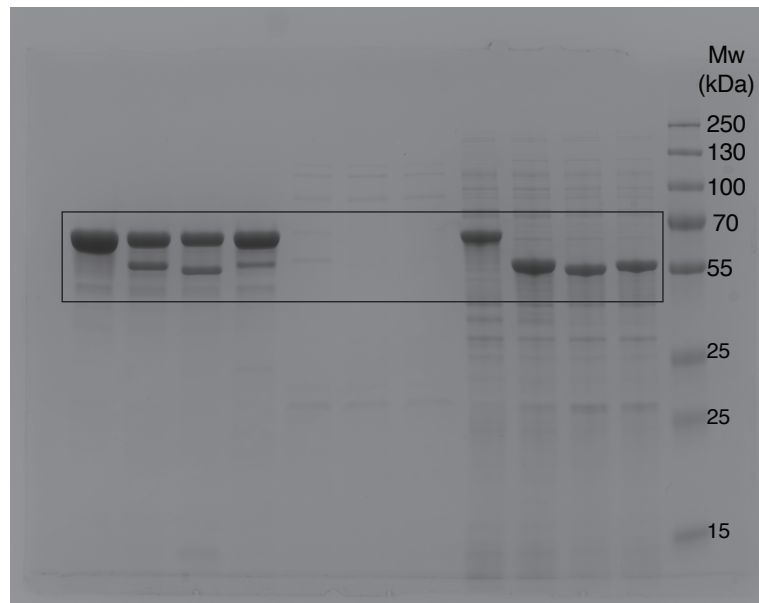

Supplement: Supplementary file 11 — Source Data Fig. 2 [file 44319_2023_6_MOESM11_ESM.zip › Figure 2/2D/fig2D_example-gel.pdf]

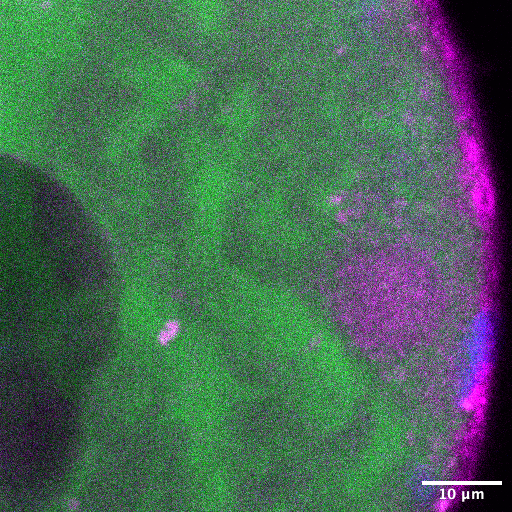

Supplement: Supplementary file 11 — Source Data Fig. 2 [file 44319_2023_6_MOESM11_ESM.zip › Figure 2/2E/2E_4.tif]

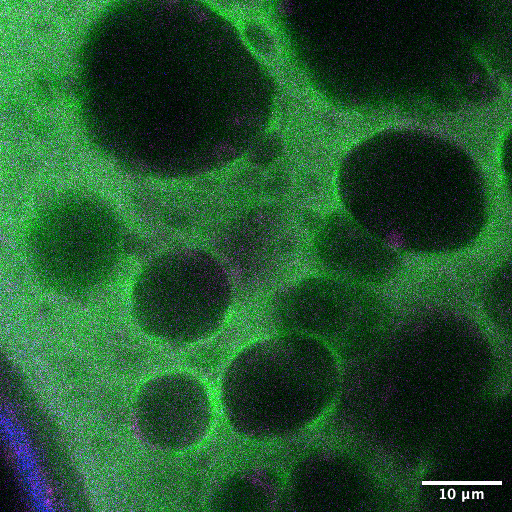

Supplement: Supplementary file 11 — Source Data Fig. 2 [file 44319_2023_6_MOESM11_ESM.zip › Figure 2/2E/2E_5.tif]

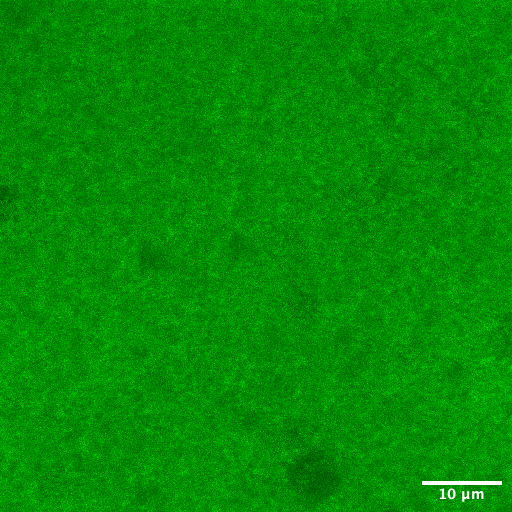

Supplement: Supplementary file 11 — Source Data Fig. 2 [file 44319_2023_6_MOESM11_ESM.zip › Figure 2/2E/2E_6.tif]

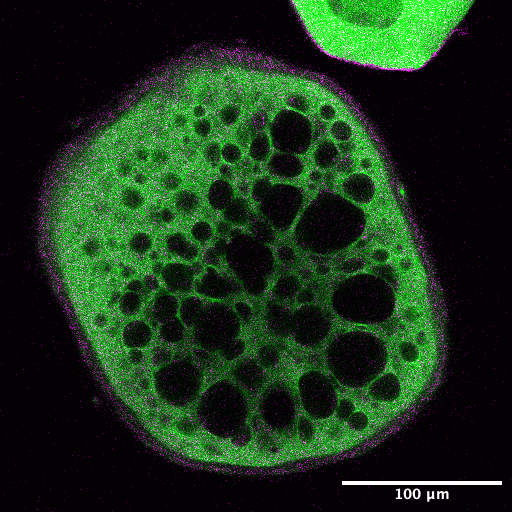

Supplement: Supplementary file 11 — Source Data Fig. 2 [file 44319_2023_6_MOESM11_ESM.zip › Figure 2/2E/2E_2.tif]

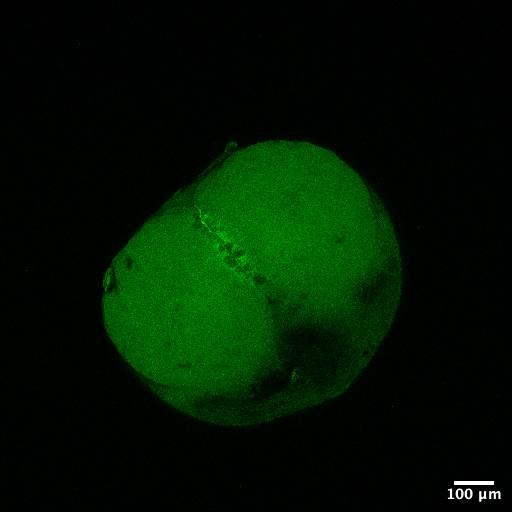

Supplement: Supplementary file 11 — Source Data Fig. 2 [file 44319_2023_6_MOESM11_ESM.zip › Figure 2/2E/2E_3.tif]

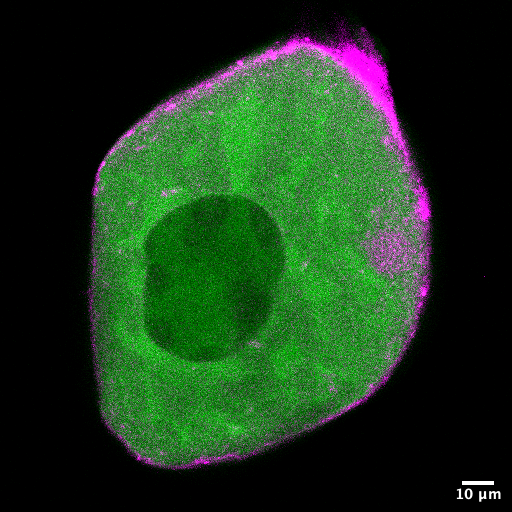

Supplement: Supplementary file 11 — Source Data Fig. 2 [file 44319_2023_6_MOESM11_ESM.zip › Figure 2/2E/2E_1.tif]

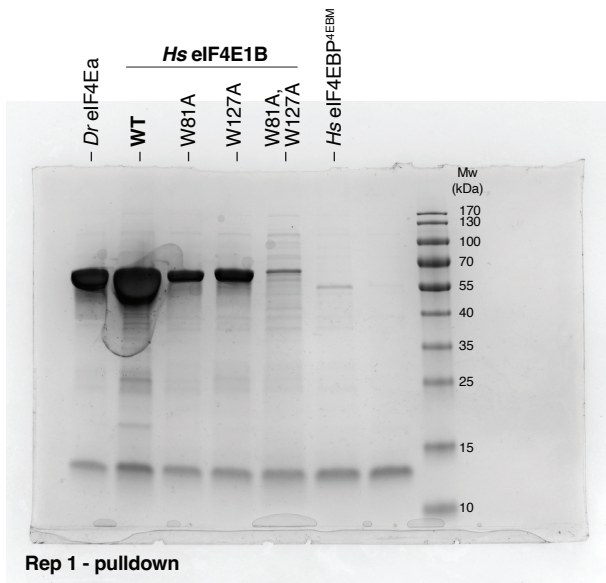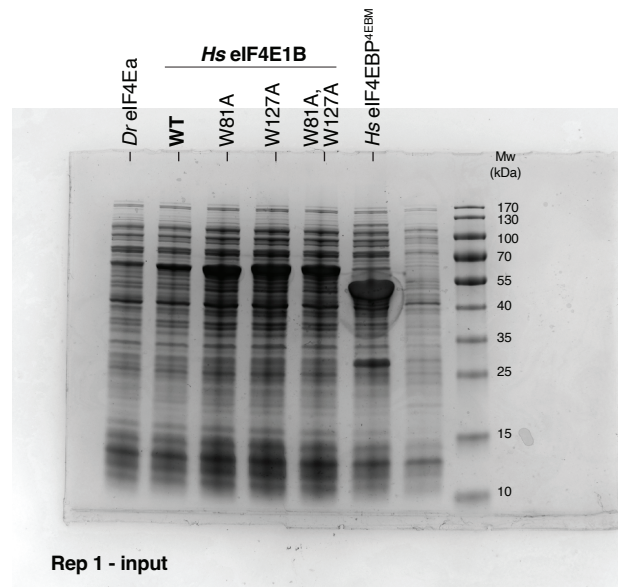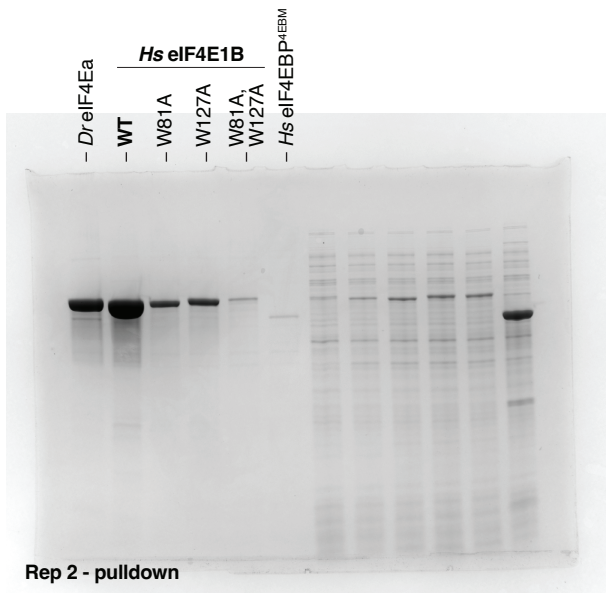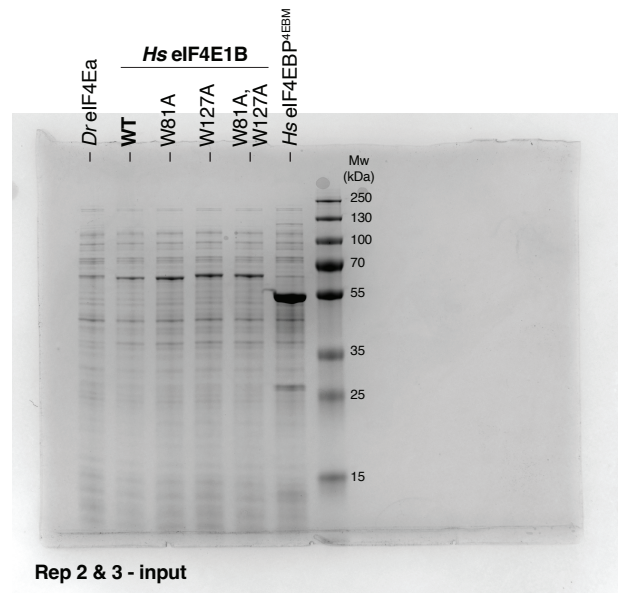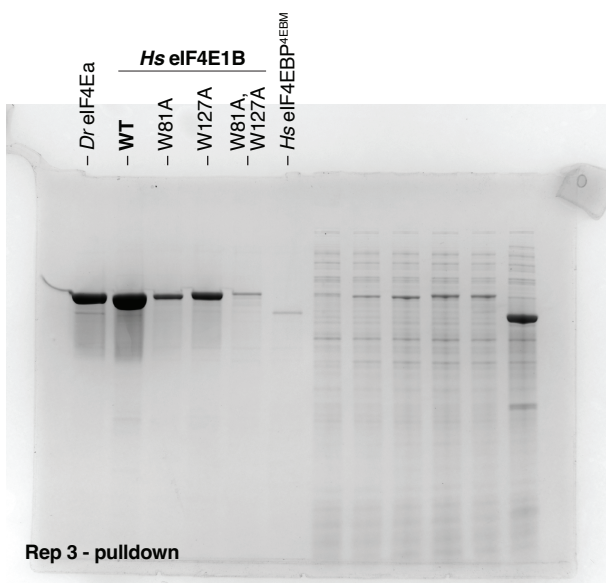

Supplement: Supplementary file 11 — Source Data Fig. 2 [file 44319_2023_6_MOESM11_ESM.zip › Figure 2/2B/fig2B.pdf]

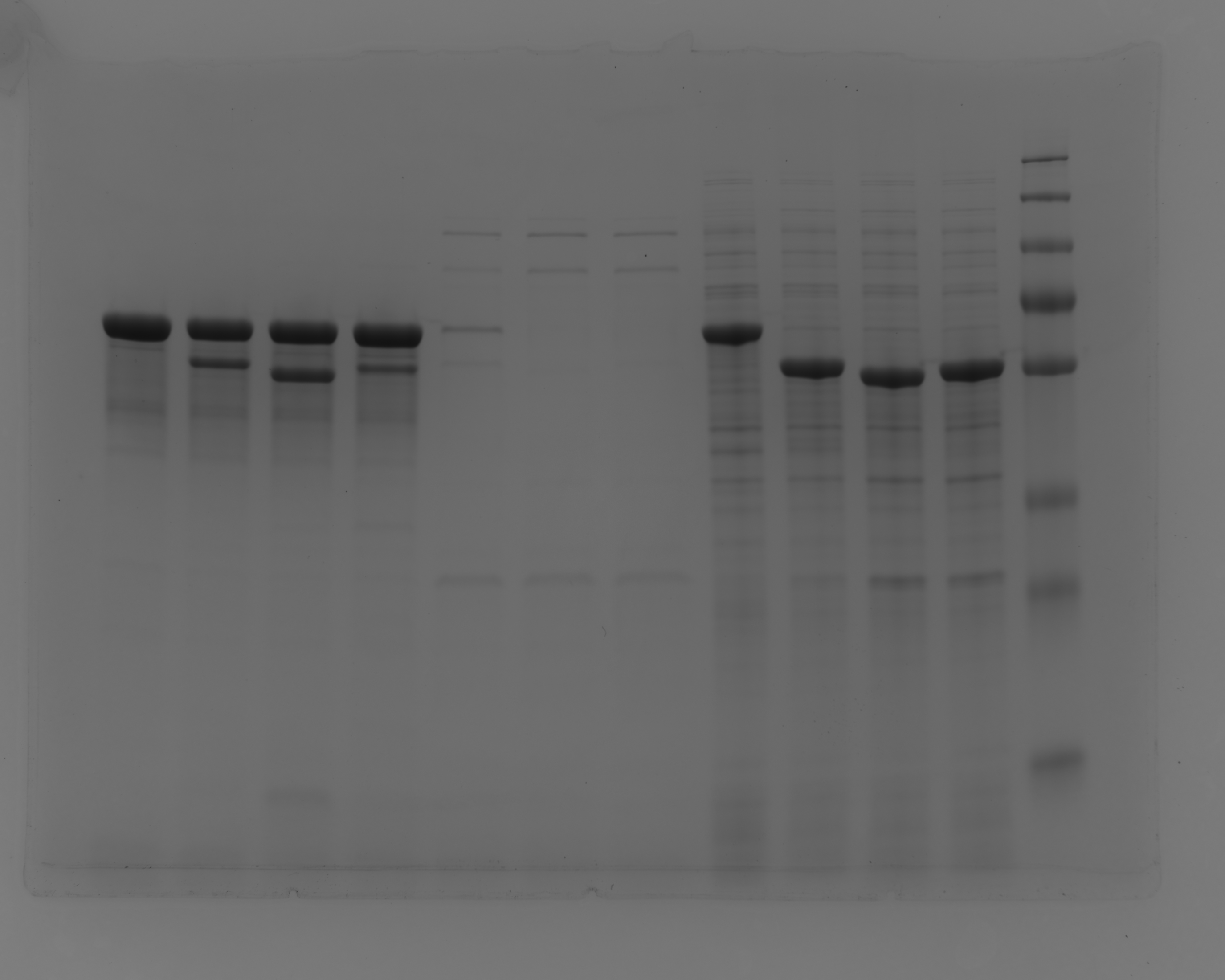

Supplement: Supplementary file 11 — Source Data Fig. 2 [file 44319_2023_6_MOESM11_ESM.zip › Figure 2/2D/Dr eIF4Ea/admin1 2022-08-02 10h55m06s(Coomassie Blue).raw16.tif]

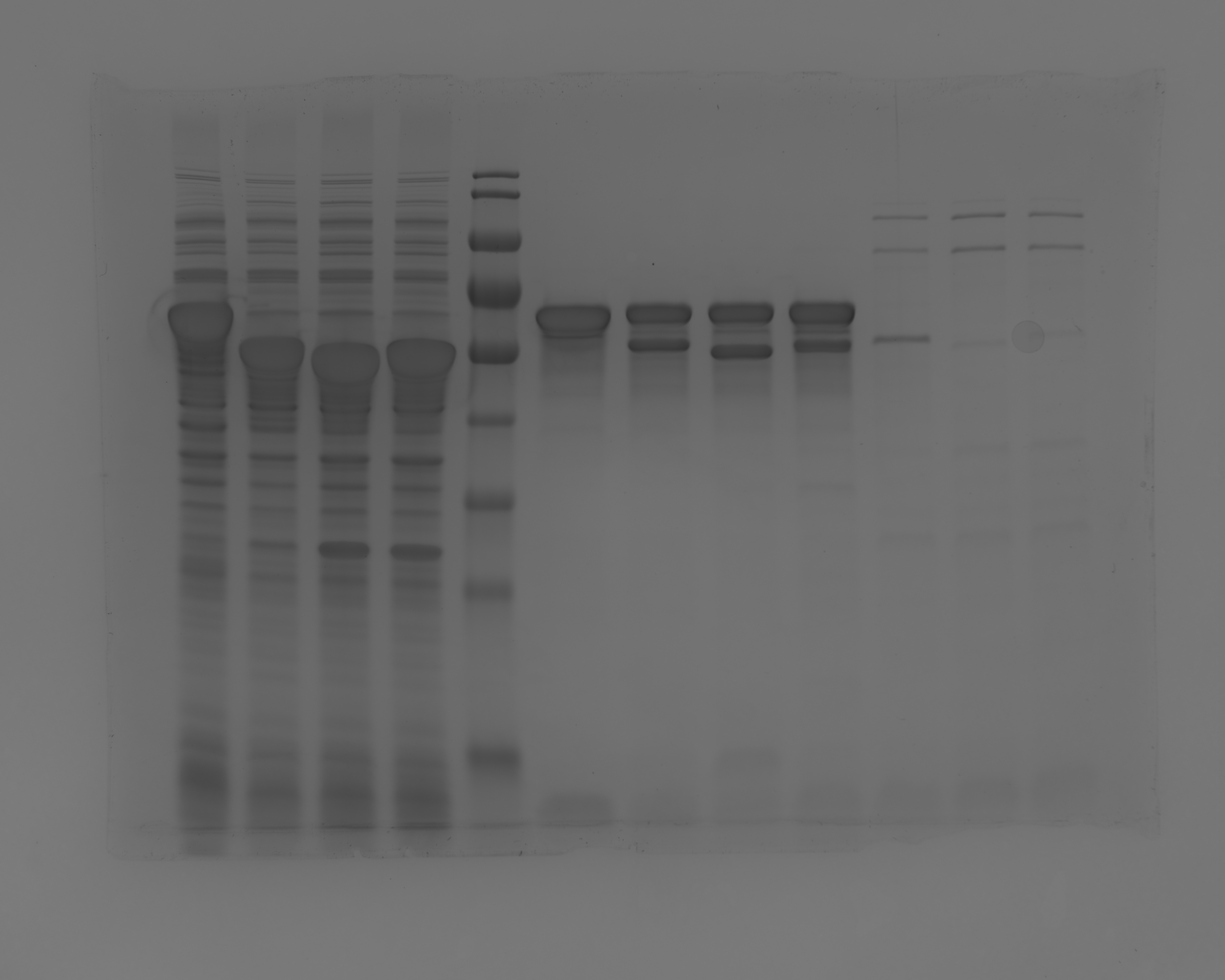

Supplement: Supplementary file 11 — Source Data Fig. 2 [file 44319_2023_6_MOESM11_ESM.zip › Figure 2/2D/Dr eIF4Ea/admin1 2021-08-02 15h42m03s(Coomassie Blue).raw16.tif]

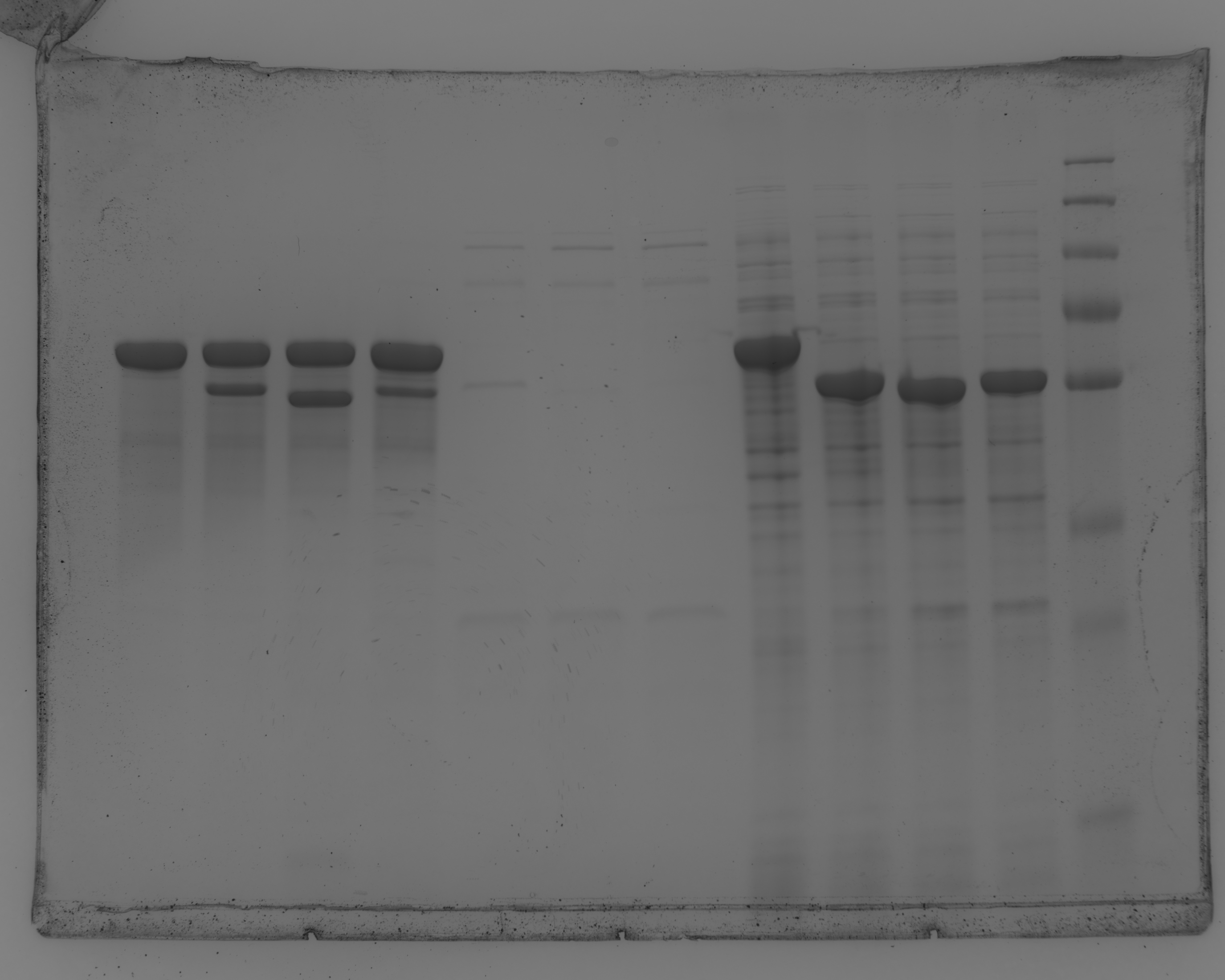

Supplement: Supplementary file 11 — Source Data Fig. 2 [file 44319_2023_6_MOESM11_ESM.zip › Figure 2/2D/Dr eIF4Ea/admin1 2022-07-27 10h05m26s(Coomassie Blue).raw16.tif]

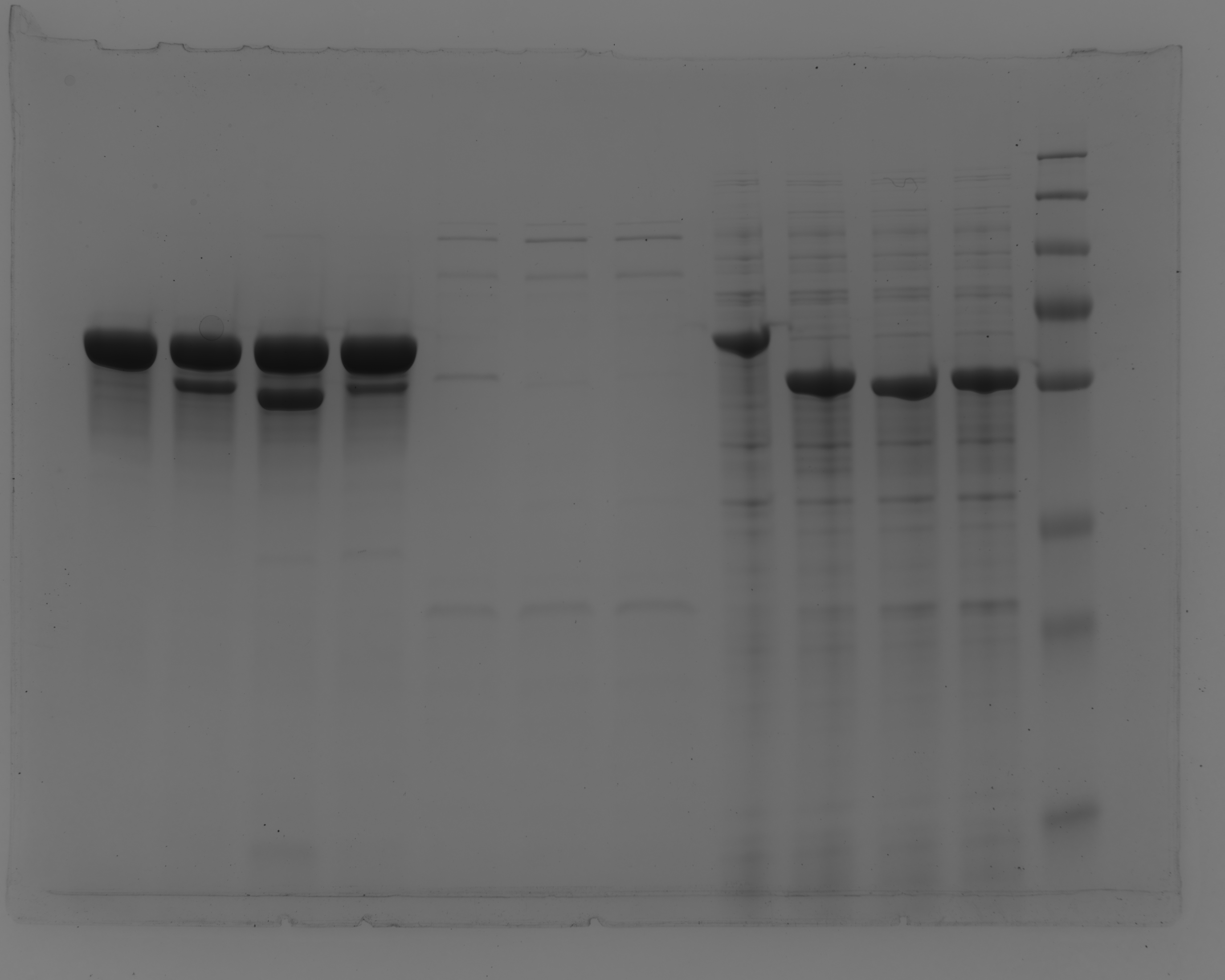

Supplement: Supplementary file 11 — Source Data Fig. 2 [file 44319_2023_6_MOESM11_ESM.zip › Figure 2/2D/Dr eIF4E1c/admin1 2022-07-27 10h07m48s(Coomassie Blue).raw16.tif]

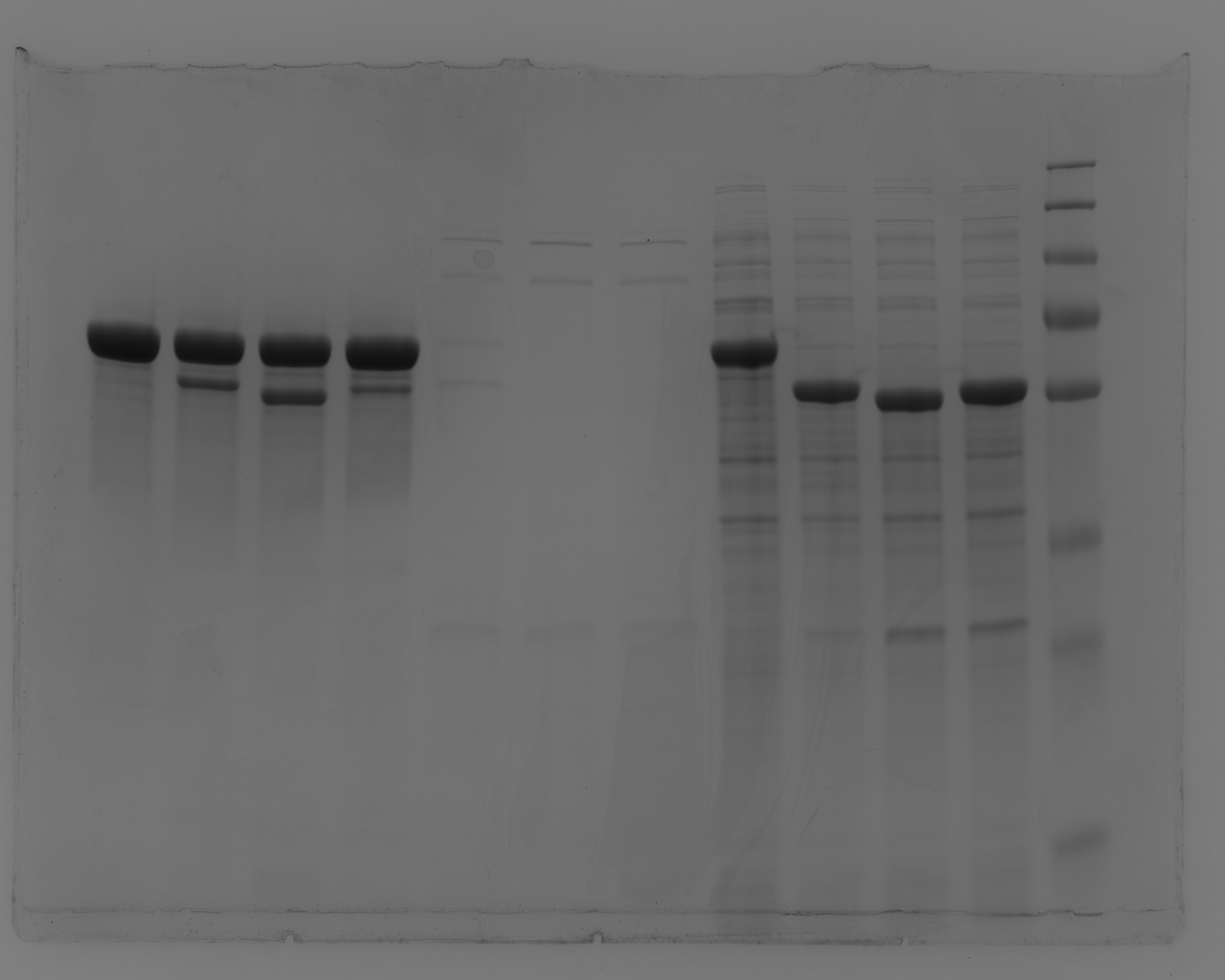

Supplement: Supplementary file 11 — Source Data Fig. 2 [file 44319_2023_6_MOESM11_ESM.zip › Figure 2/2D/Dr eIF4E1c/admin1 2022-06-27 12h36m24s(Coomassie Blue).raw16.tif]

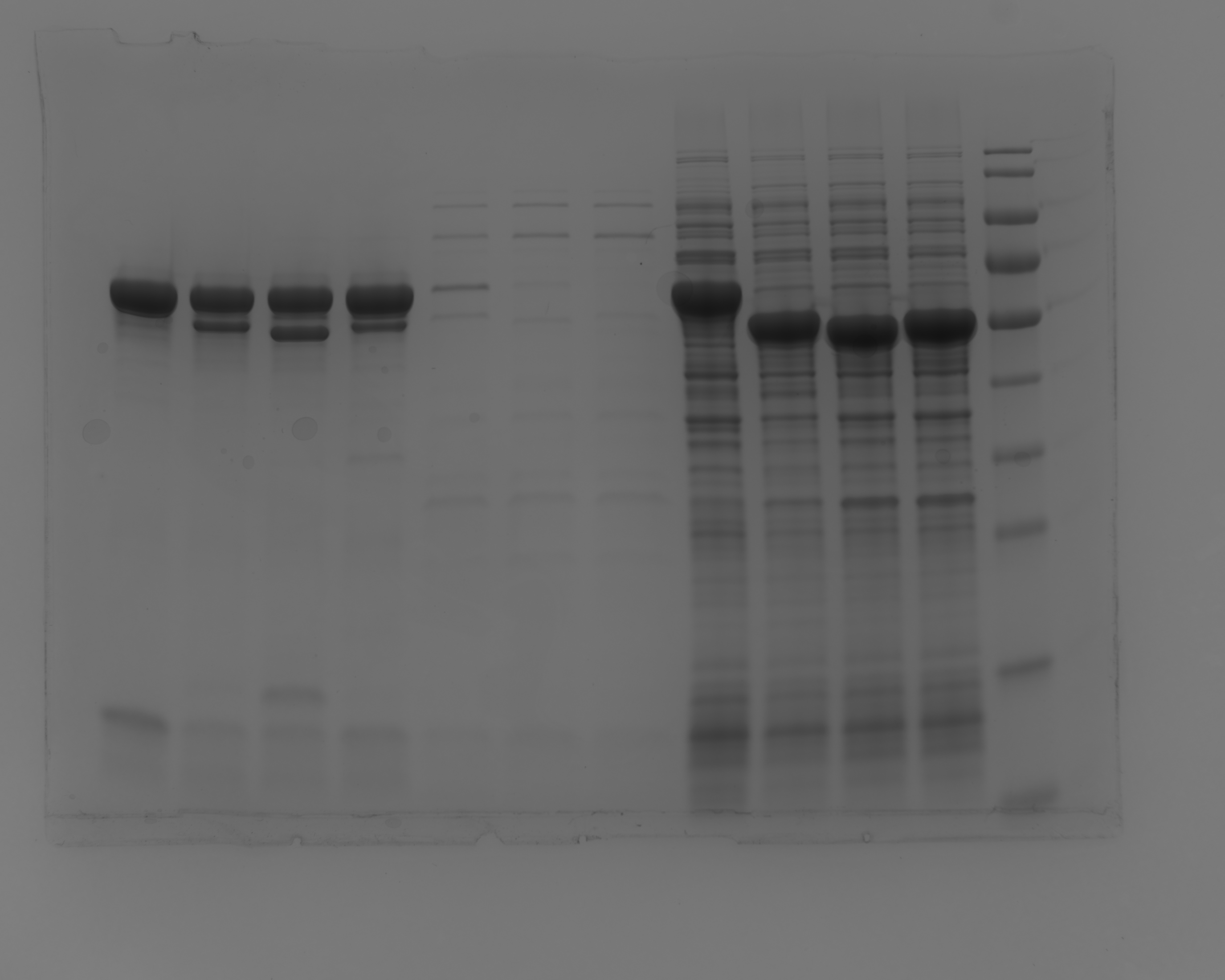

Supplement: Supplementary file 11 — Source Data Fig. 2 [file 44319_2023_6_MOESM11_ESM.zip › Figure 2/2D/Dr eIF4E1c/admin1 2021-12-09 12h54m01s(Coomassie Blue).raw16.tif]

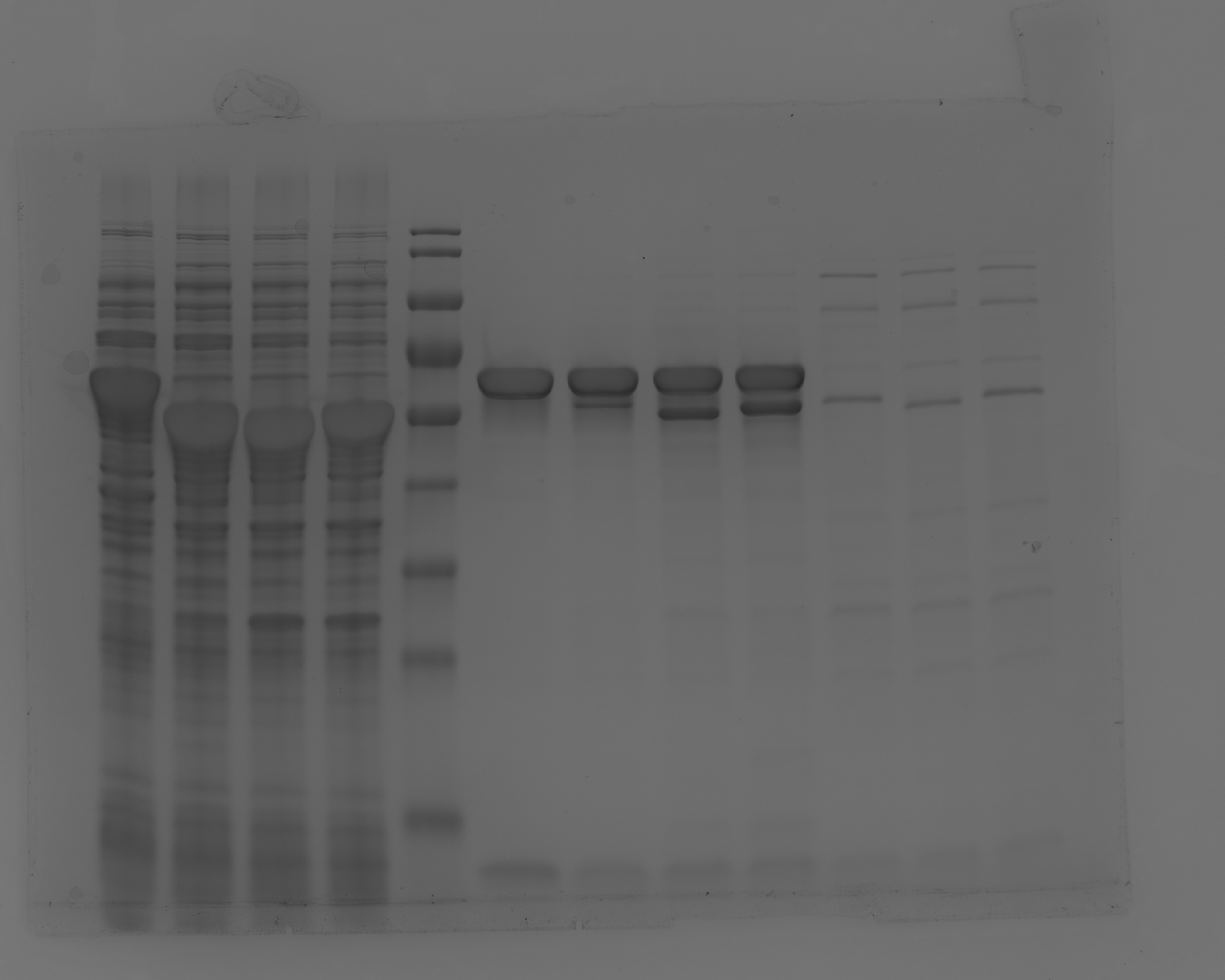

Supplement: Supplementary file 11 — Source Data Fig. 2 [file 44319_2023_6_MOESM11_ESM.zip › Figure 2/2D/Mm eIF4E1B/admin1 2021-09-17 08h58m42s(Coomassie Blue).raw16.tif]

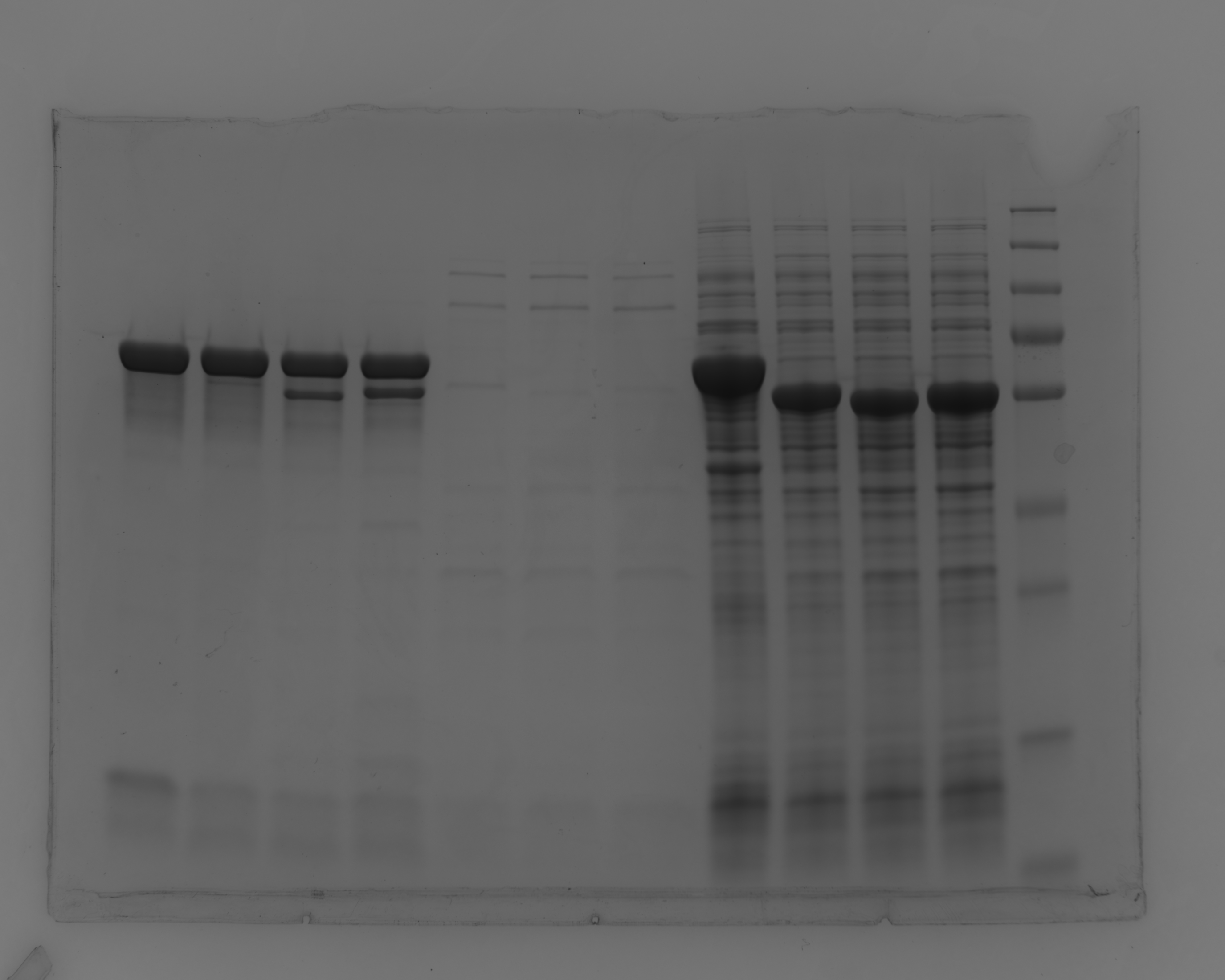

Supplement: Supplementary file 11 — Source Data Fig. 2 [file 44319_2023_6_MOESM11_ESM.zip › Figure 2/2D/Mm eIF4E1B/admin1 2022-02-10 10h14m59s(Coomassie Blue).raw16.tif]

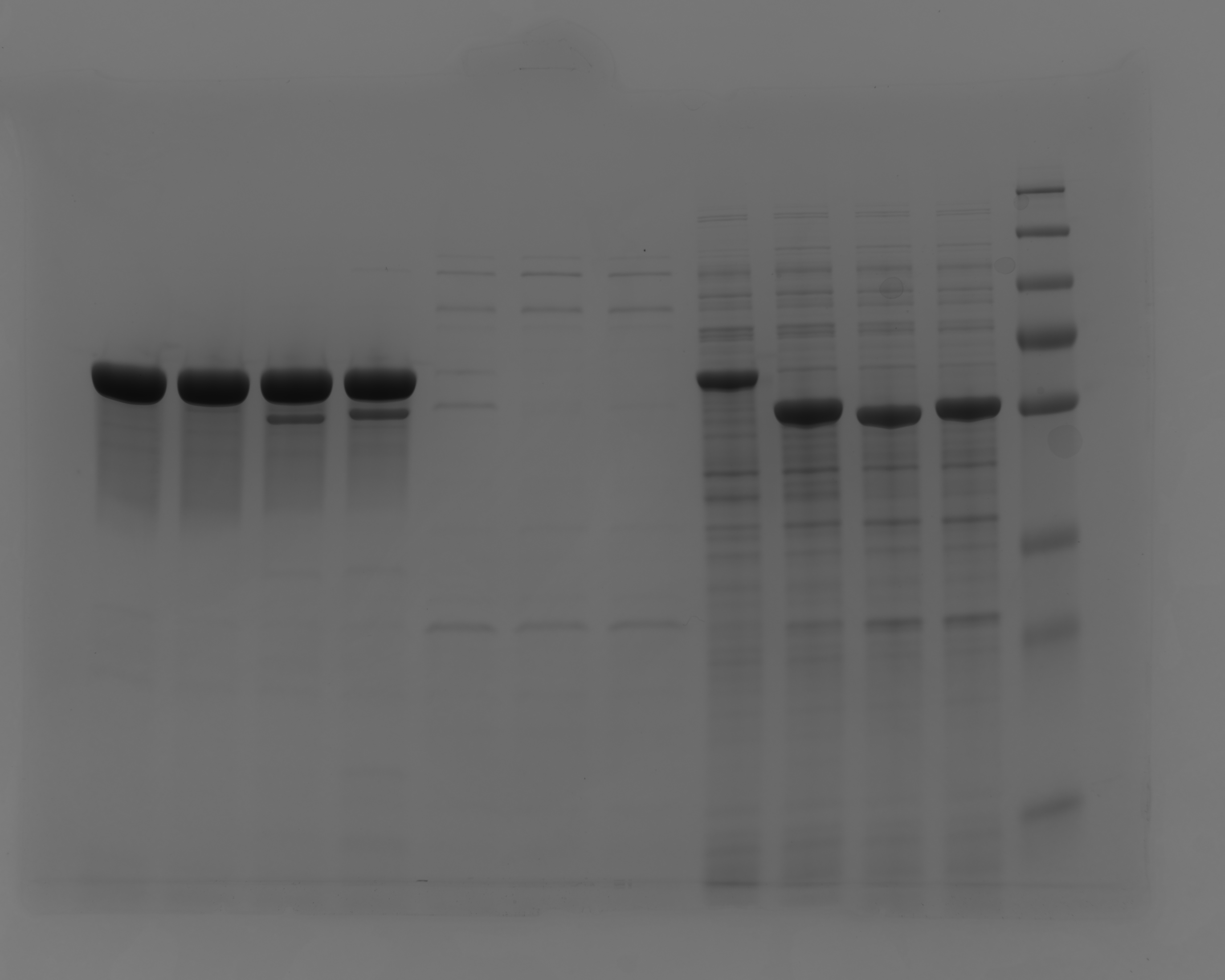

Supplement: Supplementary file 11 — Source Data Fig. 2 [file 44319_2023_6_MOESM11_ESM.zip › Figure 2/2D/Mm eIF4E1B/admin1 2022-06-13 08h32m45s(Coomassie Blue).raw16.tif]

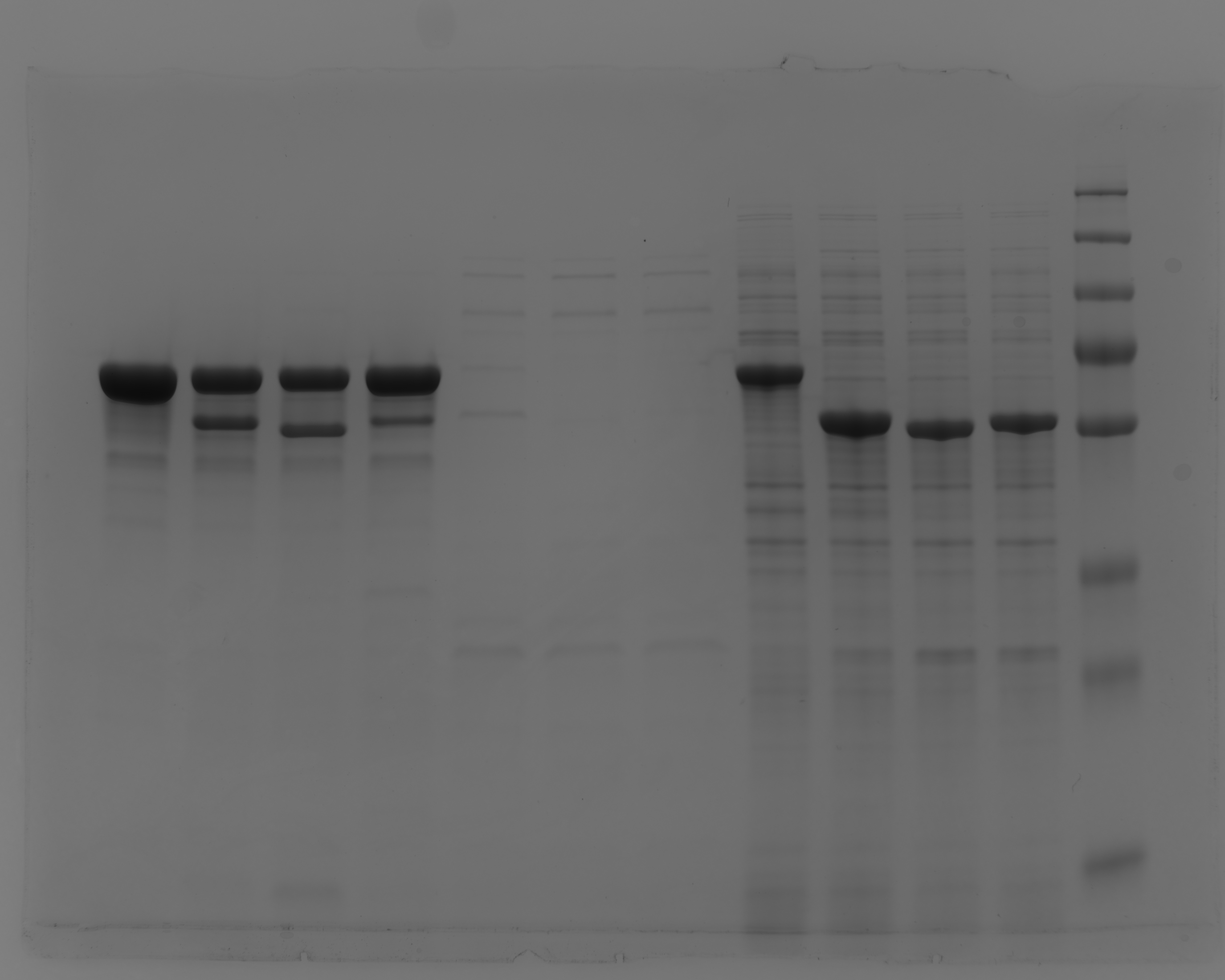

Supplement: Supplementary file 11 — Source Data Fig. 2 [file 44319_2023_6_MOESM11_ESM.zip › Figure 2/2D/Mm eIF4E/admin1 2022-05-16 08h29m45s(Coomassie Blue).raw16.tif]

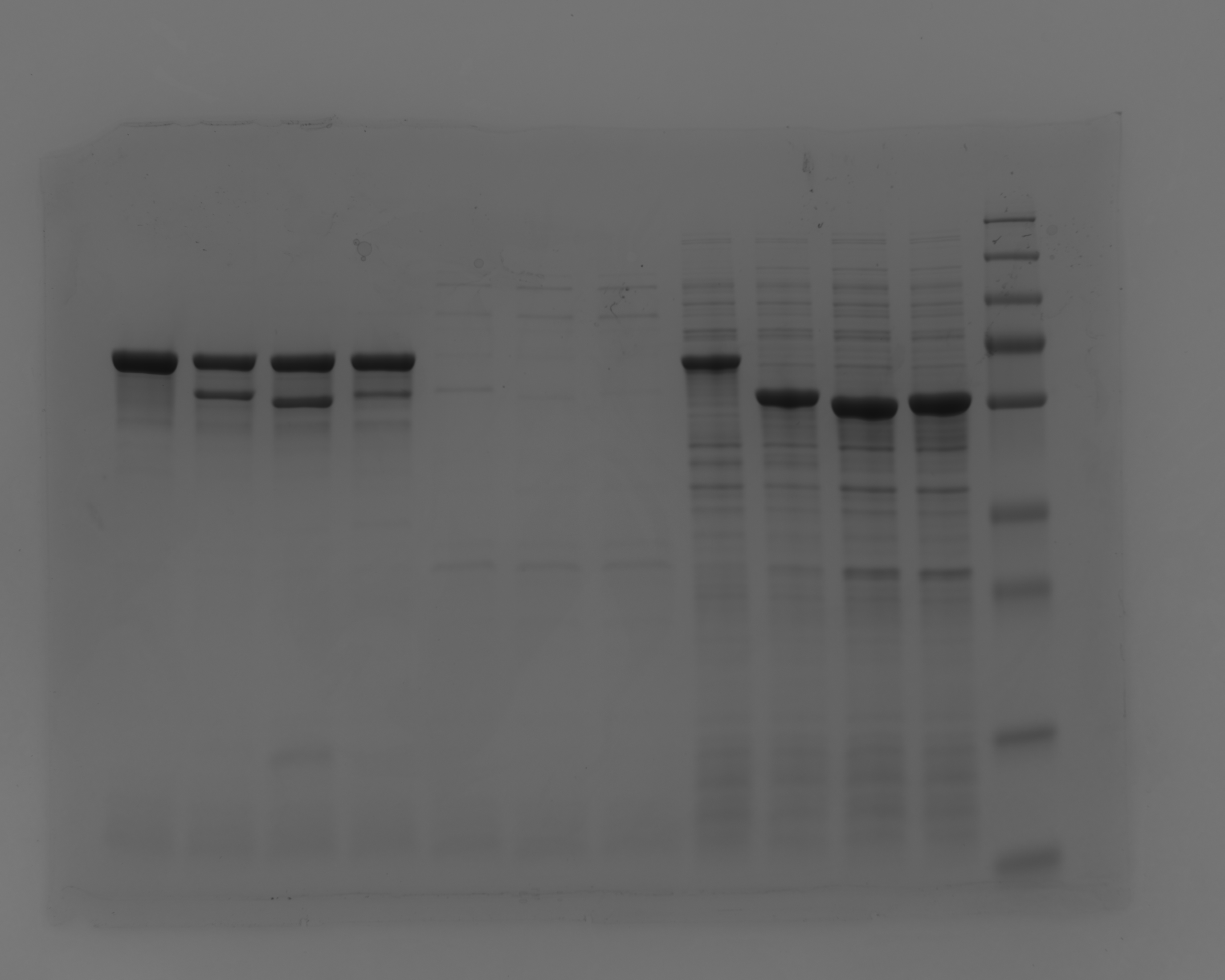

Supplement: Supplementary file 11 — Source Data Fig. 2 [file 44319_2023_6_MOESM11_ESM.zip › Figure 2/2D/Mm eIF4E/admin1 2022-05-08 13h48m08s(Coomassie Blue).raw16.tif]

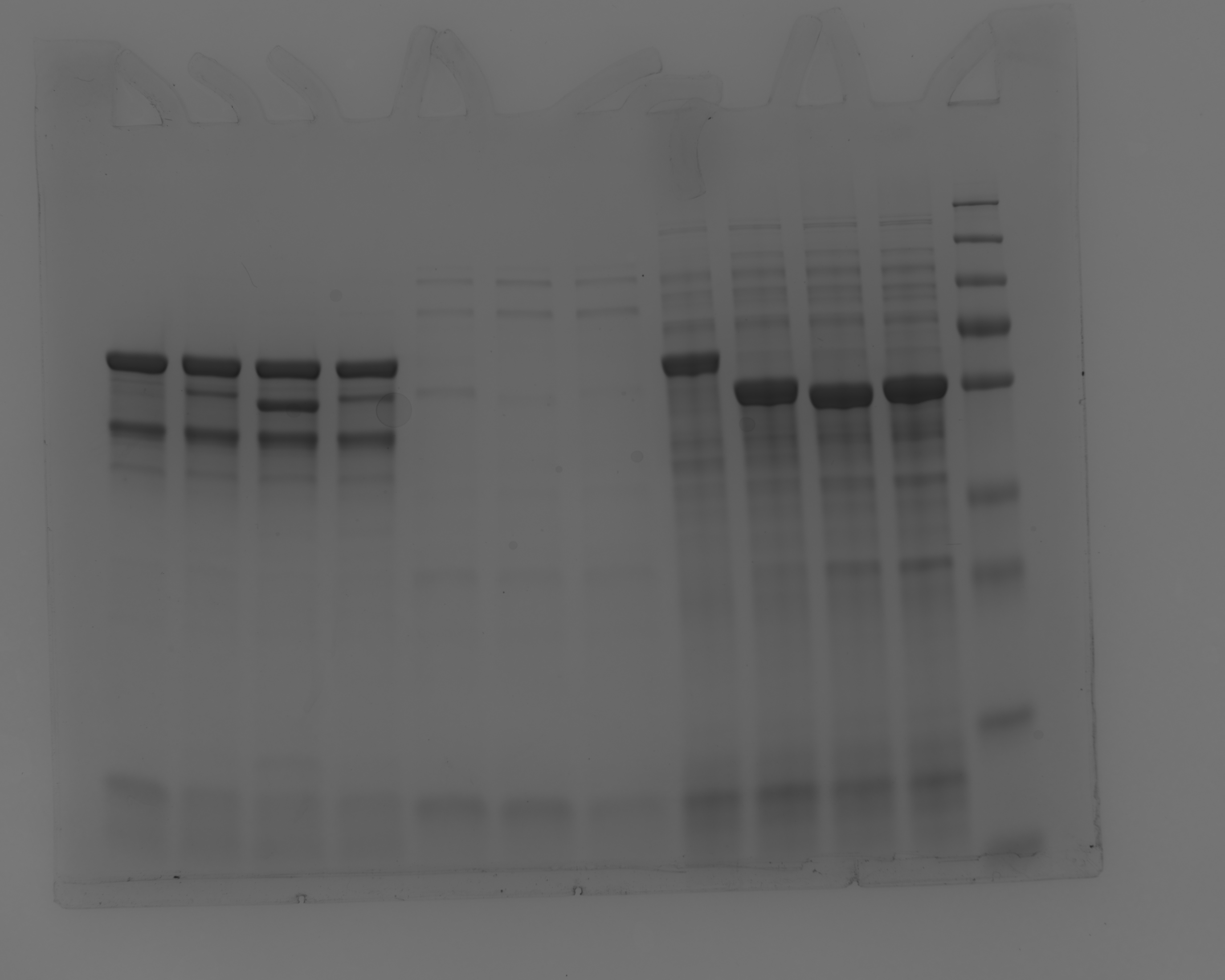

Supplement: Supplementary file 11 — Source Data Fig. 2 [file 44319_2023_6_MOESM11_ESM.zip › Figure 2/2D/Mm eIF4E/admin1 2022-04-28 14h25m07s(Coomassie Blue).raw16.tif]

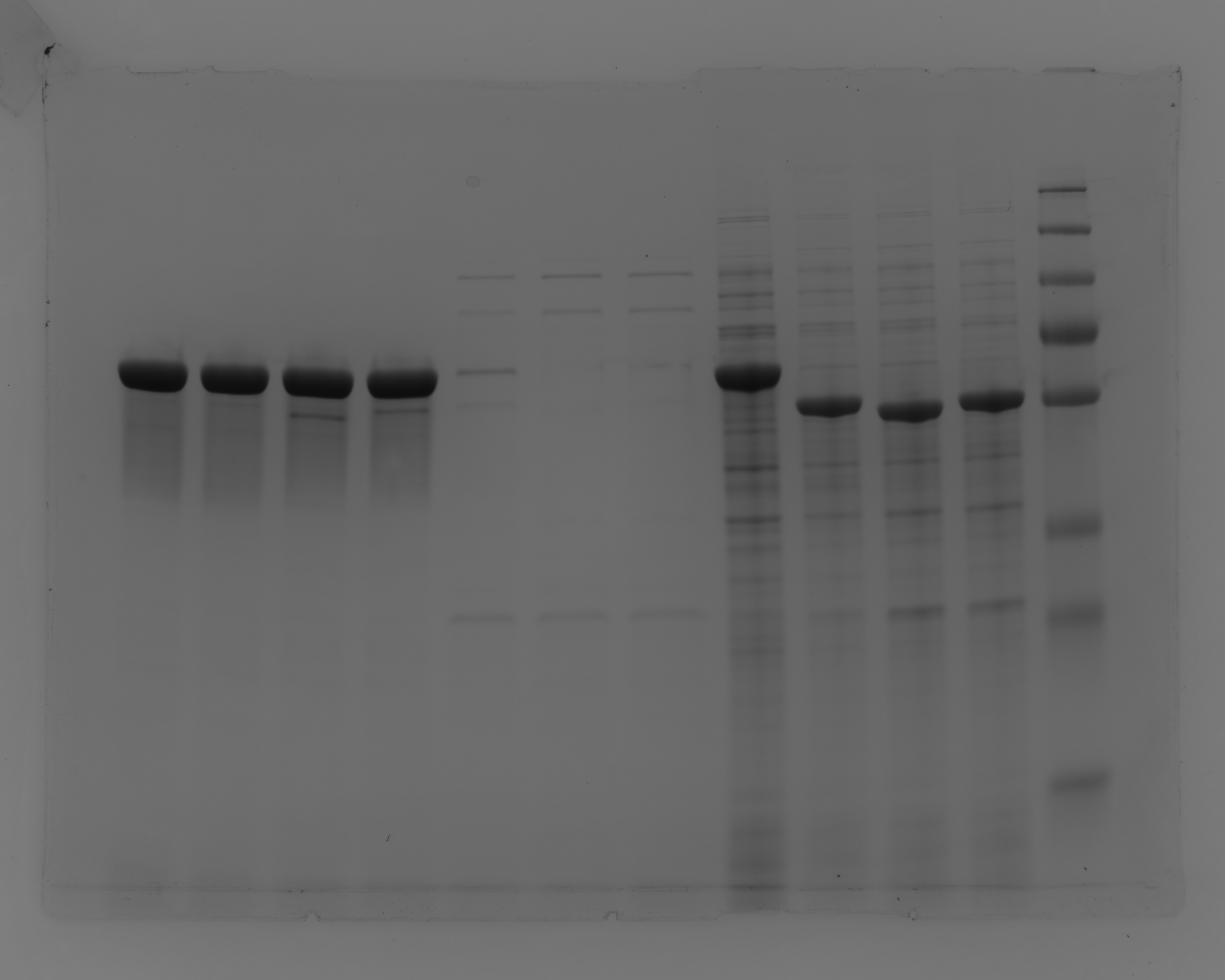

Supplement: Supplementary file 11 — Source Data Fig. 2 [file 44319_2023_6_MOESM11_ESM.zip › Figure 2/2D/Dr eIF4E2/admin1 2022-08-02 10h56m45s(Coomassie Blue).raw16.tif]

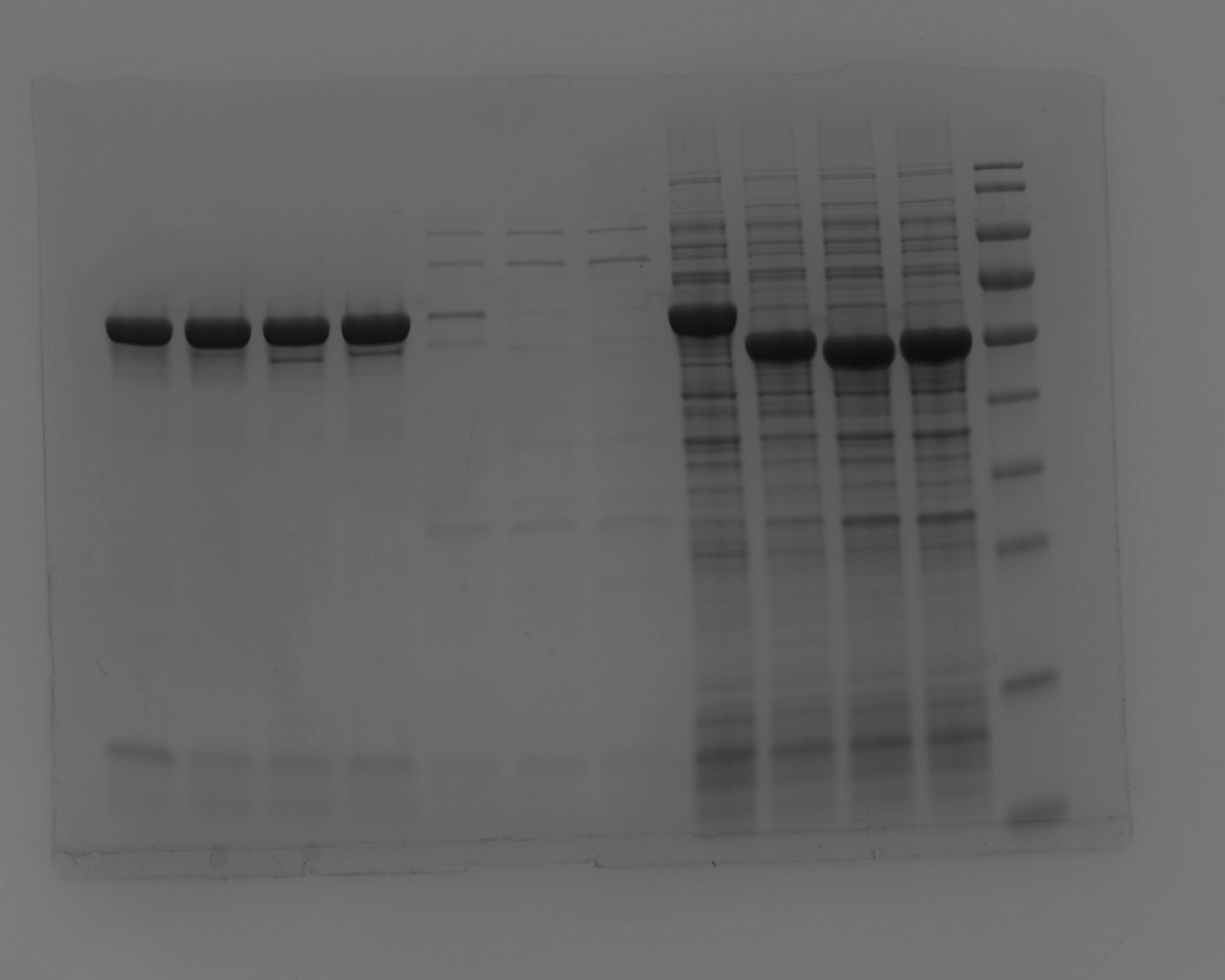

Supplement: Supplementary file 11 — Source Data Fig. 2 [file 44319_2023_6_MOESM11_ESM.zip › Figure 2/2D/Dr eIF4E2/admin1 2021-12-09 12h55m33s(Coomassie Blue).raw16.tif]

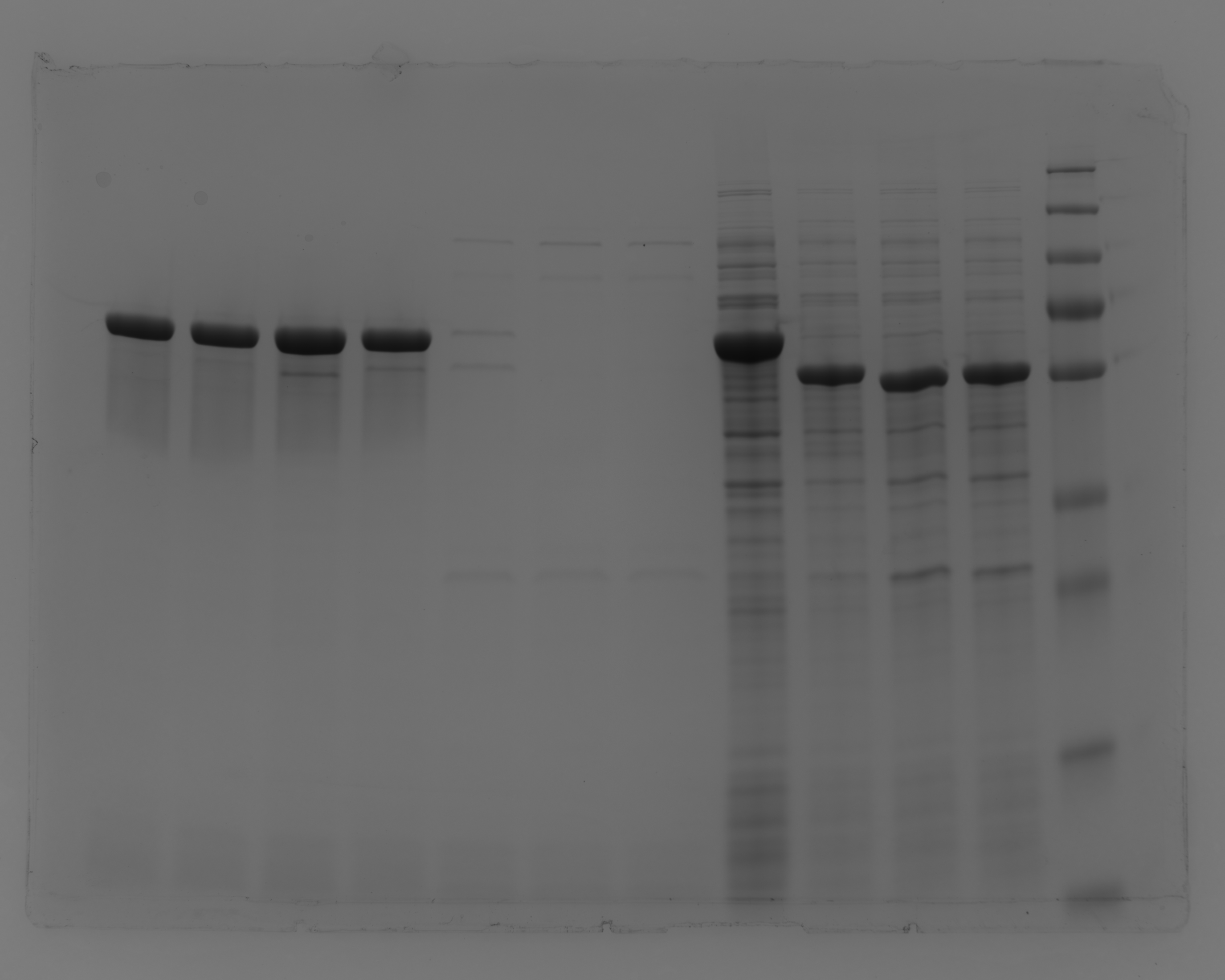

Supplement: Supplementary file 11 — Source Data Fig. 2 [file 44319_2023_6_MOESM11_ESM.zip › Figure 2/2D/Dr eIF4E2/admin1 2022-07-05 10h32m35s(Coomassie Blue).raw16.tif]

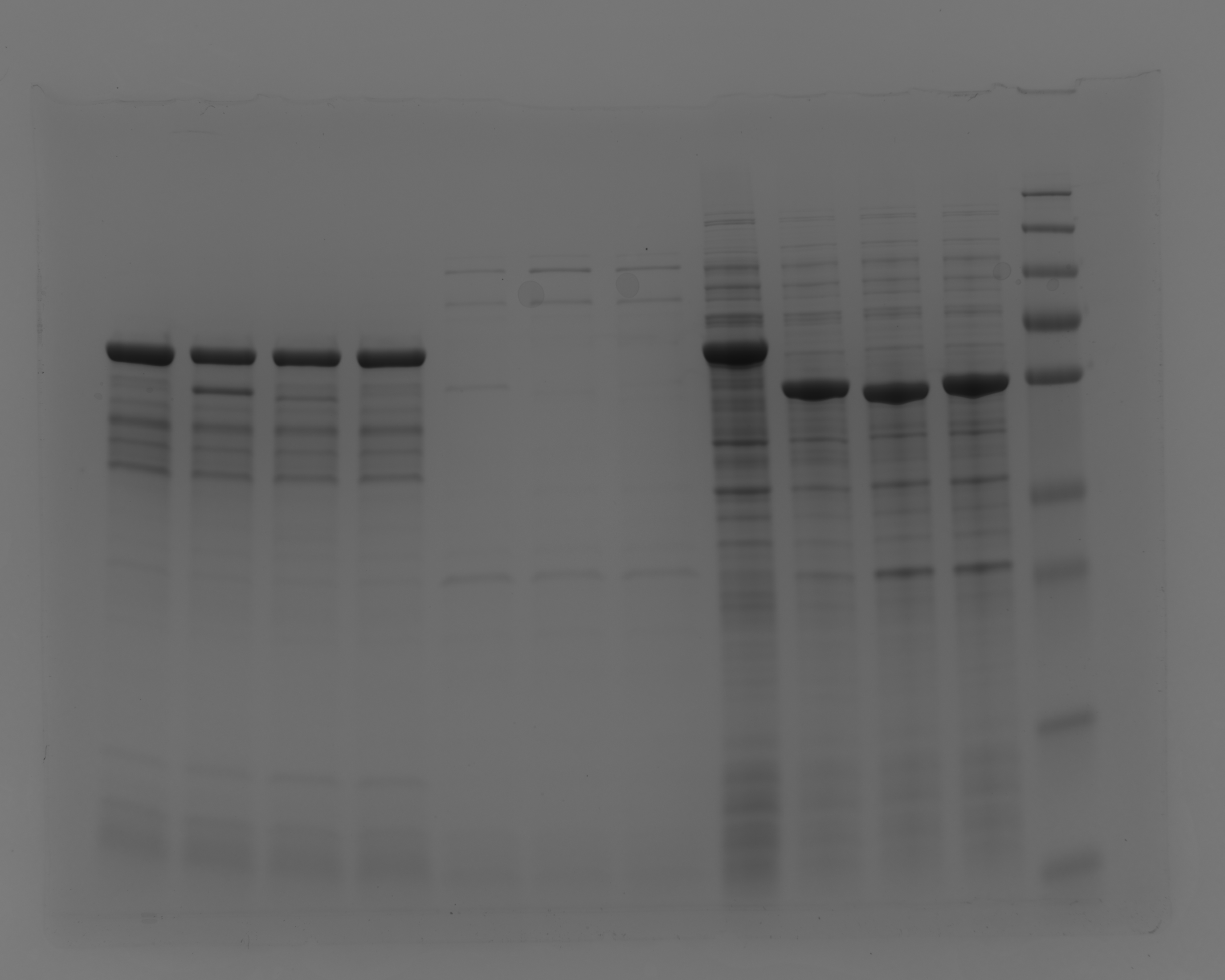

Supplement: Supplementary file 11 — Source Data Fig. 2 [file 44319_2023_6_MOESM11_ESM.zip › Figure 2/2D/Dr eIF4E3/admin1 2022-07-04 10h29m03s(Coomassie Blue).raw16.tif]

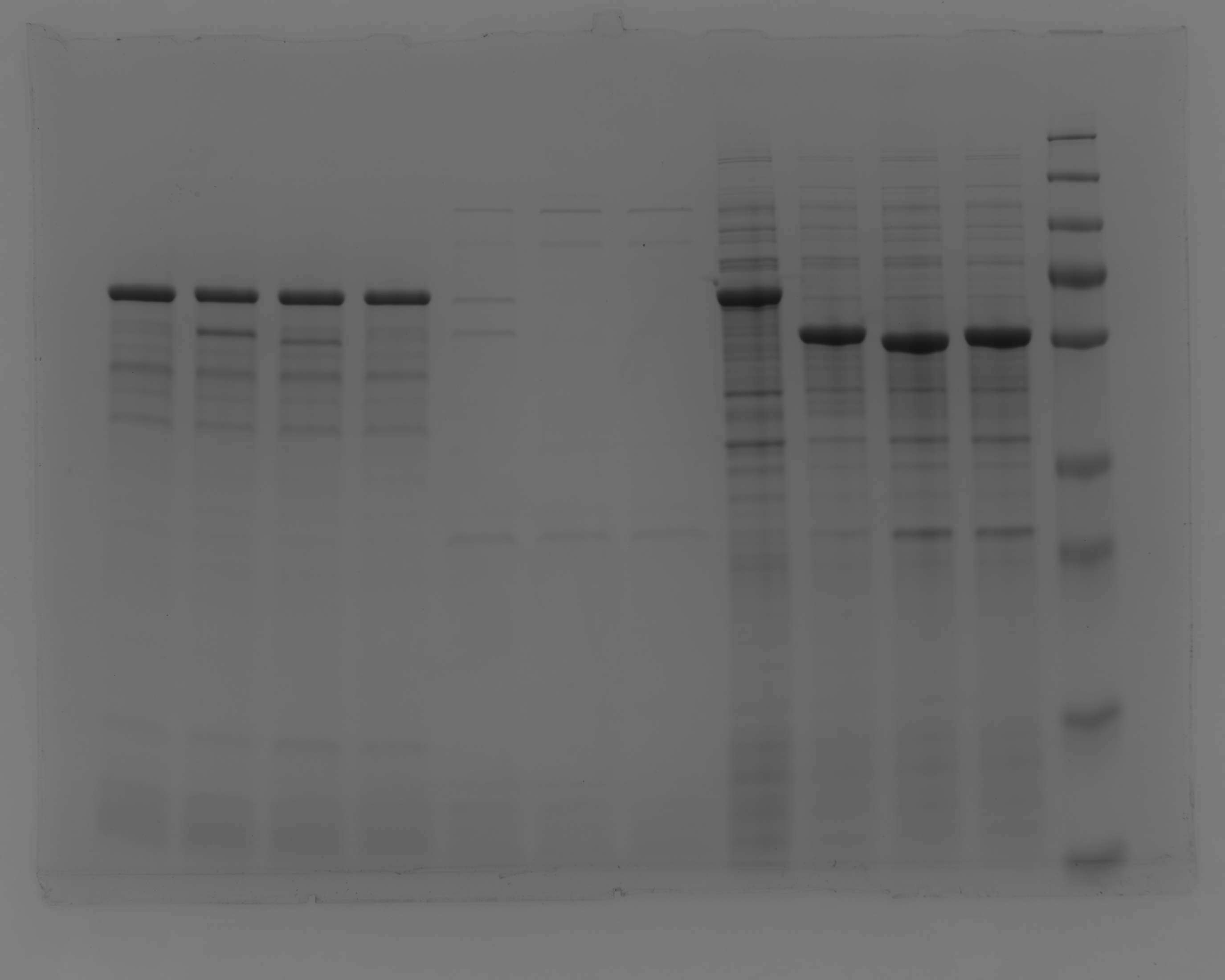

Supplement: Supplementary file 11 — Source Data Fig. 2 [file 44319_2023_6_MOESM11_ESM.zip › Figure 2/2D/Dr eIF4E3/admin1 2022-07-05 10h31m03s(Coomassie Blue).raw16.tif]

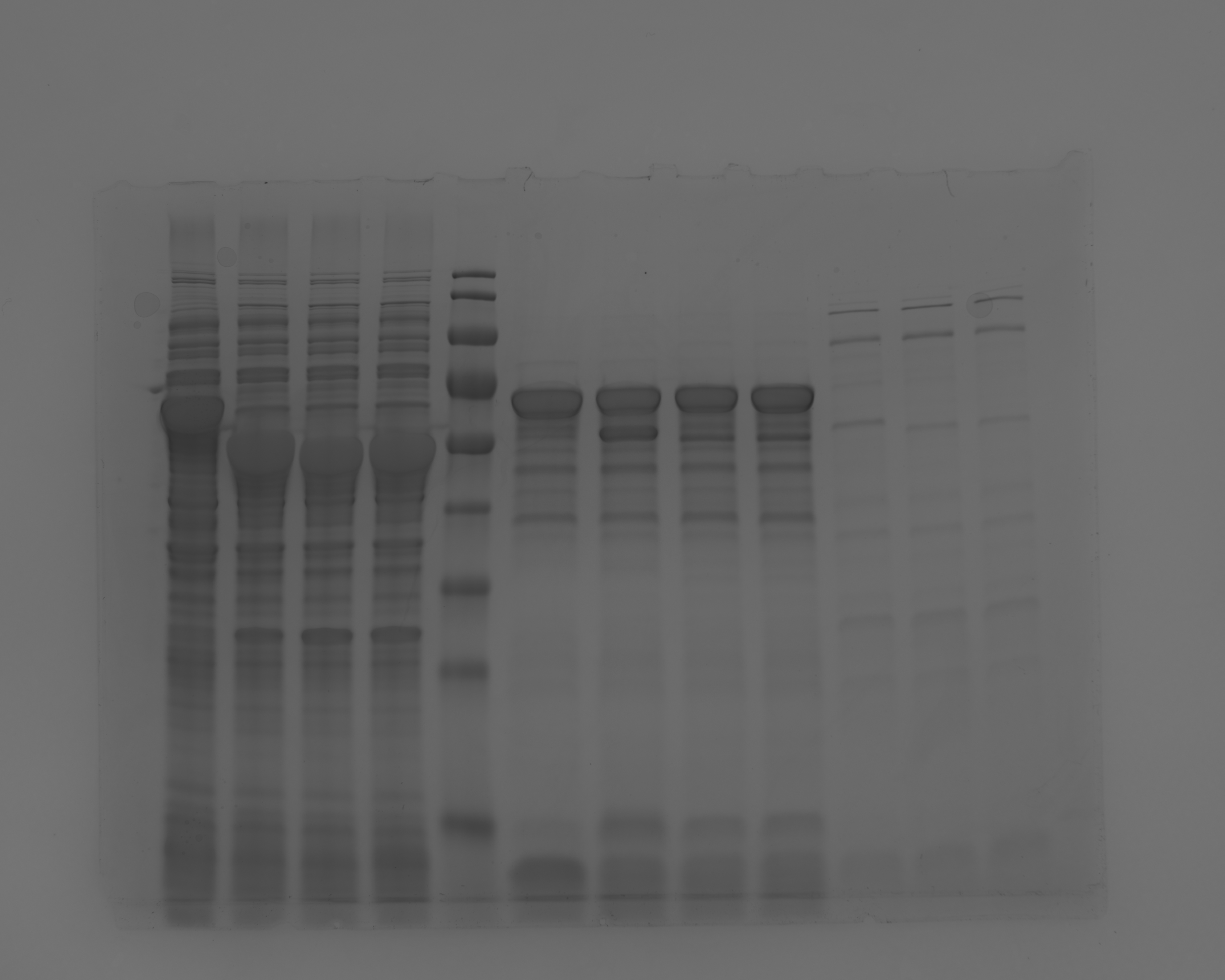

Supplement: Supplementary file 11 — Source Data Fig. 2 [file 44319_2023_6_MOESM11_ESM.zip › Figure 2/2D/Dr eIF4E3/admin1 2021-08-30 09h51m47s(Coomassie Blue).raw16.tif]

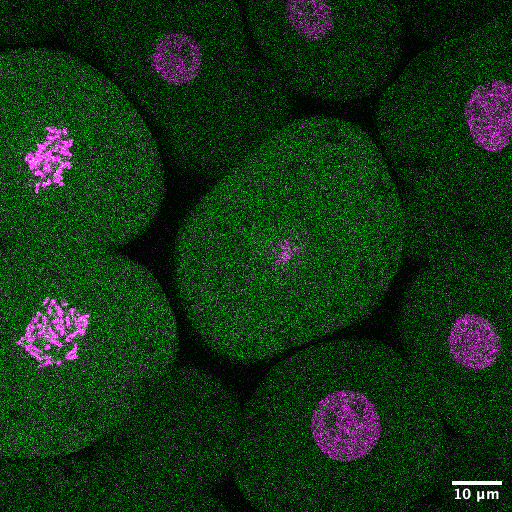

Supplement: Supplementary file 12 — Source Data Fig. 3 [file 44319_2023_6_MOESM12_ESM.zip › Figure 3/3G/3G-right.tif]

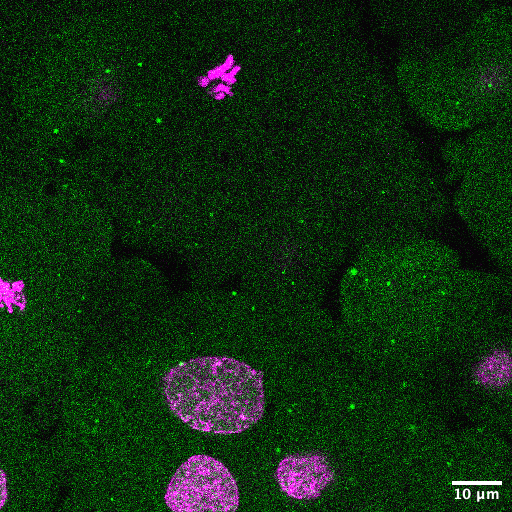

Supplement: Supplementary file 12 — Source Data Fig. 3 [file 44319_2023_6_MOESM12_ESM.zip › Figure 3/3G/3G-left.tif]

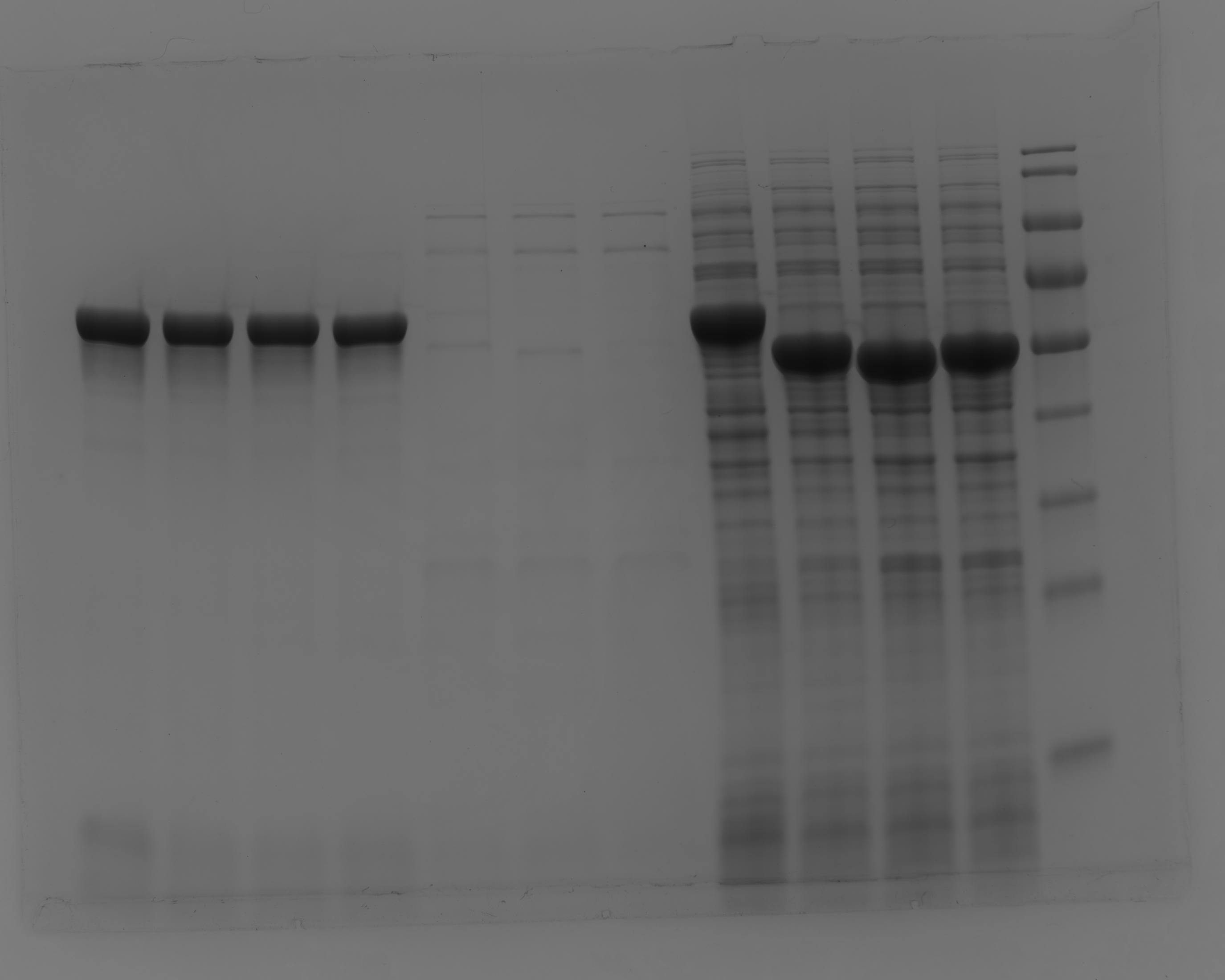

Supplement: Supplementary file 12 — Source Data Fig. 3 [file 44319_2023_6_MOESM12_ESM.zip › Figure 3/3D/Dorsal/admin1 2021-12-08 12h32m39s(Coomassie Blue).raw16.tif]

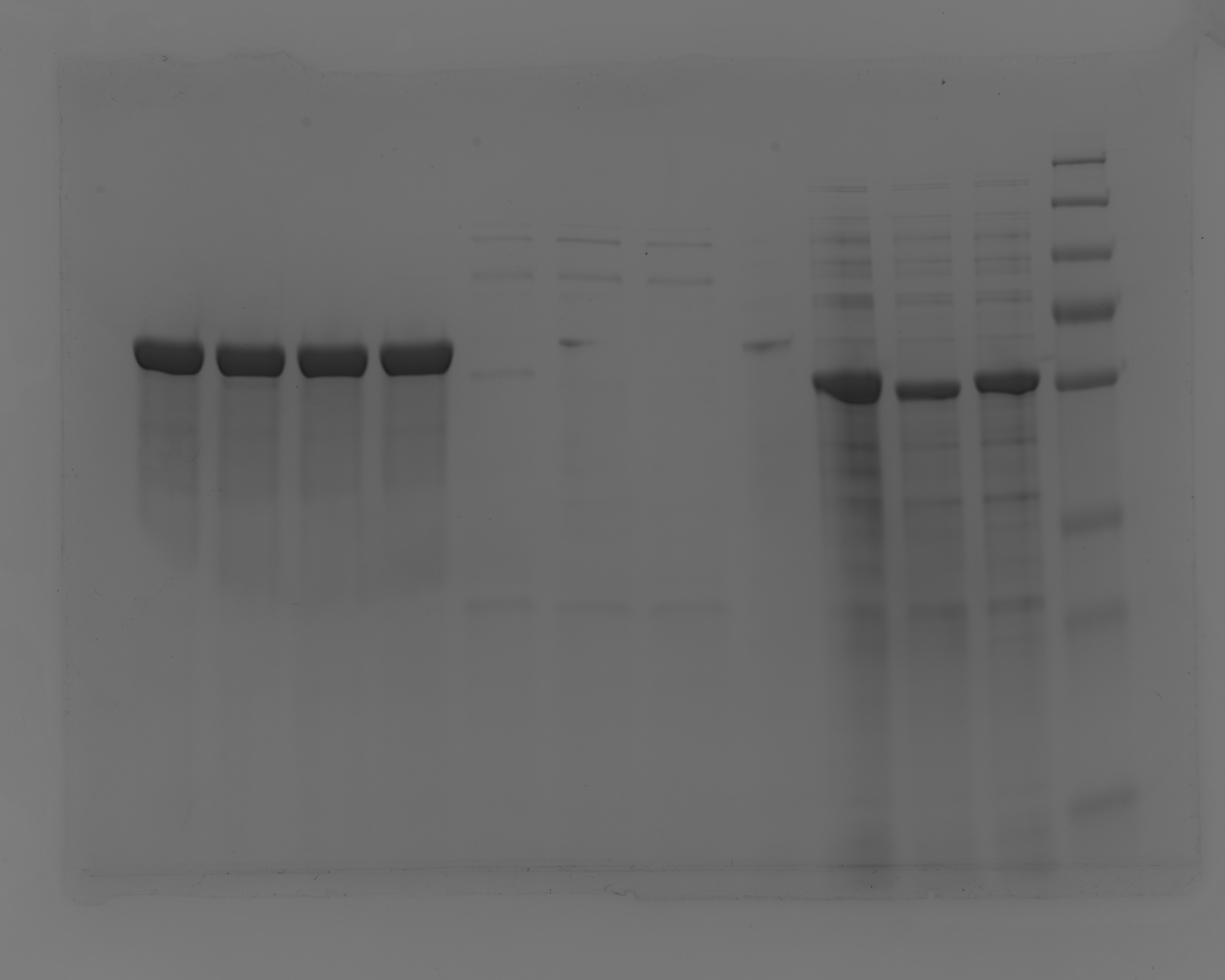

Supplement: Supplementary file 12 — Source Data Fig. 3 [file 44319_2023_6_MOESM12_ESM.zip › Figure 3/3D/Dorsal/admin1 2022-08-17 10h40m32s(Coomassie Blue).raw16.tif]

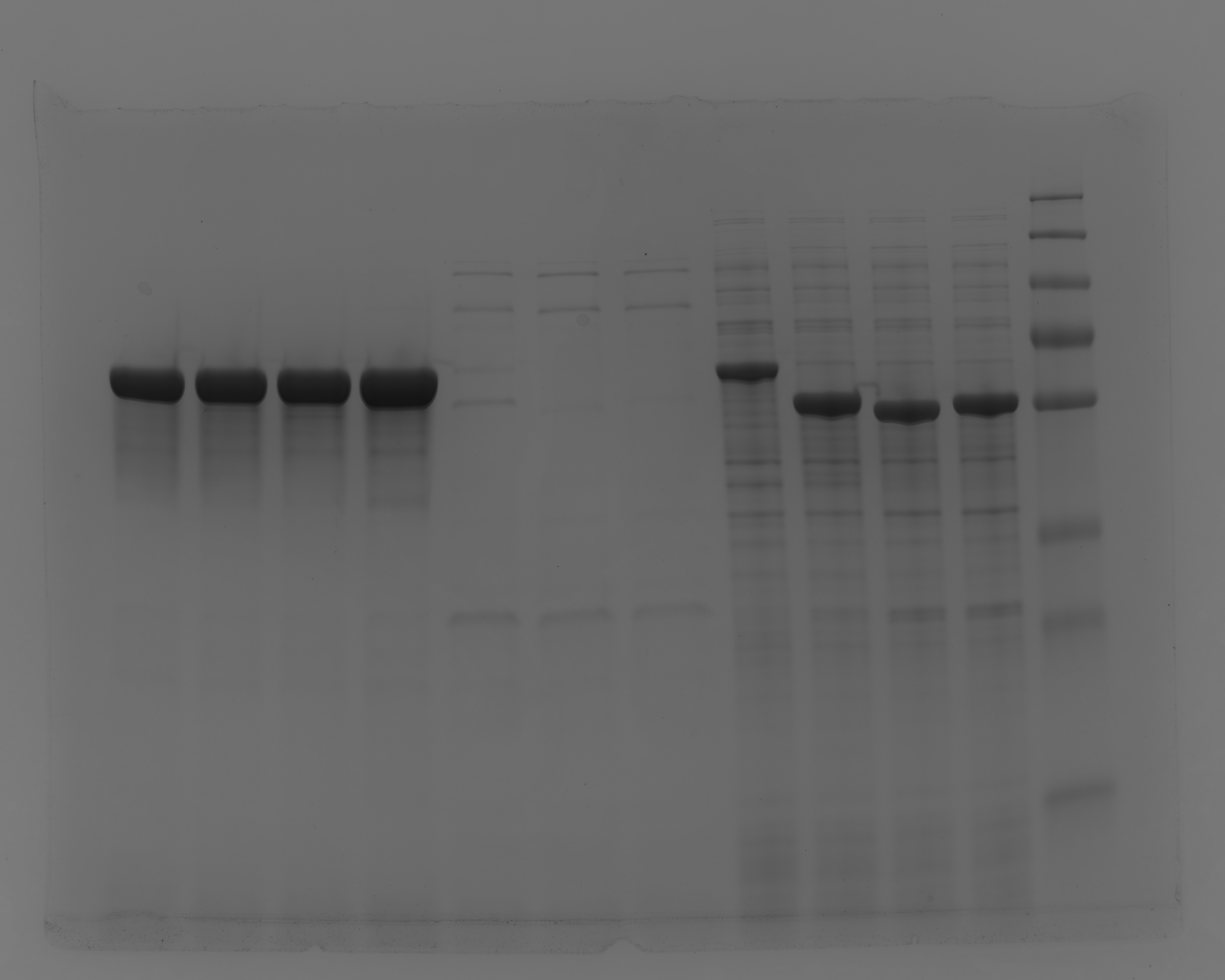

Supplement: Supplementary file 12 — Source Data Fig. 3 [file 44319_2023_6_MOESM12_ESM.zip › Figure 3/3D/Dorsal/admin1 2022-08-18 11h46m18s(Coomassie Blue).raw16.tif]

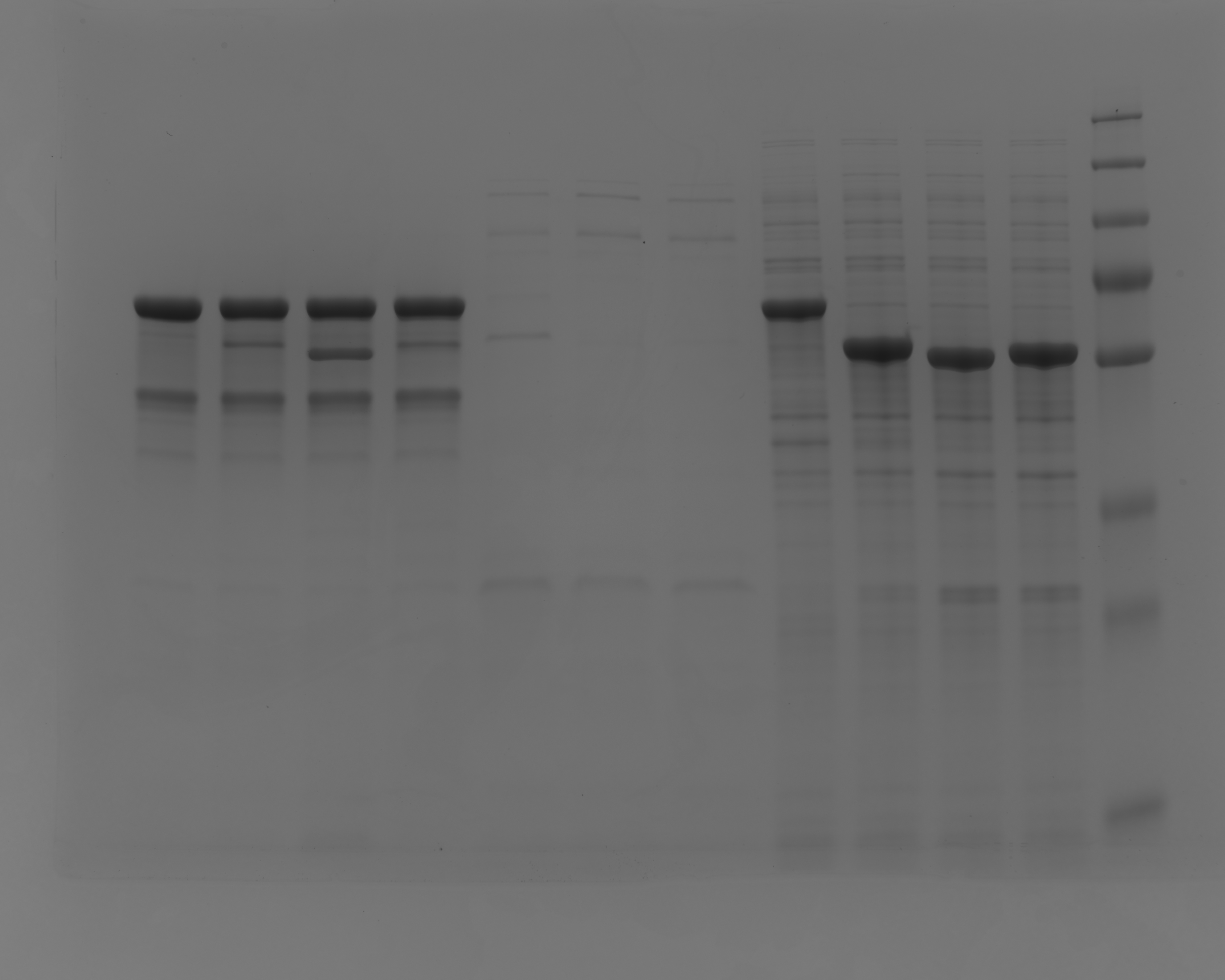

Supplement: Supplementary file 12 — Source Data Fig. 3 [file 44319_2023_6_MOESM12_ESM.zip › Figure 3/3D/eIF4E-eIF4E1B/admin1 2022-05-23 10h54m44s(Coomassie Blue).raw16.tif]

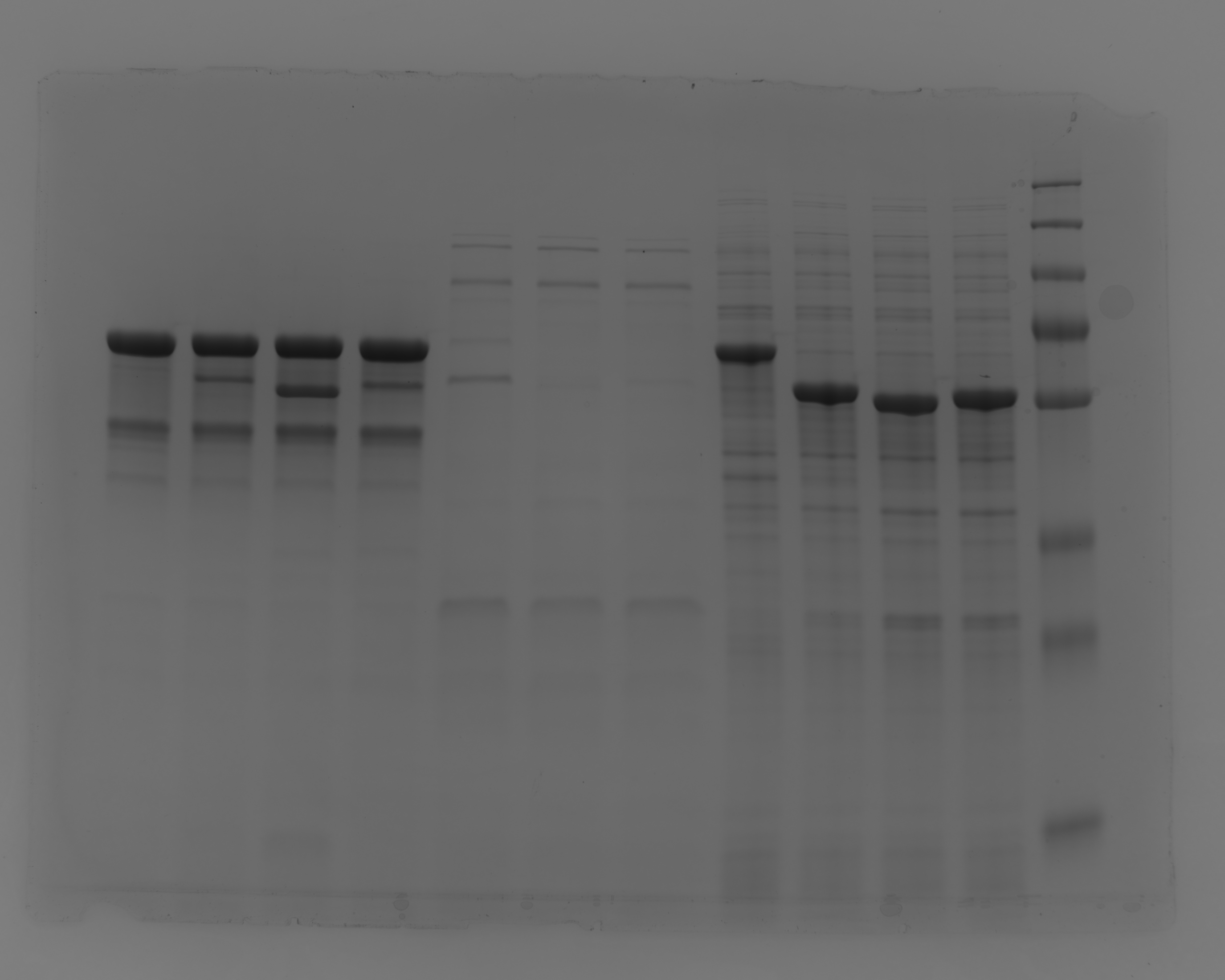

Supplement: Supplementary file 12 — Source Data Fig. 3 [file 44319_2023_6_MOESM12_ESM.zip › Figure 3/3D/eIF4E-eIF4E1B/admin1 2022-05-10 11h11m15s(Coomassie Blue).raw16.tif]

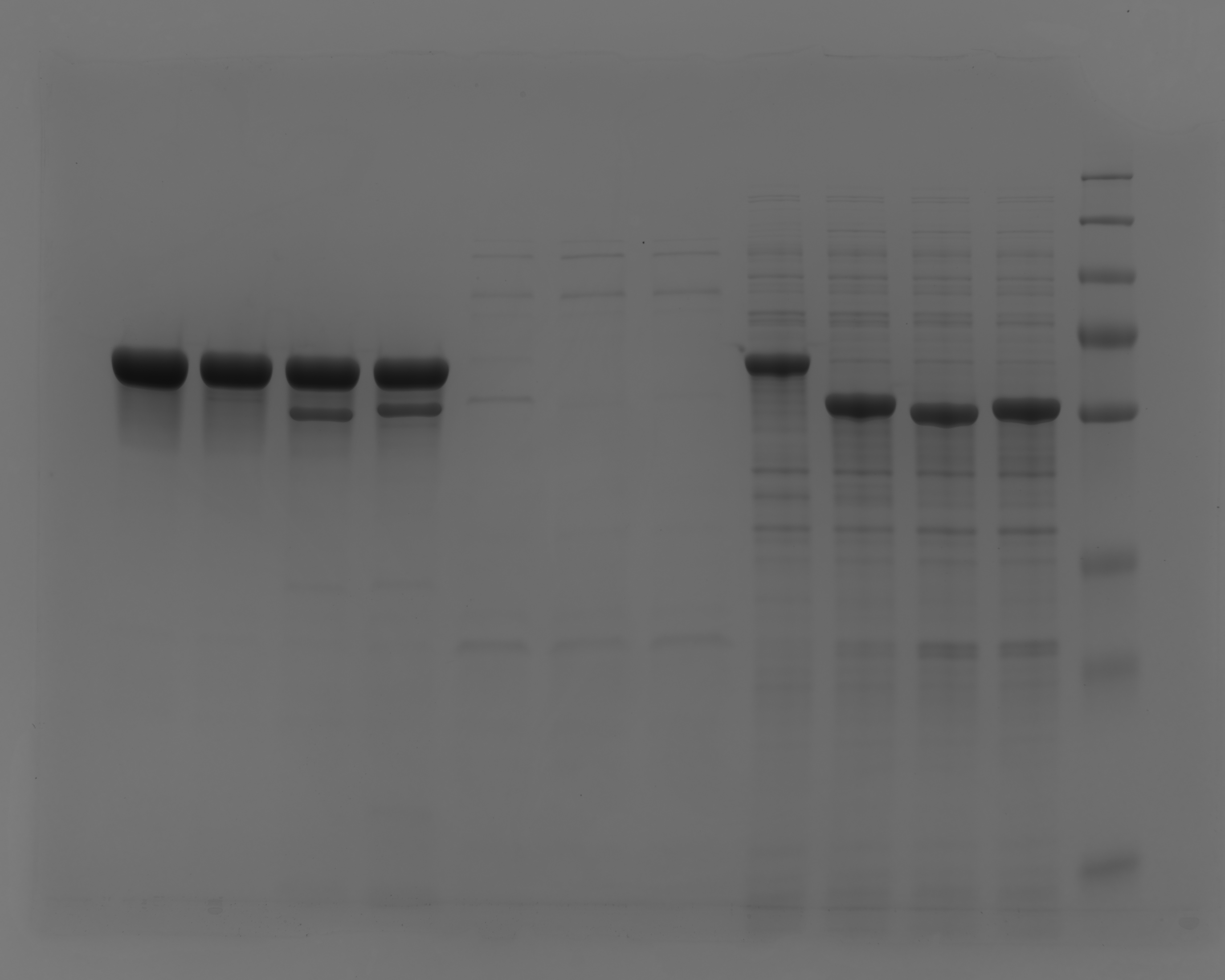

Supplement: Supplementary file 12 — Source Data Fig. 3 [file 44319_2023_6_MOESM12_ESM.zip › Figure 3/3D/eIF4E1B-eIF4E/admin1 2022-05-23 10h56m39s(Coomassie Blue).raw16.tif]

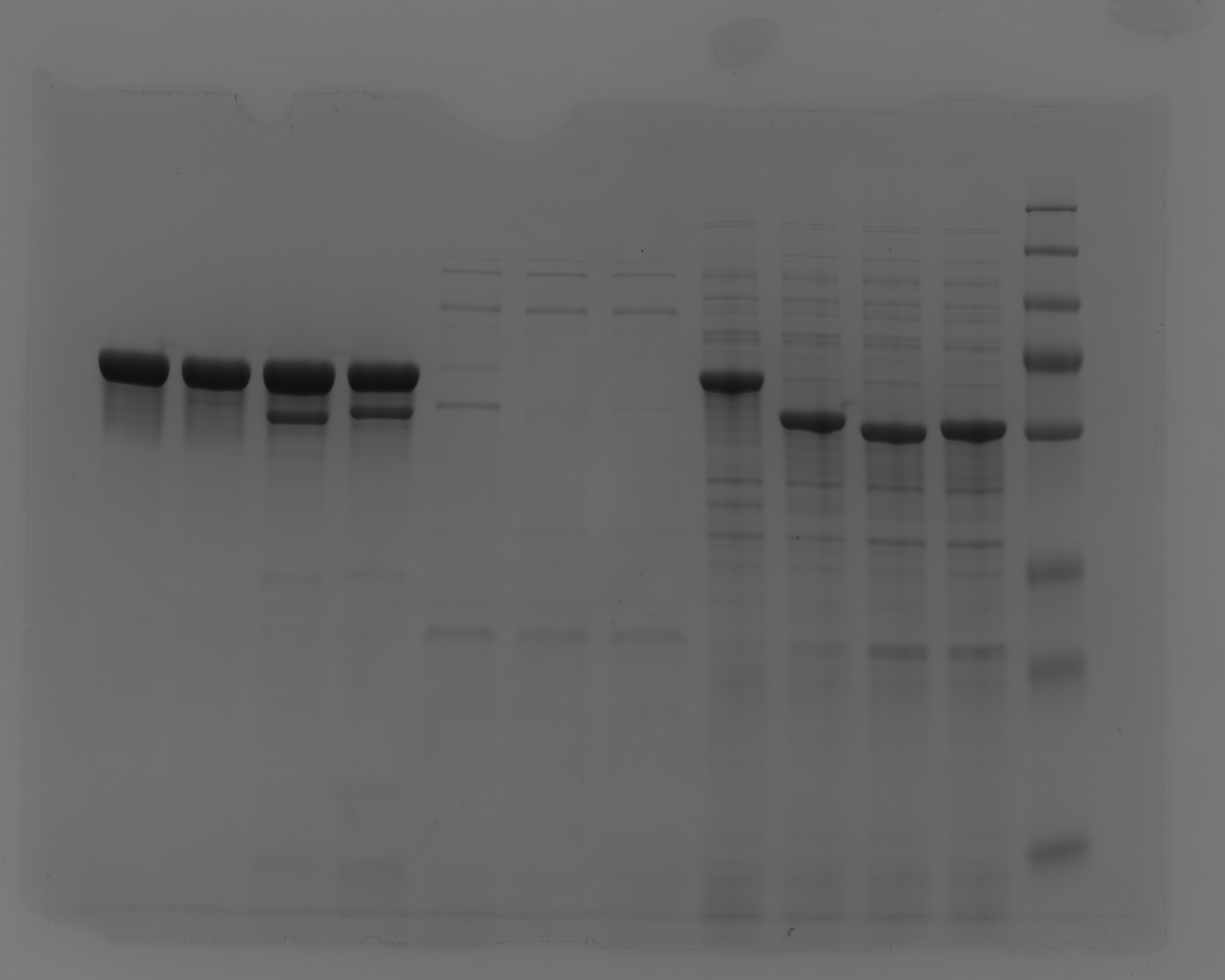

Supplement: Supplementary file 12 — Source Data Fig. 3 [file 44319_2023_6_MOESM12_ESM.zip › Figure 3/3D/eIF4E1B-eIF4E/admin1 2022-05-10 11h13m12s(Coomassie Blue).raw16.tif]

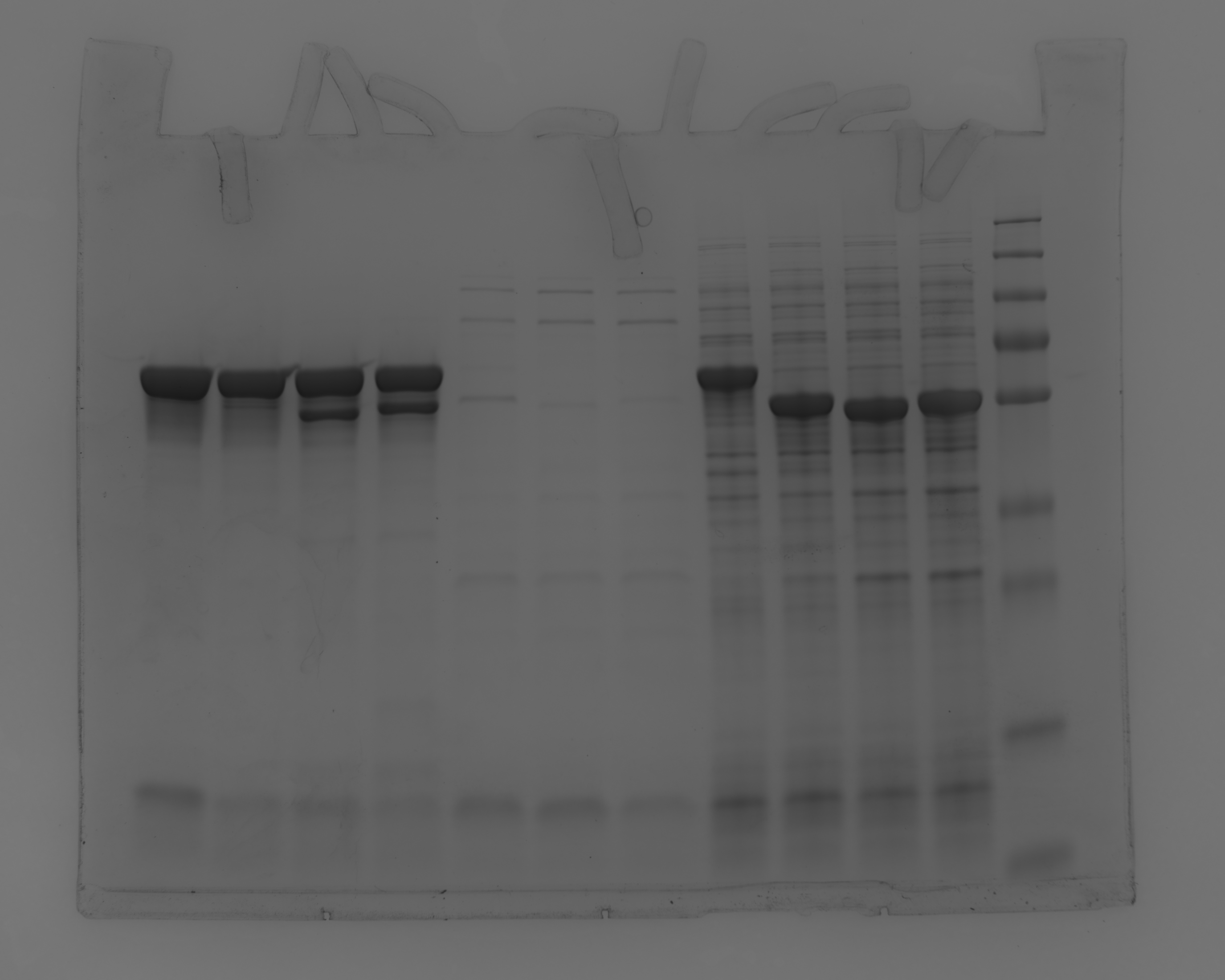

Supplement: Supplementary file 12 — Source Data Fig. 3 [file 44319_2023_6_MOESM12_ESM.zip › Figure 3/3D/eIF4E1B-eIF4E/admin1 2022-04-28 14h26m55s(Coomassie Blue).raw16.tif]

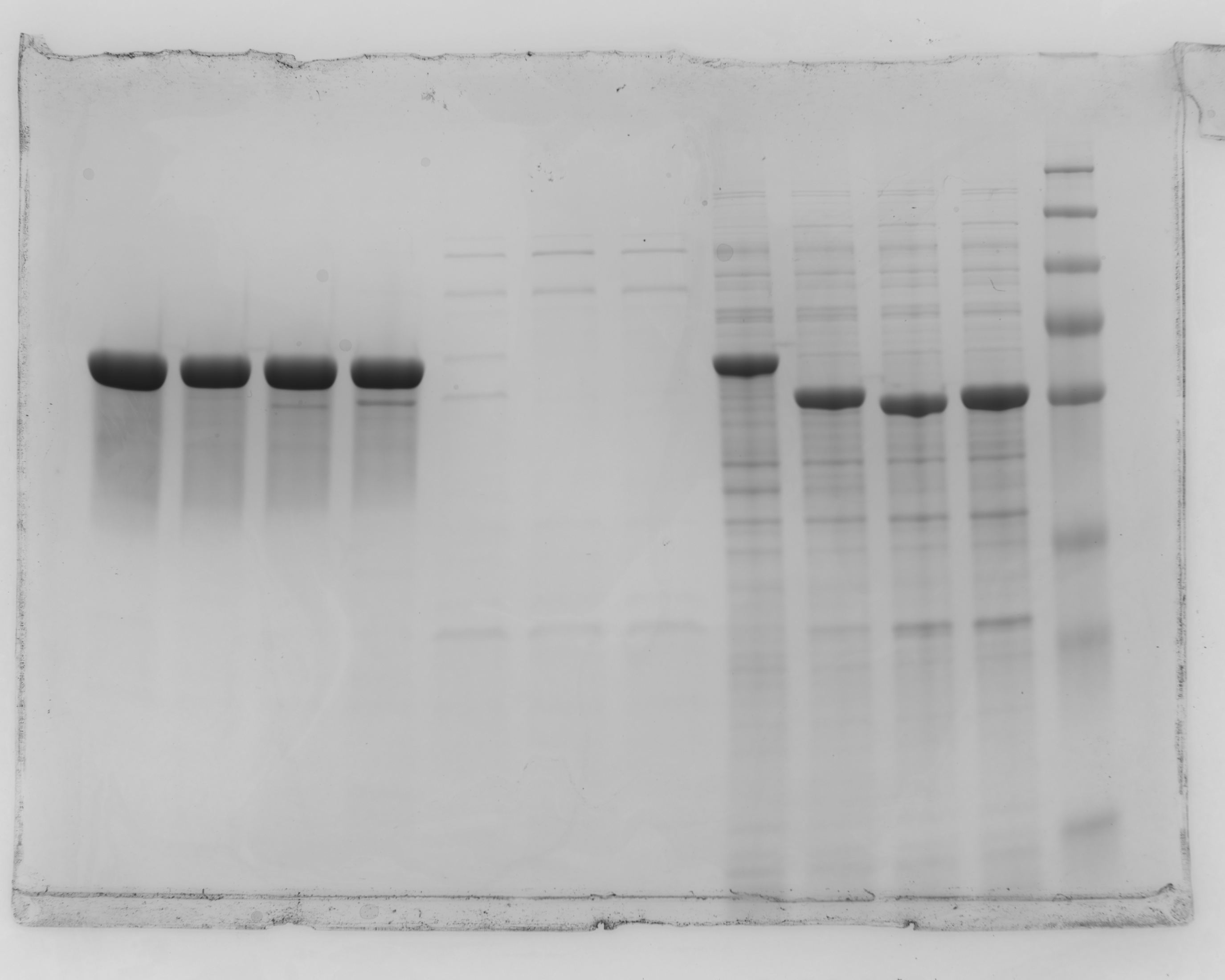

Supplement: Supplementary file 12 — Source Data Fig. 3 [file 44319_2023_6_MOESM12_ESM.zip › Figure 3/3D/Lateral/admin1 2022-06-27 12h33m40s(Coomassie Blue).raw16.tif]

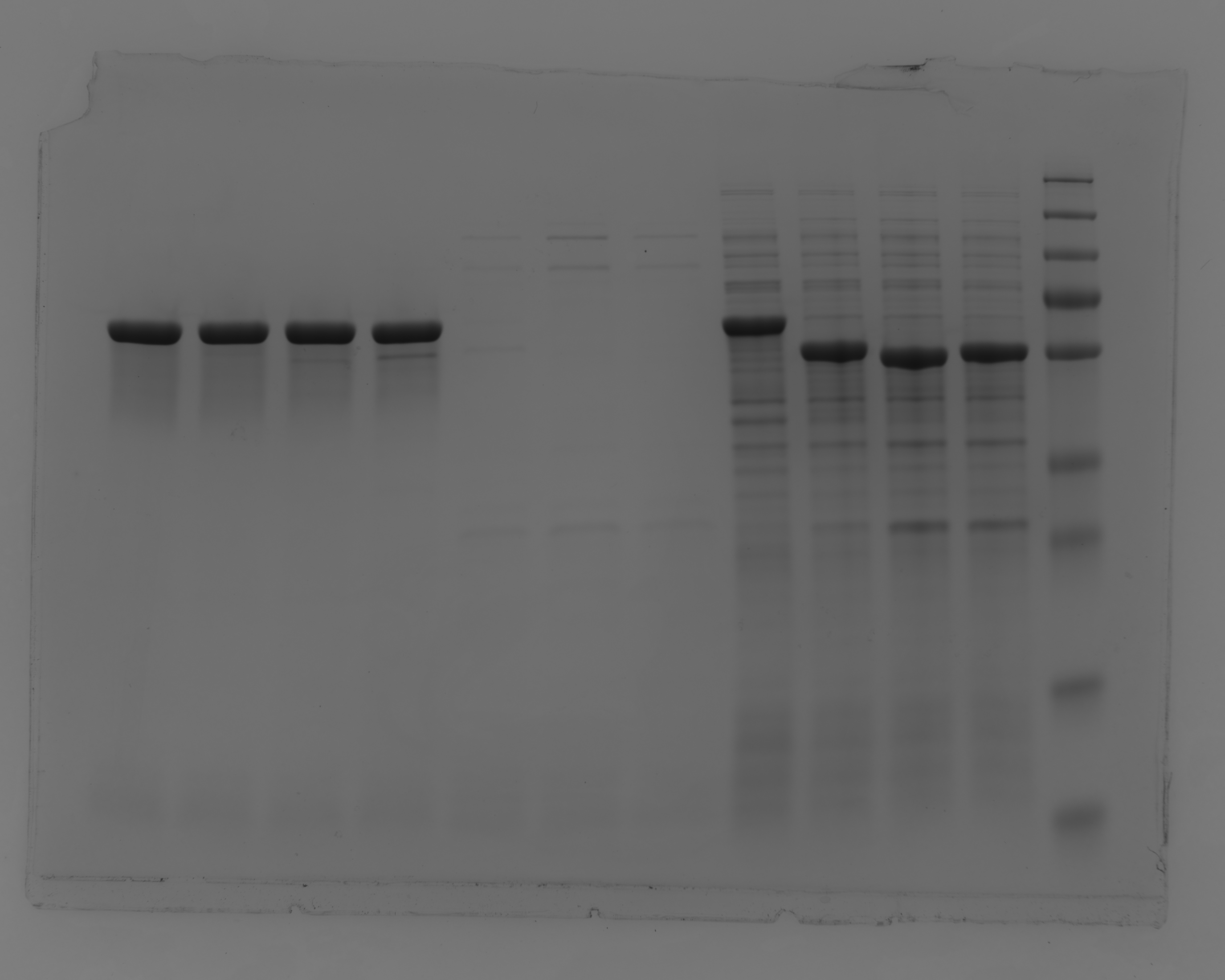

Supplement: Supplementary file 12 — Source Data Fig. 3 [file 44319_2023_6_MOESM12_ESM.zip › Figure 3/3D/Lateral/admin1 2022-06-21 10h32m20s(Coomassie Blue).raw16.tif]

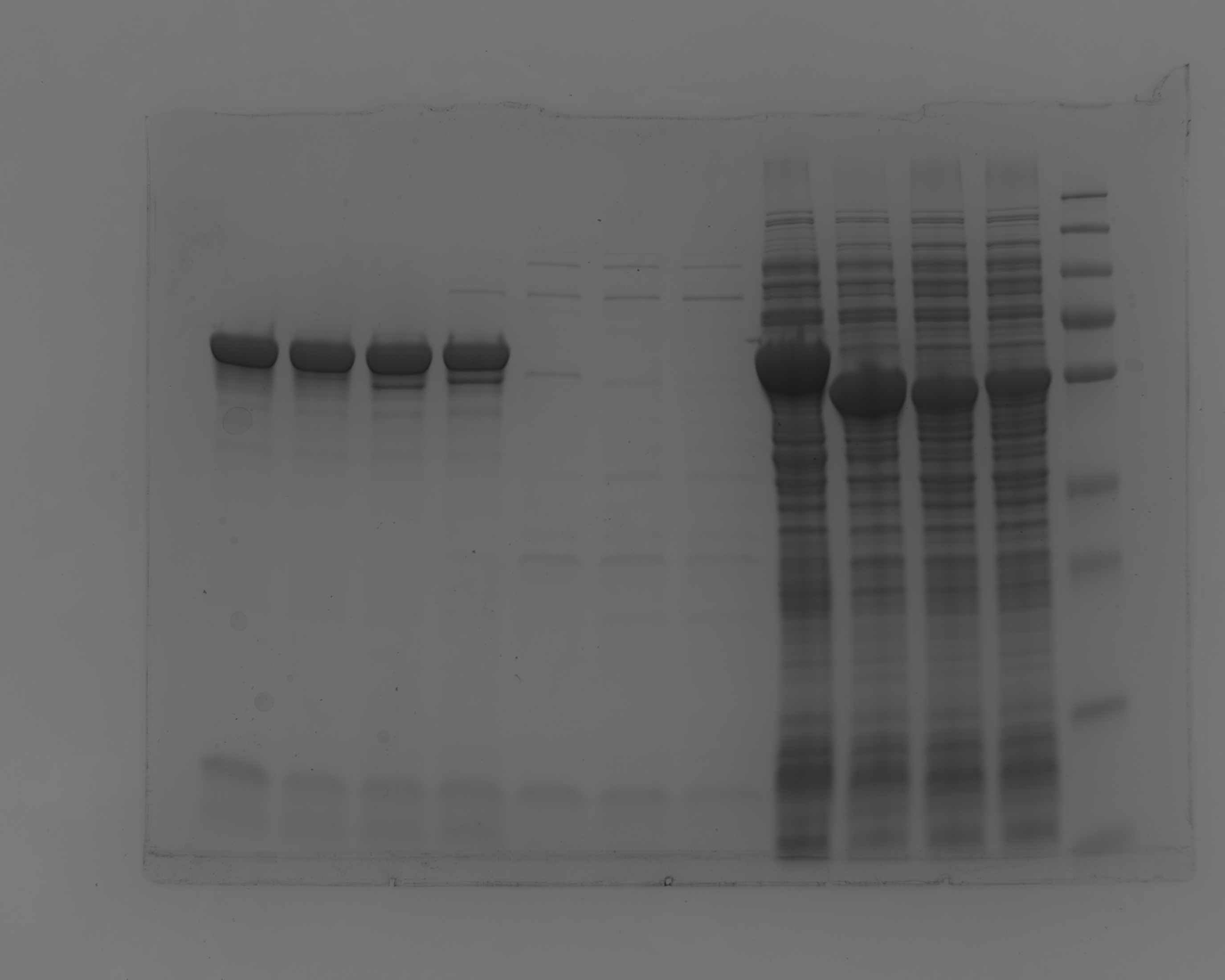

Supplement: Supplementary file 12 — Source Data Fig. 3 [file 44319_2023_6_MOESM12_ESM.zip › Figure 3/3D/Lateral/admin1 2022-03-04 09h07m43s(Coomassie Blue).raw16.tif]

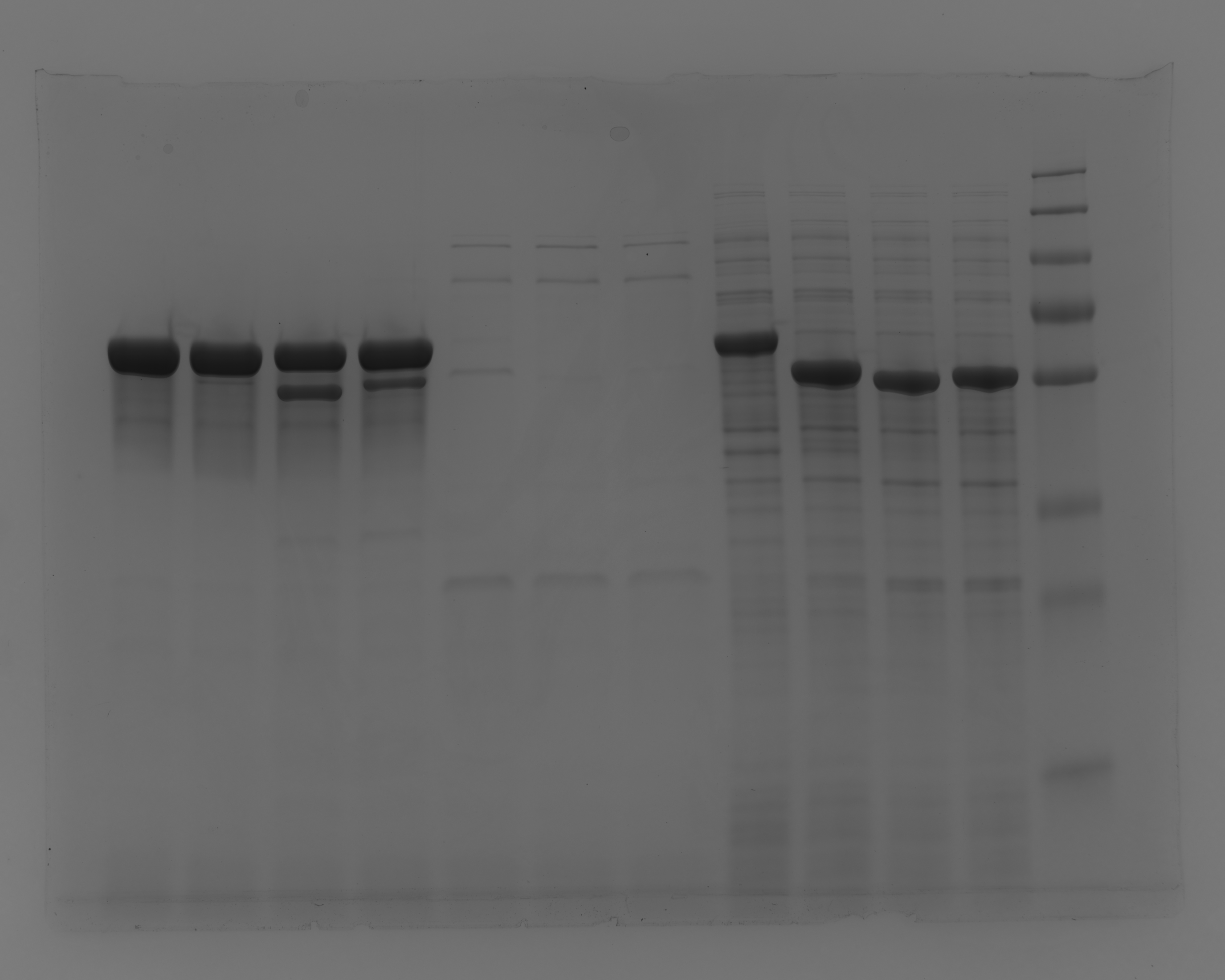

Supplement: Supplementary file 12 — Source Data Fig. 3 [file 44319_2023_6_MOESM12_ESM.zip › Figure 3/3D/K108E/admin1 2022-08-18 11h48m29s(Coomassie Blue).raw16.tif]

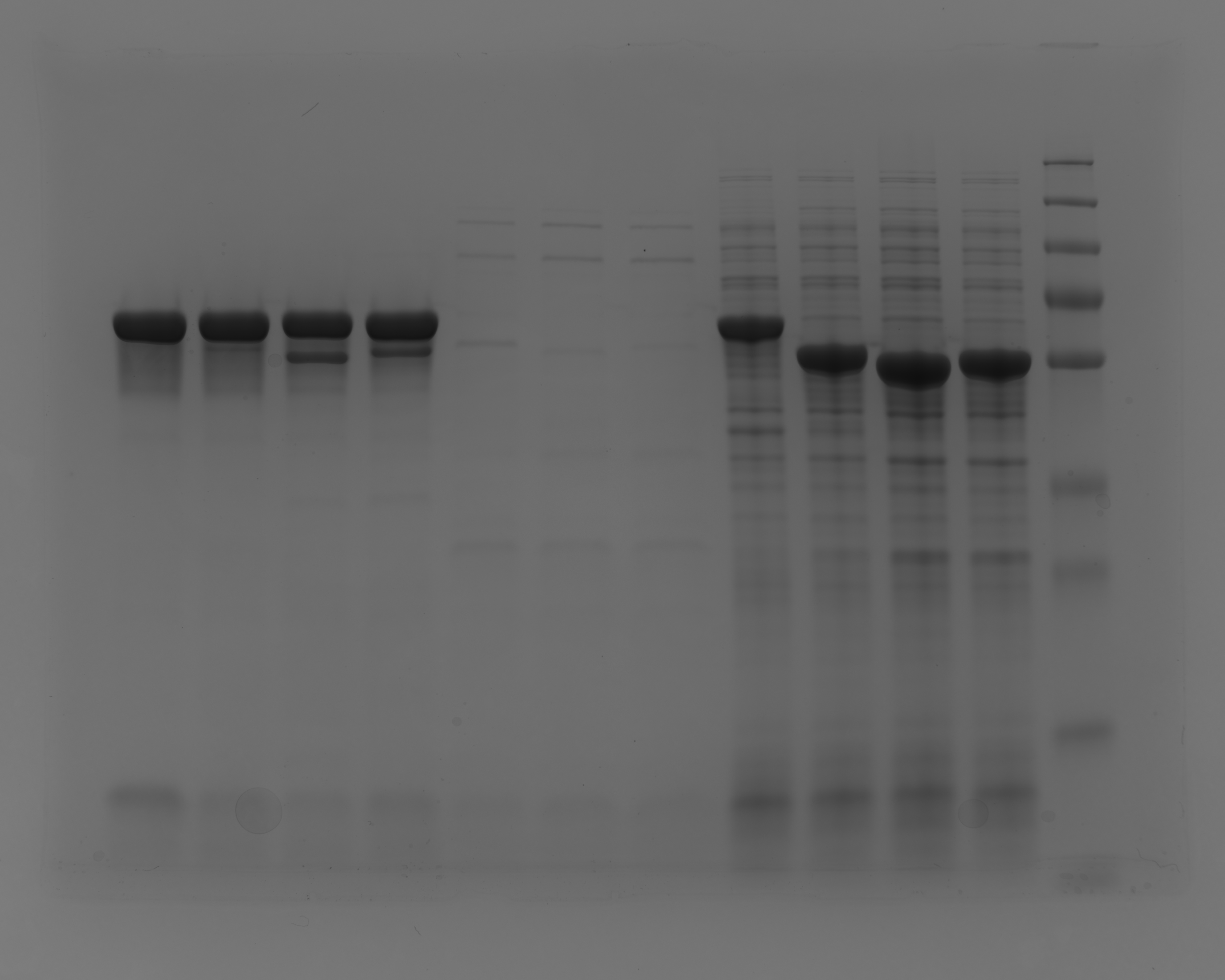

Supplement: Supplementary file 12 — Source Data Fig. 3 [file 44319_2023_6_MOESM12_ESM.zip › Figure 3/3D/K108E/admin1 2022-04-01 17h10m00s(Coomassie Blue).raw16.tif]

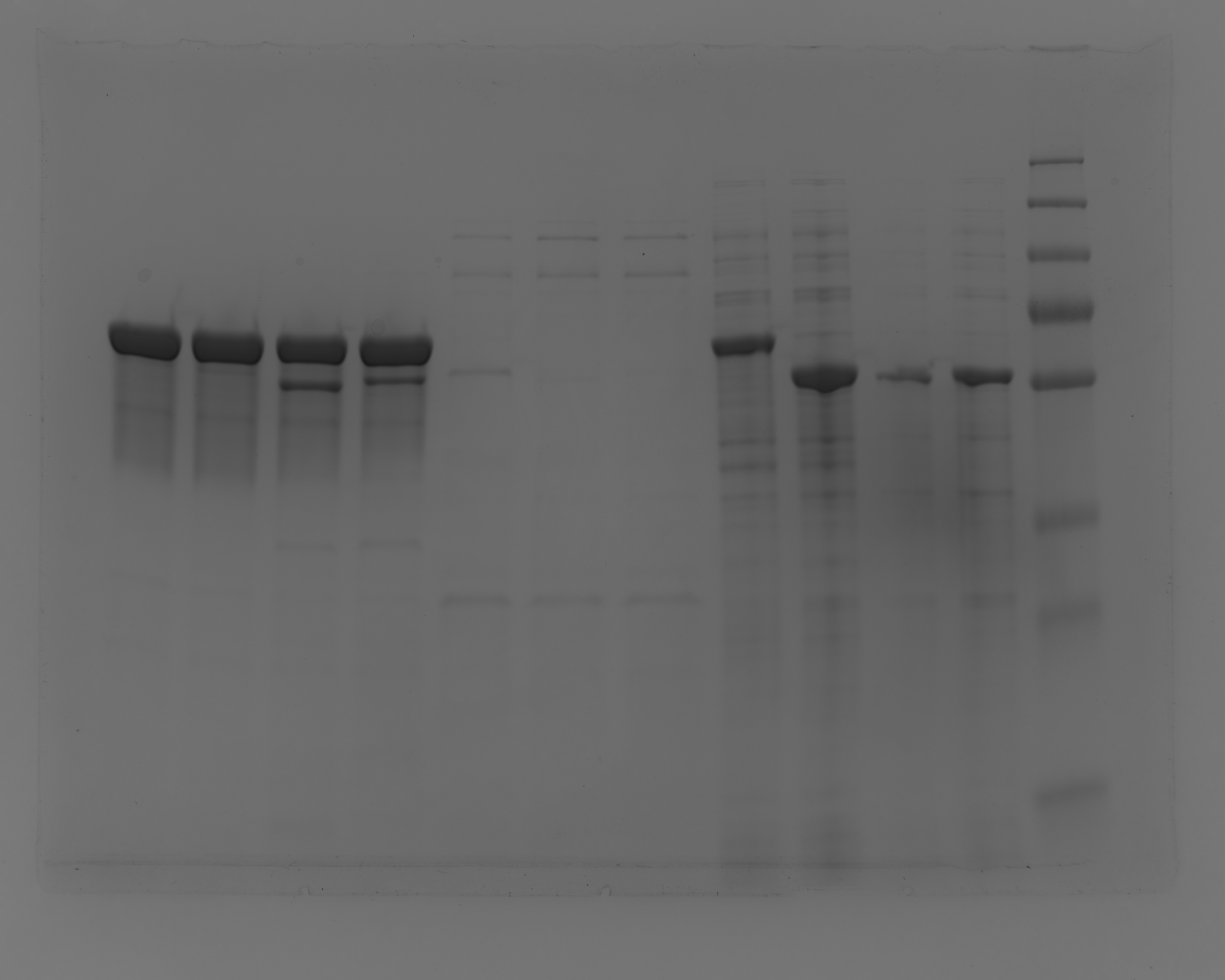

Supplement: Supplementary file 12 — Source Data Fig. 3 [file 44319_2023_6_MOESM12_ESM.zip › Figure 3/3D/K108E/admin1 2022-08-17 10h42m36s(Coomassie Blue).raw16.tif]

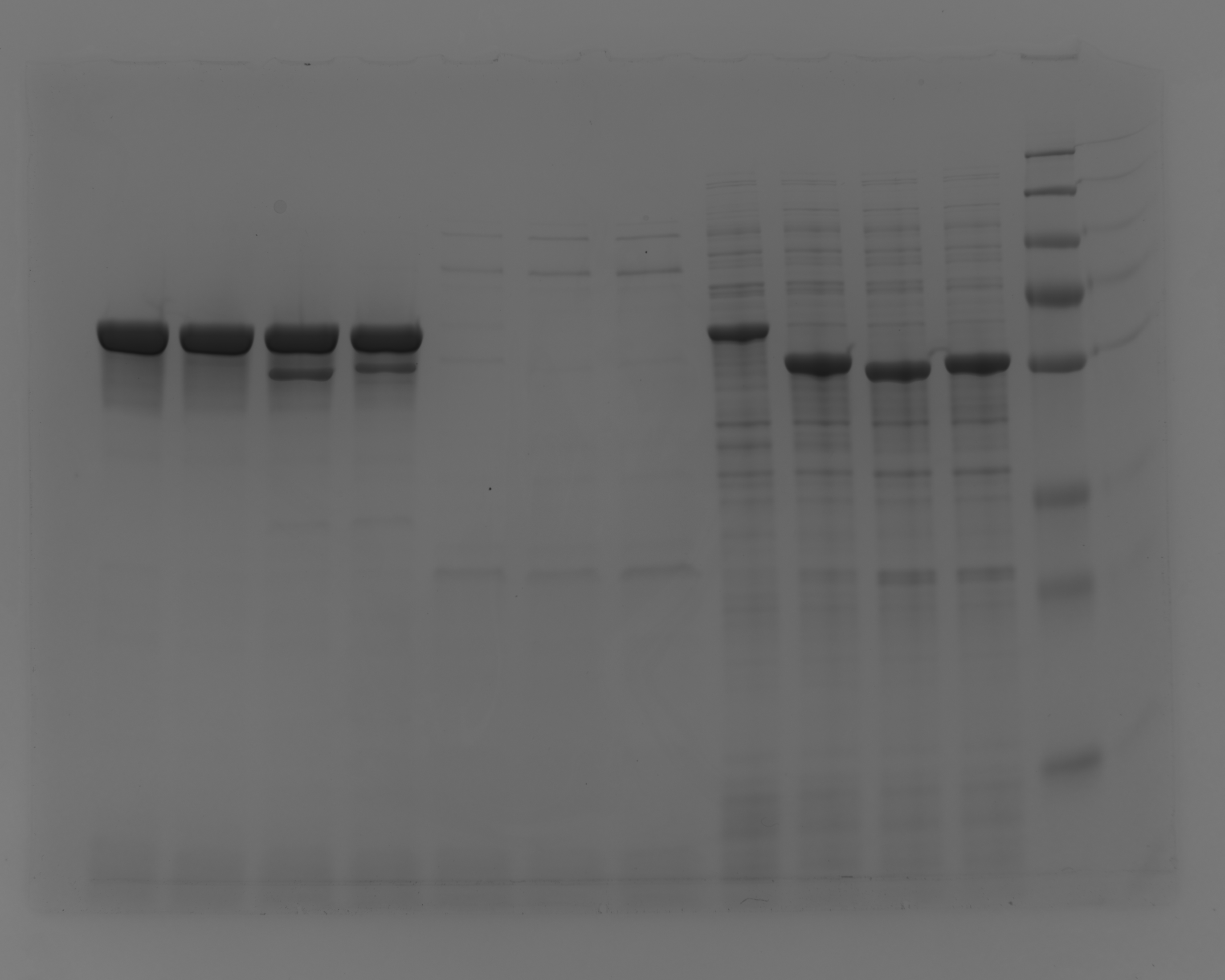

Supplement: Supplementary file 12 — Source Data Fig. 3 [file 44319_2023_6_MOESM12_ESM.zip › Figure 3/3D/KSHN/admin1 2022-05-20 10h43m42s(Coomassie Blue).raw16.tif]

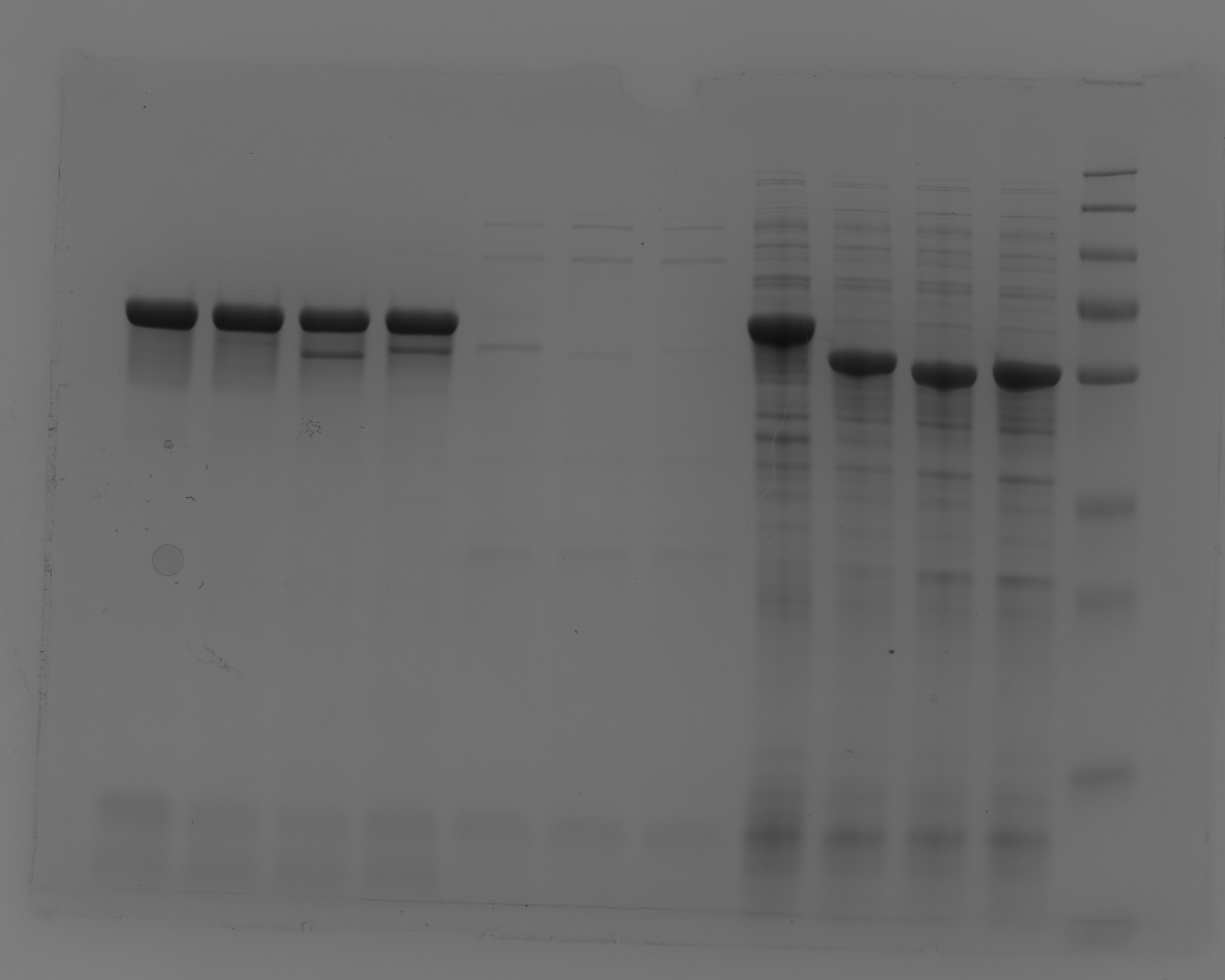

Supplement: Supplementary file 12 — Source Data Fig. 3 [file 44319_2023_6_MOESM12_ESM.zip › Figure 3/3D/KSHN/admin1 2022-04-04 09h32m23s(Coomassie Blue).raw16.tif]

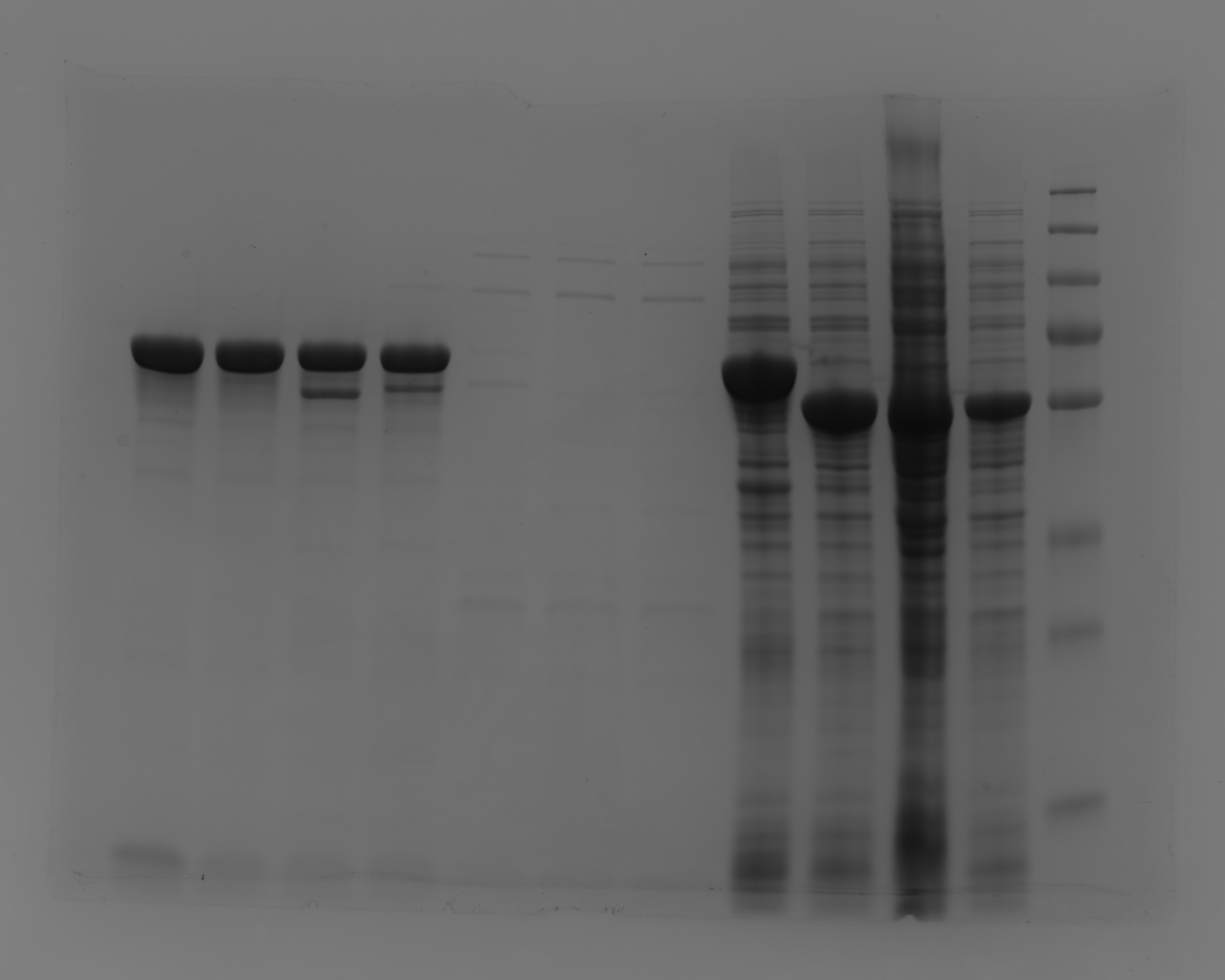

Supplement: Supplementary file 12 — Source Data Fig. 3 [file 44319_2023_6_MOESM12_ESM.zip › Figure 3/3D/KSHN/admin1 2022-02-23 11h44m46s(Coomassie Blue).raw16.tif]

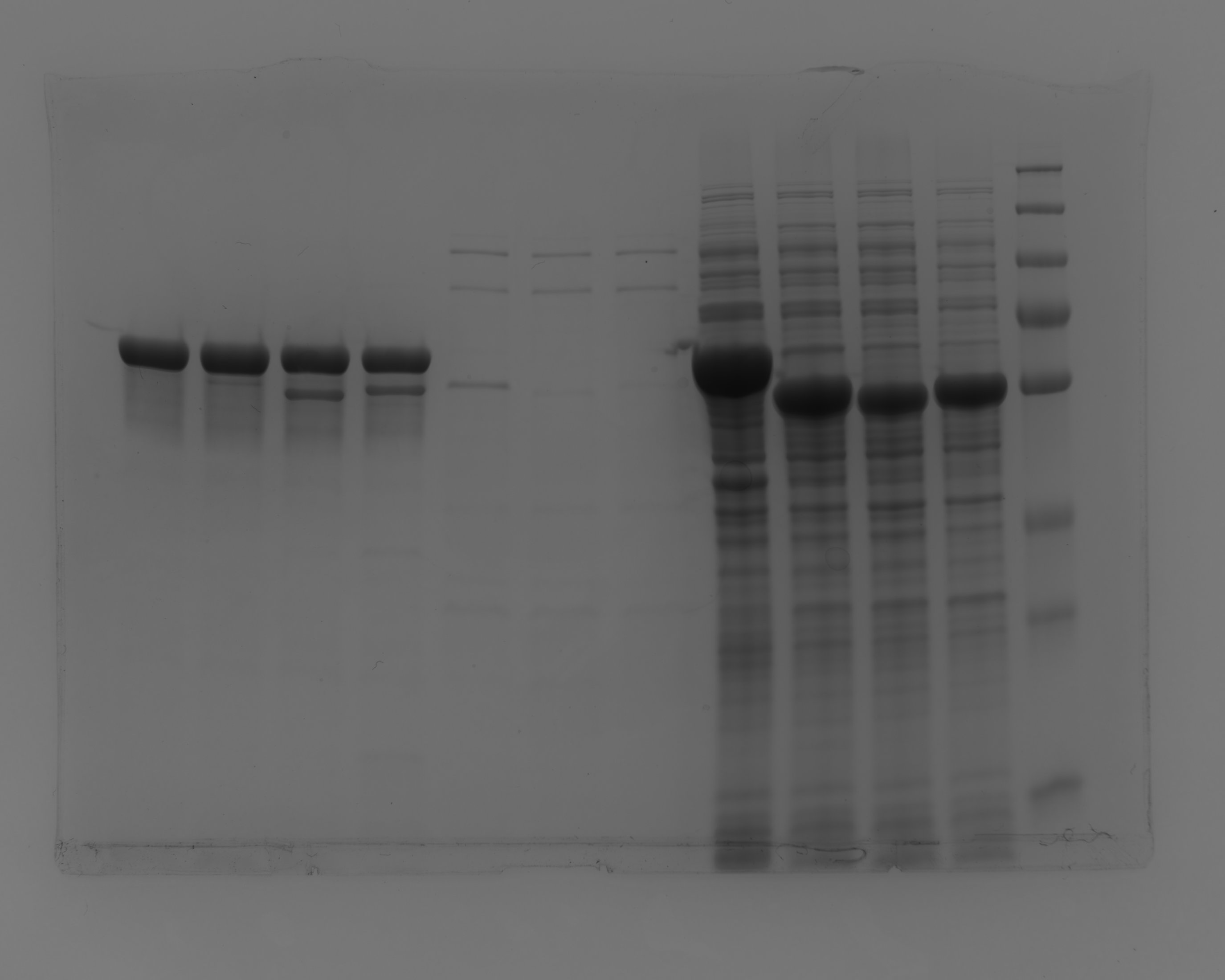

Supplement: Supplementary file 12 — Source Data Fig. 3 [file 44319_2023_6_MOESM12_ESM.zip › Figure 3/3D/K112N/admin1 2022-01-24 09h03m36s(Coomassie Blue).raw16.tif]

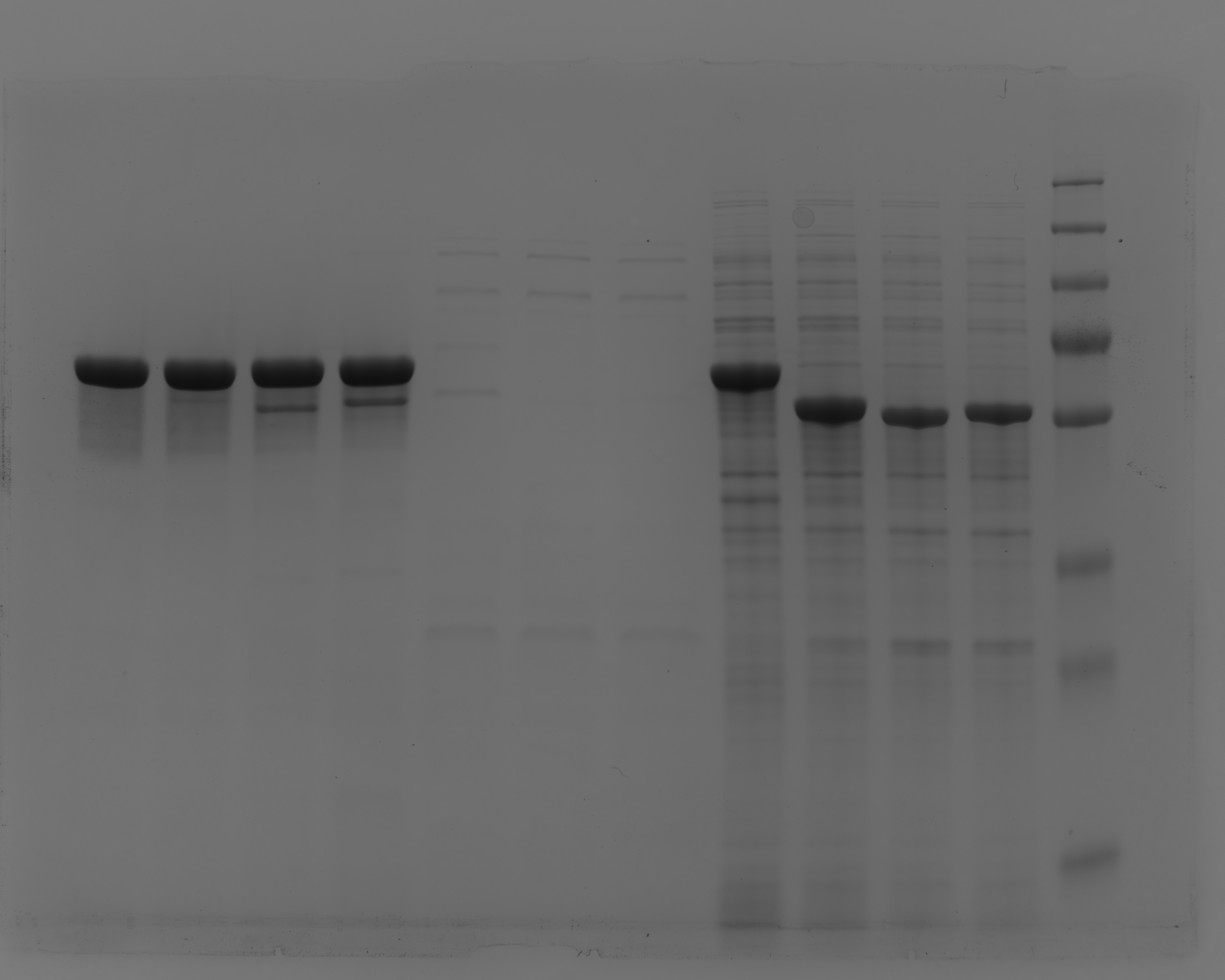

Supplement: Supplementary file 12 — Source Data Fig. 3 [file 44319_2023_6_MOESM12_ESM.zip › Figure 3/3D/K112N/admin1 2022-05-16 08h31m46s(Coomassie Blue).raw16.tif]

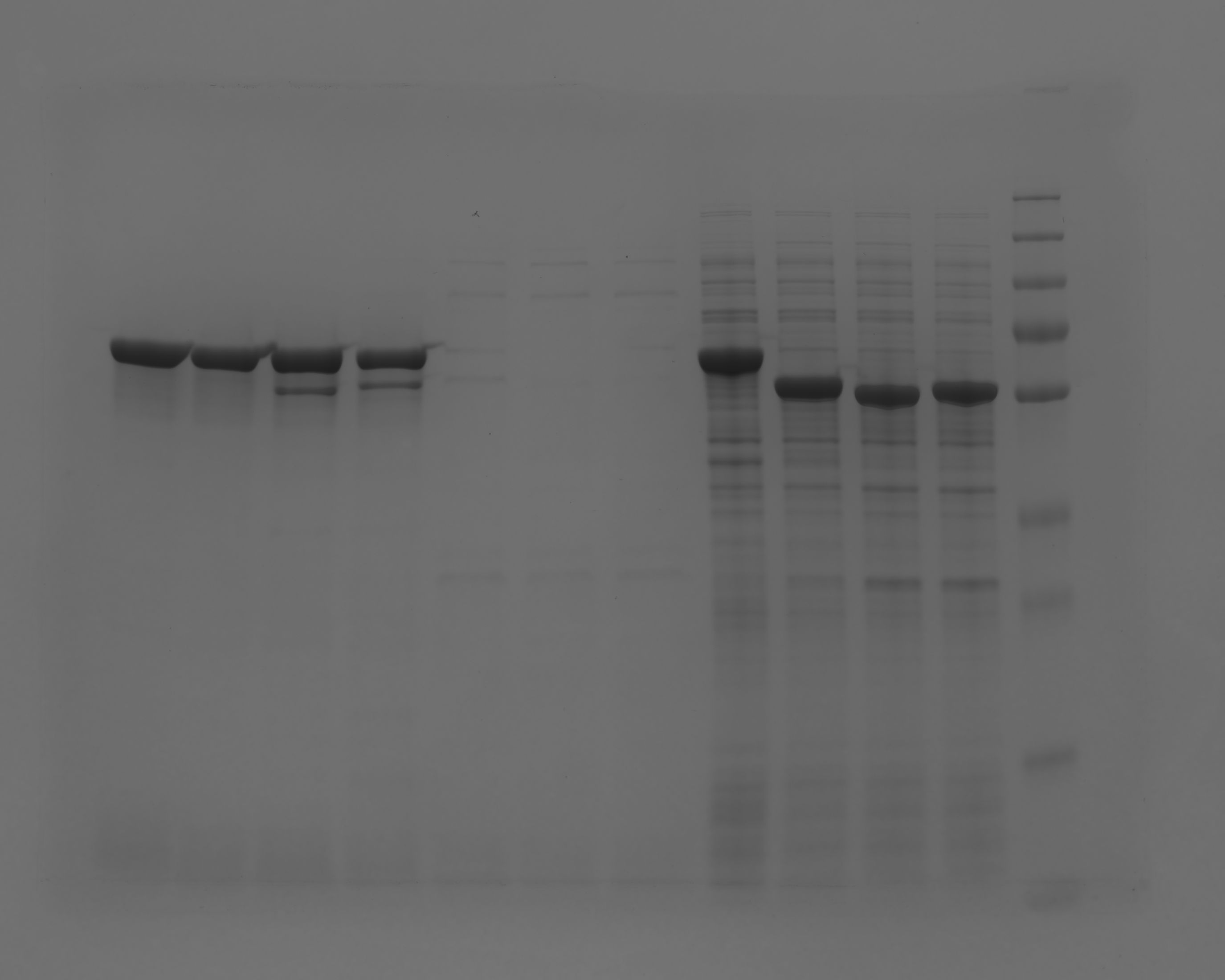

Supplement: Supplementary file 12 — Source Data Fig. 3 [file 44319_2023_6_MOESM12_ESM.zip › Figure 3/3D/K112N/test 2022-06-07 09h03m00s(Coomassie Blue).raw16.tif]

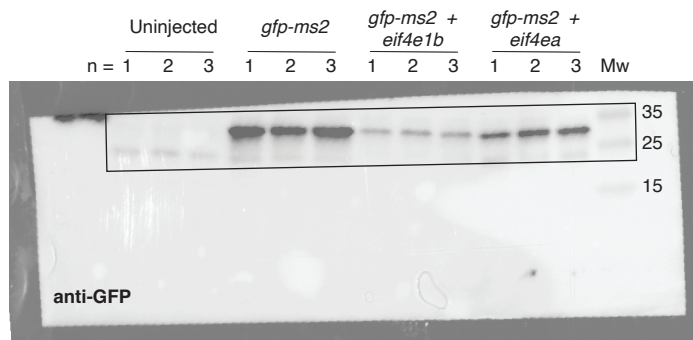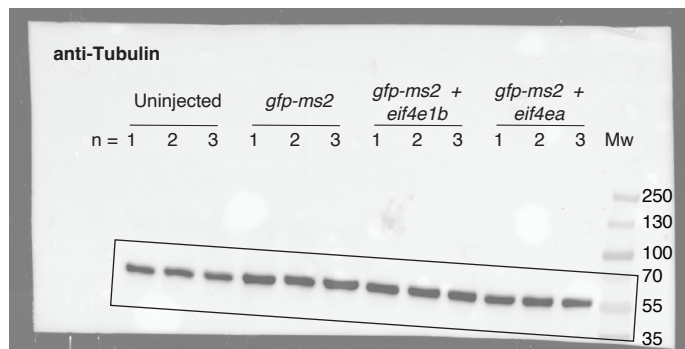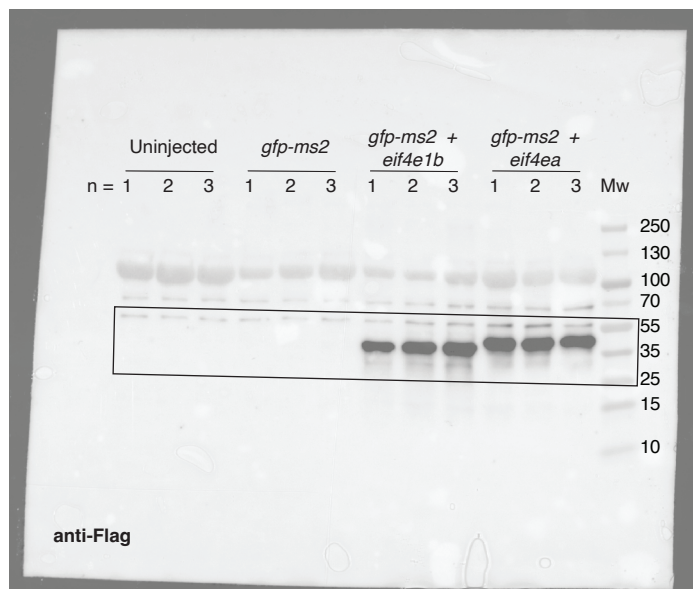

Supplement: Supplementary file 13 — Source Data Fig. 5 [file 44319_2023_6_MOESM13_ESM.zip › Figure 5/5G_blots.pdf]
