# Supplementary material for: eIF4E1b is a non-canonical eIF4E protecting maternal dormant mRNAs
Source: EMBO Rep. 2023 Dec 14;25(1):23. doi: 10.1038/s44319-023-00006-4 (PMC10883267; doi:10.1038/s44319-023-00006-4)
Supplement: Supplementary file 11 — Source Data Fig. 2 [file 44319_2023_6_MOESM11_ESM.zip › Figure 2/2D/README.rtf]

Folders contain Coomassie-stained gels of 3 replicates obtained from independent experiments (raw images used for quantification).The order of lanes is as described in fig2D_example-gel.pdf, except for Dr eIF4Ea 2021-08-02, Mm eIF4E1B 2021-09-17, and Dr eIF4E3 2021-08-02, where the order is:1) eIF4E lysate2) eIF4G lysate3) eIF4EBP1 lysate4) eIF4ENIF1 lysate5) Marker6) eIF4E pulldown7) eIF4E + eIF4EG pulldown8) eIF4E + eIF4EBP1 pulldown9) eIF4E + eIF4ENIF1 pulldown10) eIF4G pulldown11) eIF4EBP1 pulldown12) eIF4ENIF1 pulldown
