# Supplementary material for: eIF4E1b is a non-canonical eIF4E protecting maternal dormant mRNAs
Source: EMBO Rep. 2023 Dec 14;25(1):23. doi: 10.1038/s44319-023-00006-4 (PMC10883267; doi:10.1038/s44319-023-00006-4)
Supplement: Supplementary file 15 — Expanded View Figures [file 44319_2023_6_MOESM15_ESM.pdf]

Expanded View Figures

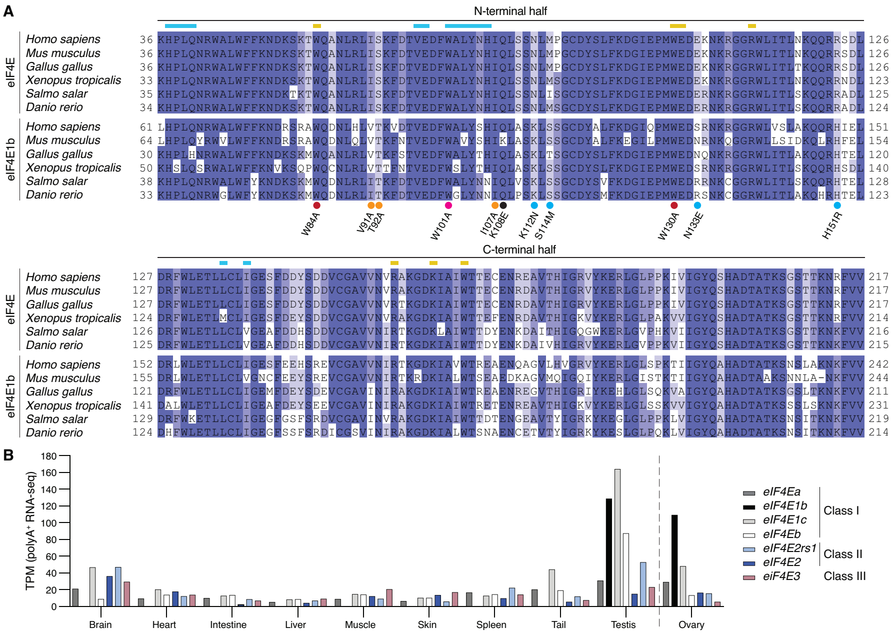

Figure EV1. eIF4E1b and eIF4E are highly conserved but have specific expression patterns.

(**A**) Amino acid alignment of eIF4E and eIF4E1b proteins from six vertebrate species. Unstructured N-terminal regions (see Fig. 1A), which are not included in the constructs used for expressing recombinant proteins, are excluded from the alignment. Residues interacting with the mRNA cap or with eIF4E-binding motifs are highlighted with yellow or blue lines, respectively. eIF4E1B residues mutated in Fig. 3D are indicated with dots (red: mRNA cap binding; pink: dorsal site; orange: lateral site; blue: conserved in eIF4E but different in eIF4E1b proteins; black: others). N-terminal and C-terminal regions exchanged in chimeric constructs are indicated. (**B**) mRNA levels (in transcripts per million, TPM) of zebrafish eIF4Es in different organs and adult tissues based on polyadenine-selected RNA-seq data (Fujihara et al, 2021) (ovary RNA-seq data from Herberg et al, 2018).

© The Author(s)

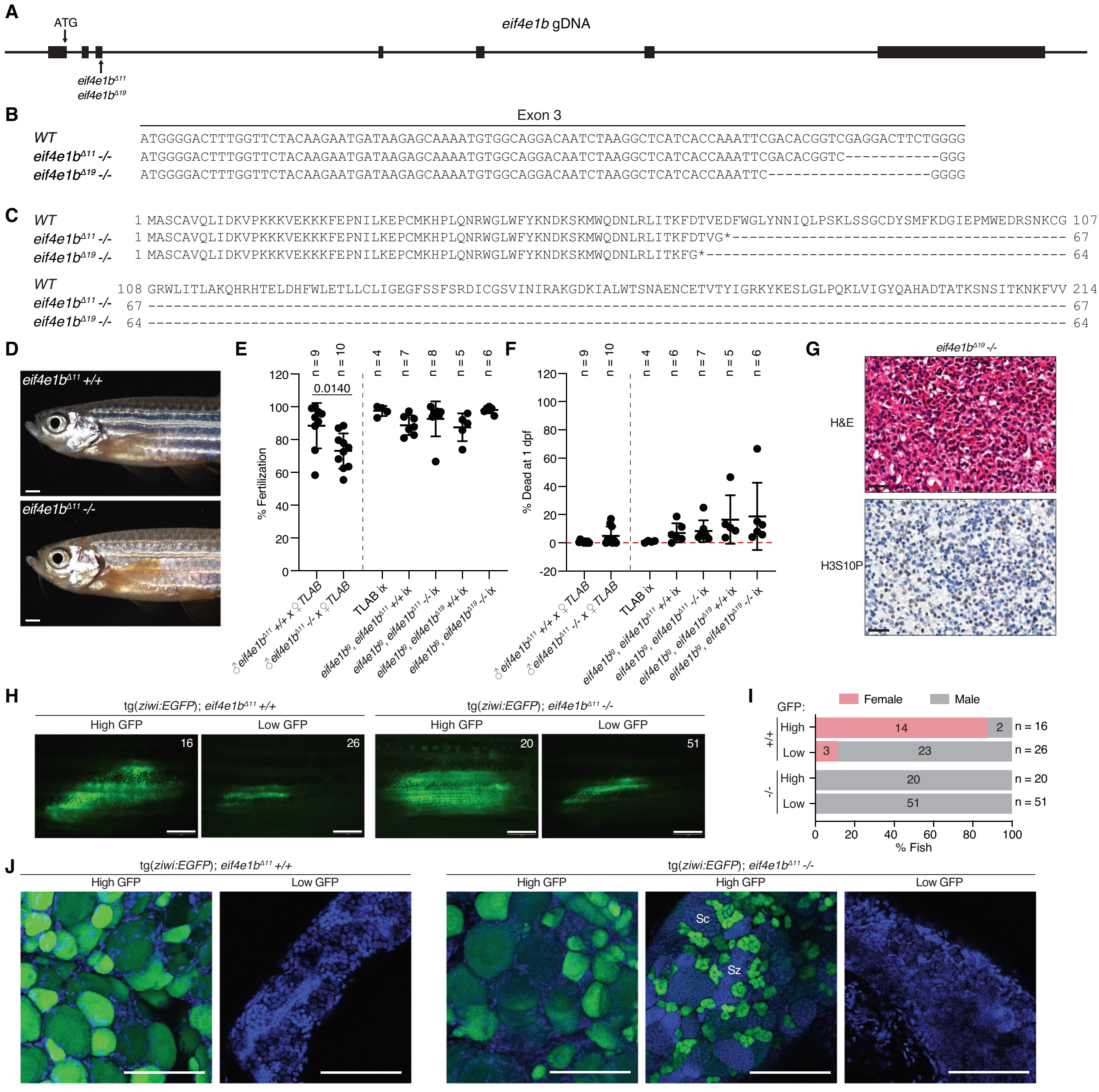

Figure EV2. ***eif4e1b* mutants develop into fertile males due to sex reversal.**

(**A**) Scheme of the zebrafish *eif4e1b* locus, indicating the start codon (*ATG*) and the 11 and 19 nt deletions present in the two *eif4e1b* mutants (*eif4e1b*$^{\Delta 11}$ and *eif4e1b*$^{\Delta 19}$, respectively) generated in this study. Exons are depicted as boxes, introns as lines. (**B**) Nucleotides missing in the third exon of *eif4e1b* mutants. (**C**) eIF4E1b mRNA translation in *eif4e1b* mutants result in truncated proteins of 67 (for *eif4e1b*$^{\Delta 11}$) and 64 (for *eif4e1b*$^{\Delta 19}$) amino acids. Asterisks indicate premature stop codons. (**D**) Representative images of wild-type and *eif4e1b*$^{\Delta 11}$ mutant sibling males obtained from a heterozygous incross (scale bars = 1 mm). (**E**) Fertilization rates of embryos obtained by crossing *eif4e1b*$^{\Delta 11}$ homozygous or wild-type males with wild-type females (left). Overexpression of *3xflag-sfGFP-eIF4E1b* (*eif4e1b*^{tg}) in transgenic wild-type and *eif4e1b* mutant siblings results in fertile males and females (right; ix = incross). (**F**) Percentage of dead embryos at 1-day post fertilization (dpf). Embryos were obtained by crossing homozygous or wild-type *eif4e1b*$^{\Delta 11}$ male siblings with wild-type females (left), or by incrossing (ix) wild-type or mutant fish expressing *3xflag-sfGFP-eIF4E1b* (right). (**G**) Hematoxylin and eosin (H&E, top) and phospho-histone H3 (bottom) staining of *eif4e1b* $^{\Delta 19}$ ovary sections. Scale bars = 20 µm. (**H**) Live microscopy of gonads from juvenile wild-type and *eif4e1b*$^{\Delta 11}$ mutant siblings expressing eGFP under the control of the *ziwi* promoter (scale bars = 1 mm). Fish were classified based on the area and intensity of eGFP. Numbers (top right) indicate the number of fish in each category. (**I**) Number of fish in *H* that developed into males or females. (**J**) Confocal microscopy of fixed gonads isolated from wild-type (left) and *eif4e1b*$^{\Delta 11}$ homozygous (right) juveniles expressing eGFP under the control of the *ziwi* promoter. Nuclei were stained with DAPI (in blue; Sc = spermatocytes; Sz = spermatozoa; scale bars = 100 µm). Data information: (**E, F**) lines indicate mean with SD. Significance for the first two genotypes was calculated using unpaired *t* tests. The other genotypes were compared using one-way ANOVA followed by Tukey's test (if not indicated, *P* value > 0.005).

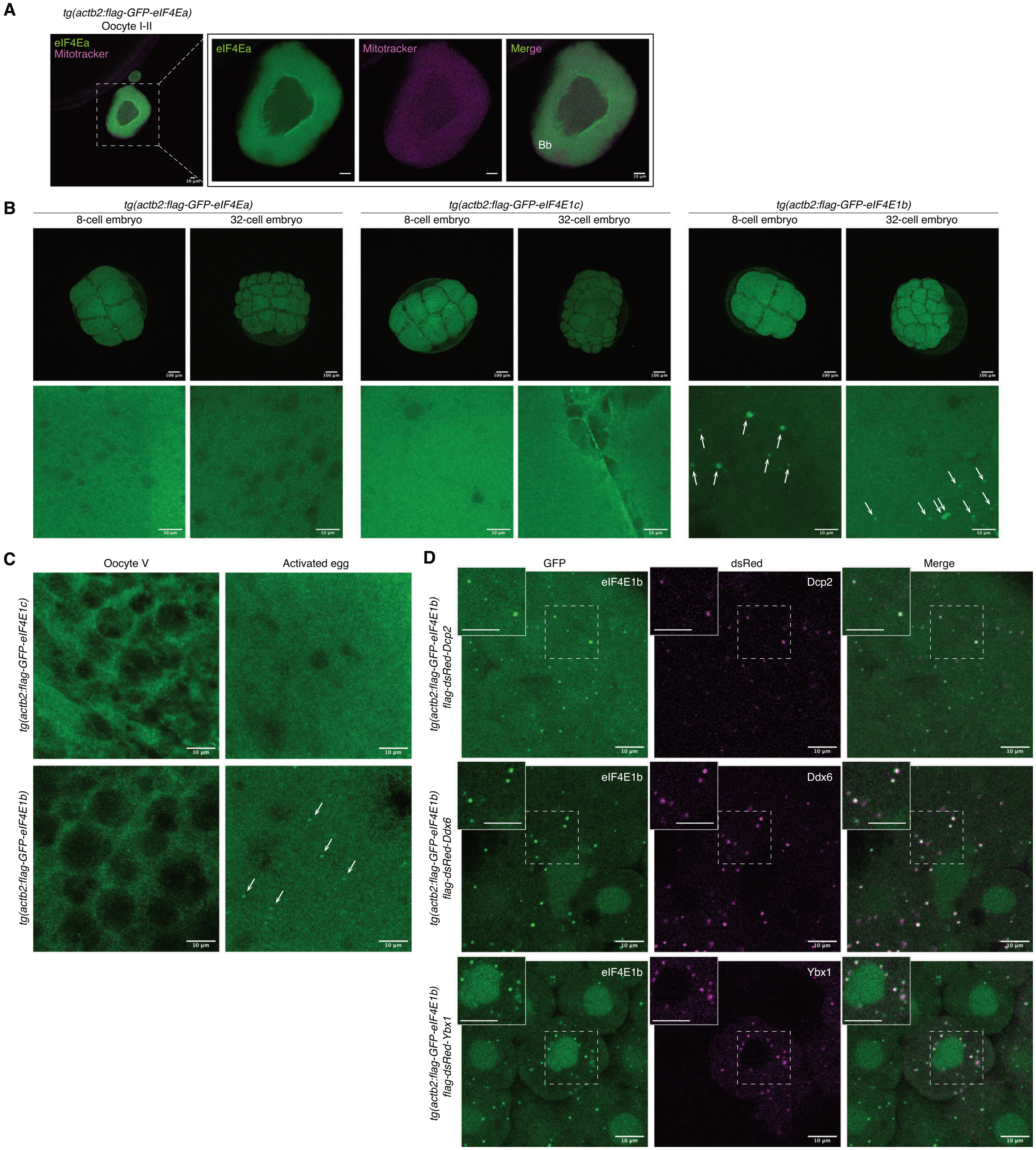

Figure EV3. eIF4E1b localizes to P-bodies in the embryo.

(**A**) eIF4Ea localizes to the cytosol of fixed early zebrafish oocytes. Mitochondria present in the Balbiani body (Bb) are stained with Mitotracker. Scale bars = 10 μm. (**B**) Confocal microscopy images of fixed transgenic embryos expressing 3xflag-sfGFP-tagged eIF4Ea, eIF4E1b and eIF4E1c (scale bars: top = 100 μm; bottom = 10 μm). eIF4E1b foci are indicated by white arrows. (**C**) Confocal microscopy images of squeezed (oocyte V) and activated eggs from transgenic lines expressing 3xflag-sfGFP-tagged eIF4E1c (top) and eIF4E1b (bottom). eIF4E1b foci are indicated by white arrows. (**D**) Colocalization experiments in zebrafish embryos expressing *3xflag-sfGFP-eIF4E1b* transiently expressing P-body markers. mRNAs containing the coding sequences of dsRed-tagged P-body components (Dcp2, Ddx6 and Ybx1) were injected into 1-cell embryos. Images were taken at 3 h post fertilization. Regions enclosed by dashed boxes are shown at higher magnification on the top left (scale bars = 10 μm).

 © The Author(s)

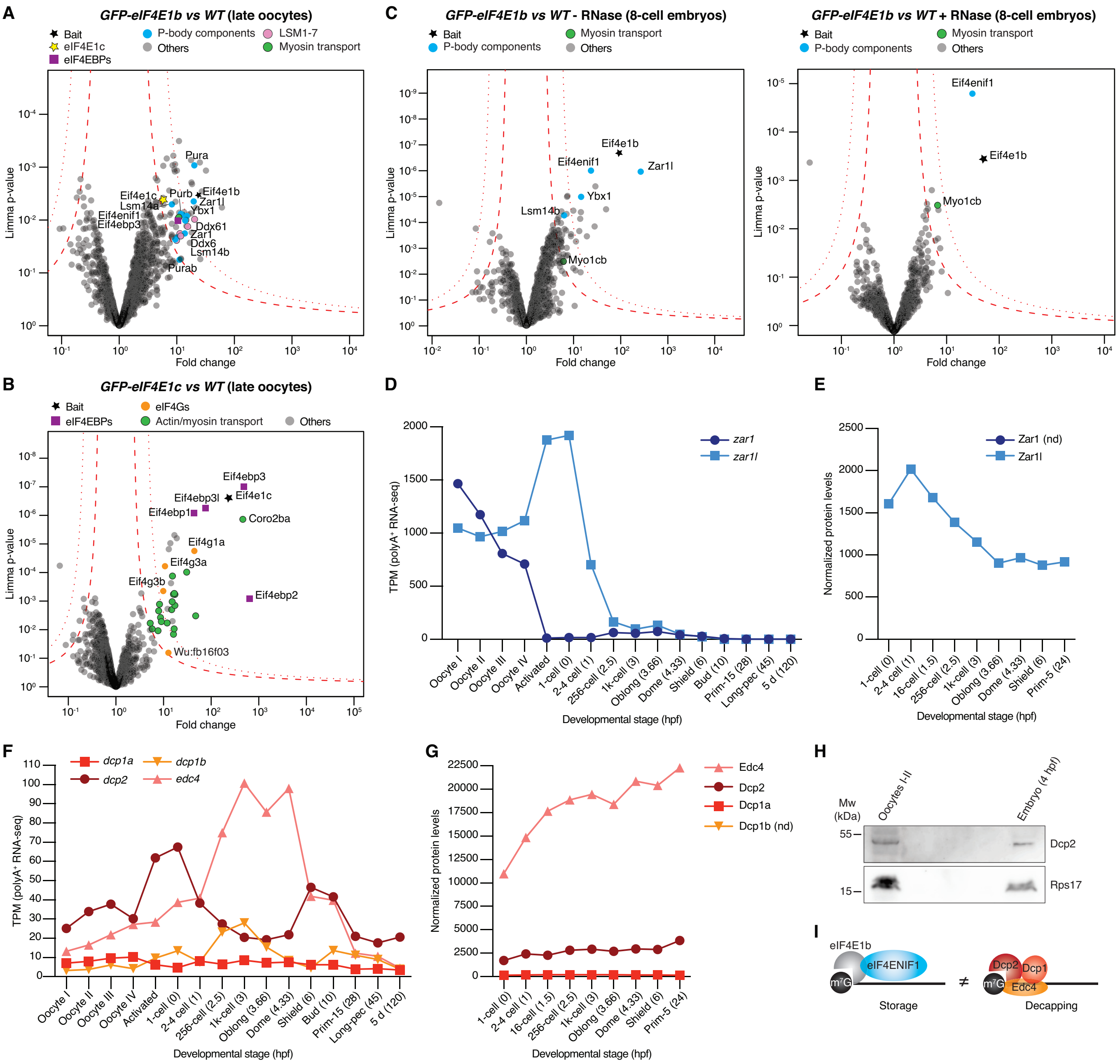

Figure EV4. Additional IP-MS and expression data.

(**A, B**) Volcano plots of the proteins identified by IP-MS in zebrafish late oocytes (stage III-IV) expressing GFP-tagged eIF4E1b (**A**) or eIF4E1c (**B**) compared to wild type (*WT*). (**C**) Volcano plots of the proteins identified by IP-MS in 8-cell embryos expressing GFP-tagged eIF4E1b compared to *WT* in the absence (mock, left) or presence (right) of RNase I. (**D**) Levels of *zar1* and *zar1l* mRNAs during zebrafish oogenesis and embryogenesis based on polyA-selected RNA-seq data (Pauli et al, 2012; Cabrera-Quio et al, 2021). (**E**) Normalized expression of Zar1l protein during zebrafish embryogenesis in TMT-MS. Zar1 peptides were not detected (nd). (**F, G**) mRNA (**F**) and protein (**G**) levels of decapping factors during zebrafish oogenesis and embryogenesis based on polyA-selected RNA-seq (Pauli et al, 2012; Cabrera-Quio et al, 2021) and TMT-MS data, respectively. (**H**) Western blot showing the expression of Dcp2 (predicted Mw of 45.5 kDa) and Rps17 (loading control, predicted Mw of 15.4 kDa) in zebrafish early oocytes and embryos. Data information: (**A–C**) statistical significance was determined using limma (Smyth, 2005). Permutation-based false discovery rates (FDRs) are displayed as dotted (FDR < 0.01) or dashed (FDR < 0.05) lines ($n = 3$ biological replicates). Hpf hours post fertilization, Mw molecular weight, TPM transcripts per million. (**I**) Model of eIF4E1b function in mRNA stability: eIF4E1b interacts with eIF4ENIF1 in P-bodies and binds to the mRNA cap (left), thereby blocking access to the decapping machinery (right), which is also located in P-bodies.

© The Author(s)

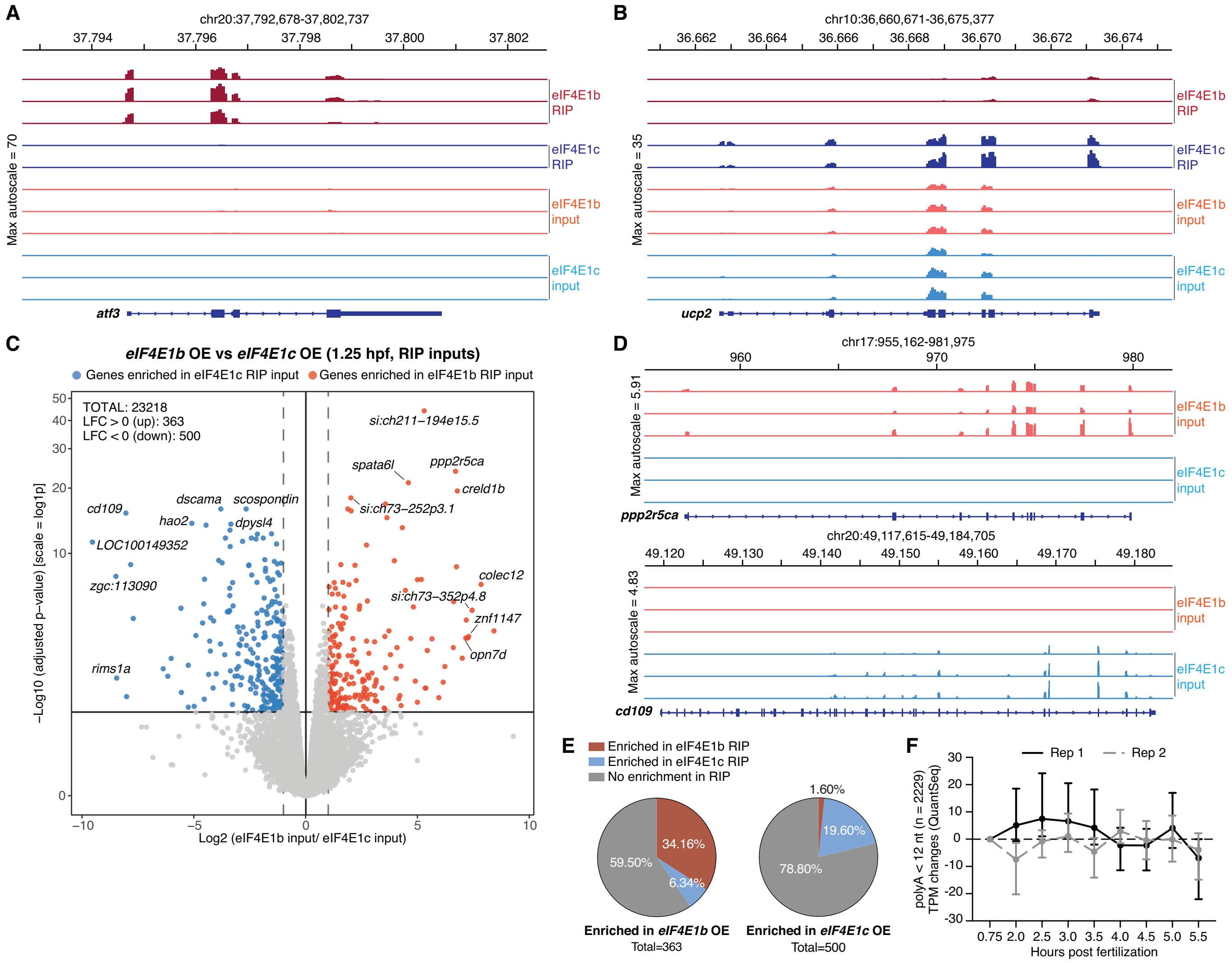

Figure EV5. Additional RNA immunoprecipitation (RIP) and RNA sequencing (RNA-seq) analyses.

(**A**, **B**) Examples of mapped RNA-seq reads of differentially expressed genes (DEGs) in eIF4E1b (**A**) and eIF4E1c (**B**) RIPs performed at 1.25 hours post fertilization (hpf). (**C**) DEG analyses of mRNAs isolated from total lysates (inputs from RIP experiments) of 8-cell stage embryos expressing 3xflag-sfGFP tagged eIF4E1b or eIF4E1c under the control of the *actb2* promoter (*eIF4E1b* or *eIF4E1c* OE, respectively). mRNAs enriched in *eIF4E1b* OE and *eIF4E1c* OE are shown in red and blue, respectively. Statistical significance was determined using Benjamini-Hochberg-corrected Wald test (DeSeq2; *P* value < 0.005; $n = 3$ biological replicates). (**D**) Mapped RNA-seq reads of example genes specifically upregulated in *eIF4E1b* (top) or *eIF4E1c* (bottom) OE 8-cell embryos. (**E**) Fraction of transcripts significantly upregulated in *eIF4E1b* OE (left) or *eIF4E1c* OE (right) embryos that were also enriched in eIF4E1b and eIF4E1c RIPs at 1.25 hpf (*P* value < 0.05). (**F**) Changes in mRNA levels (in transcripts per million, TPM) of transcripts with polyA tails containing less than 12 nucleotides during zebrafish embryogenesis according to published RNA-seq data (Bhat et al, 2023). Medians (dots) with interquartile ranges (bars) are shown.

 © The Author(s)
